# A Scalable Framework for Comprehensive Typing of Polymorphic Immune Genes from Long‐Read Data

**DOI:** 10.1002/advs.202521531

**Published:** 2026-02-11

**Authors:** Shuai Wang, Xuedong Wang, Mengyao Wang, Qian Zhou, Lusheng Wang, Shuai Cheng Li

**Affiliations:** ^1^ City University of Hong Kong Shenzhen Research Institute Shenzhen China; ^2^ Department of Computer Science City University of Hong Kong Kowloon Hong Kong China; ^3^ OmicLab Limited, New Territories Hong Kong China; ^4^ Department of Epidemiology and Health Statistics Fujian Medical University, School of Public Health Fuzhou China

**Keywords:** co‐evolution, complex gene typing, gene sequence reconstruction, immunity, long‐read sequencing

## Abstract

Long‐read sequencing promises to unravel the complexity of polymorphic immune genes including HLA, KIR, IG, and TCR, yet existing tools fall short in accuracy and scope. Here, we present SpecImmune, the first unified computational framework to simultaneously genotype these genes alongside the intricate CYP family from long‐read data. Employing an iterative graph‐based haplotype reconstruction algorithm, SpecImmune delivers precise diploid assemblies for each locus from diverse data types. Validated on 1019 samples from the 1kGP ONT cohort, 42 PacBio CLR and 9 PacBio HiFi samples from HGSVC, and 47 PacBio HiFi plus 37 ONT samples from HPRC, SpecImmune achieved 98% four‐field HLA typing accuracy, surpassing HLA*LA by 11% and SpecHLA by 12%. It also delivers robust KIR and germline IG/TCR genotyping and supports multi‐locus CYP allele detection, positioning it among the first integrated long‐read solutions across immune gene families. Beyond superior performance, SpecImmune uncovers elevated germline IG/TCR heterozygosity in African populations ($p=9.45\times 10^{-86}$) and, through 1kGP analysis, suggests widespread cross‐family co‐evolution, clustering immune genes into two functionally distinct communities: the *Integrated Immune–Metabolic Community* and the *Adaptive Presentation Community*. Additionally, it enables allele‐specific drug dosing recommendations and offers flexible customization for new loci, advancing immunology, precision medicine, and evolutionary genomics.

## Graphical abstract

SpecImmune introduces a unified computational framework optimized for long‐read sequencing to resolve over 400 highly polymorphic immune genes. This scalable approach achieves high‐resolution typing, enabling the discovery of cross‐family co‐evolutionary networks and population‐specific diversity.

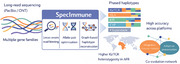

## 1 Introduction

The human immune system encompasses various gene families that are fundamental to protecting against pathogens, including the Human Leukocyte Antigen (HLA), Killer Immunoglobulin‐like Receptor (KIR), Immunoglobulin (IG), and T cell receptor (TCR) families. The HLA locus, located on the short arm of chromosome 6 (6p21.31), is the most gene‐dense and polymorphic region in the human genome [1]. Variants frequently occur in the antigen‐binding sites of these molecules, which are critical for their interactions with TCRs and KIRs, enabling the immune system to recognize a wide array of antigens. The KIR gene family on chromosome 19q13.4 also exhibits significant allelic polymorphism, consisting of 15 genes and two pseudogenes. Particularly, exons 3‐5 of KIR genes, responsible for encoding the immunoglobulin‐like domains crucial for binding to HLA molecules, exhibit elevated levels of nonsynonymous mutations [2, 3, 4]. Inhibitory KIRs are essential for maintaining self‐tolerance by recognizing self‐HLA class I molecules and inhibiting natural killer (NK) cell activity [5]. In contrast, activating KIRs detect altered‐self or non‐self antigens, triggering NK cell‐mediated immune responses [6]. The stochastic expression of activating and inhibitory KIRs results in a highly diverse NK cell population [6]. The IG genes encode B cell receptors (BCRs), which recognize antigens and initiate antibody production. The TCR genes encode T cell receptors, enabling T cells to recognize antigen‐HLA complexes presented by antigen‐presenting cells [7]. Both IG and TCR genes undergo V(D)J recombination during lymphocyte development, where variable (V), diversity (D), and joining (J) gene segments are rearranged to generate an extensive repertoire of receptors [7, 8]. This diversity is essential for enabling the immune system to recognize and respond to various antigens. The cytochrome P450 (CYP) enzyme family, typically associated with drug metabolism, is also essential in immune regulation. CYPs metabolize various compounds that can influence immune responses, including drugs, toxins, and endogenous molecules [9, 10]. In cancer patients, CYP activity is often reduced, and the administration of anti‐PD‐1 antibodies–a form of immune checkpoint inhibitor therapy–can further impair CYP‐mediated metabolism [11, 12]. This reduction in CYP activity may affect the immune system's ability to eliminate tumors by altering the pharmacokinetics of immunotherapies and other treatments. Interestingly, studies suggest that enhancing CYP activity could improve the effectiveness of cancer immunotherapies by optimizing drug metabolism and reducing immune suppression [11, 12].

Different immune‐related gene families have a complex interplay relationship. The combined diversity of IG, TCR, and HLA molecules forms the foundation of both humoral and cellular immune responses [13]. HLA class I molecules present intracellular antigens to CD8+ T cells, while HLA class II molecules present extracellular antigens to CD4+ T cells [14]. B cells internalize antigen‐BCR complexes, process the antigens, and present these as antigen‐HLA complexes to T helper cells. The recognition of these complexes by BCRs and TCRs is driven by V(D)J recombination during lymphocyte development [15, 16, 17]. Concurrently, NK cells rely on diverse KIRs to interact with HLA class I molecules, which regulate NK cell‐mediated cytotoxicity. The specific interactions between KIRs and TCRs with the peptide‐HLA class I complex depend highly on the peptide sequence, emphasizing the importance of peptide specificity in immune recognition [18]. The interplay between KIR and HLA gene families has also been shown to influence susceptibility to autoimmune diseases and to impact the success of hematopoietic stem cell transplantation [14]. The impact of CYPs on immune function through drug metabolism and immune modulation underscores the intricate interplay between CYPs and other immune‐related gene families in shaping immune responses and treatment outcomes [12, 19]. Accurate typing of these gene families provides a critical foundation for understanding their complex interplay and for elucidating the genetic basis of immune function and disease susceptibility.

High polymorphism, structural variants (SVs), and inter‐locus homology confound genotyping these immune‐related genes, particularly with short‐read next‐generation sequencing. Short reads struggle with variant phasing over long distances, accurate mapping in repetitive regions, and SV detection, leading to ambiguities in allele assignment. Computational tools like OptiType [20], PolySolver [21], and SpecHLA [22] have advanced short‐read HLA typing through alignment optimization or graph‐based approaches, while methods like KIR*IMP [23], ImmunoTyper‐SR [24], and Cyrius [25] address KIR, IG/TCR, and *CYP2D6*, respectively. However, these are often gene‐specific, computationally intensive, or limited in resolution.

Long‐read technologies, such as Pacific Biosciences (PacBio) high‐fidelity (HiFi) and continuous long reads (CLR), and Oxford Nanopore Technologies (ONT), offer transformative potential by spanning full loci and providing phase information [26, 27]. Software packages such as GenDX NGSengine, HLA*LA [28], SpecHLA [22], and pbaa have been introduced for HLA typing. For *CYP2D6* genotyping, the tool pangu is designed specifically for PacBio HiFi data, while the pipeline PLASTER supports *CYP2D6* typing from PacBio SMRT amplicons [29]. Despite their utility, these tools face limitations such as high computational costs, dependence on specific sequencing platforms, and a narrow focus on single gene families. Moreover, long‐read immune gene typing remains fundamentally challenging because high base‐level read error rates, extreme allelic polymorphism, and extensive inter‐locus homology jointly confound read alignment, variant calling, haplotype phasing, and diploid assembly. These challenges underscore the need for versatile and efficient computational methods that can handle diverse complex gene families and leverage the full potential of long‐read sequencing data.

To bridge this gap, we developed SpecImmune, an open‐source framework that integrates read binning, allele selection, and iterative graph‐based haplotype reconstruction to genotype complex immune‐related genes from long‐read WGS and targeted amplicons. SpecImmune employs locus‐level read binning to mitigate cross‐locus homology, applies an optimization‐based strategy to identify the best‐matching allele pair for accurate read alignment and variant calling, and subsequently reconstructs personalized haplotypes through an iterative graph‐based approach, enabling robust and accurate genotyping across diverse data qualities. SpecImmune demonstrated superior accuracy in typing HLA, KIR, IG, TCR, and CYP loci in five long‐read sequencing datasets: 1,019 ONT sequencing samples from the 1000 Genomes Project (1kGP) cohort [30, 31], 47 PacBio HiFi samples and 37 ONT samples from the Human Pangenome Reference Consortium (HPRC) project [32], 42 PacBio CLR samples, and 9 PacBio HiFi samples from the Human Genome Structural Variation Consortium (HGSVC) project [33]. Moreover, SpecImmune reveals novel patterns of heterozygosity and cross‐family co‐evolution that illuminate immune evolution and inform clinical applications. SpecImmune is freely available at https://github.com/deepomicslab/SpecImmune.

## 2 Results

### 2.1 Overview of SpecImmune

**FIGURE 1:**
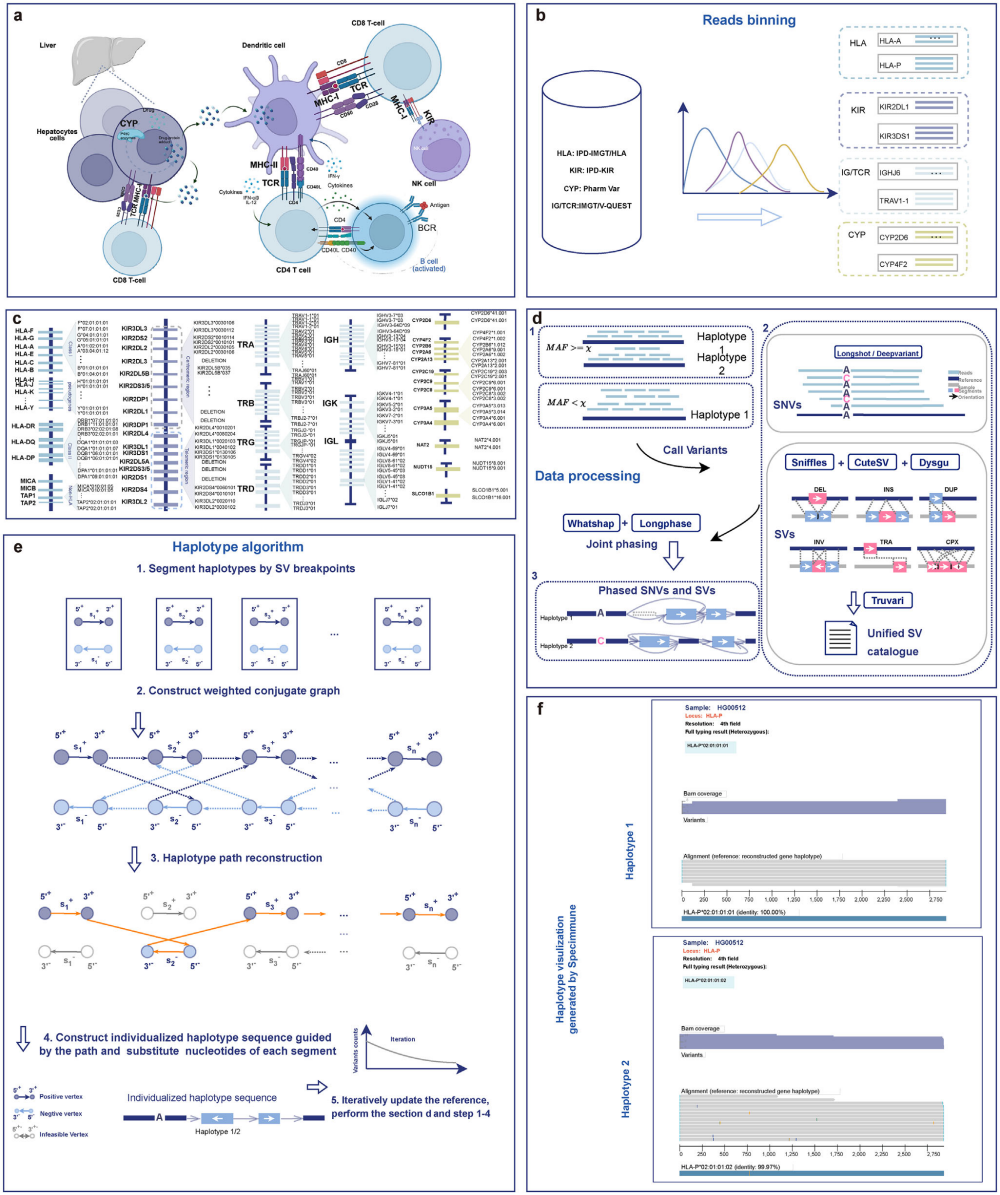
Workflow of SpecImmune. (a) Interactions among HLA, KIR, IG, TCR, and CYP in immunity and metabolism. (b) Reads are binned into gene loci by mapping to the allele database. (c) The best‐matched allele pair is identified, and reads are assigned to alleles. *Note:* The loci shown here correspond to gene class clusters rather than their actual positions in the genome. (d) Allele‐specific reads are mapped to the best‐matched allele to construct haplotypes, followed by variant calling and phasing. (e) The personalized haplotype sequence is iteratively constructed using the *haplotype reconstruction* algorithm [36, 37] (steps 1–5). (f) An example of the visualization report generated by SpecImmune. The figure demonstrates the heterozygous *HLA‐P* gene of sample HG00512, showing reads aligned to the reconstructed allele sequence (middle gray track), alignment coverage (top gray‐blue distribution), and the haplotype‐specific top matches from the IMGT database (bottom blue track).

Given long‐read WGS data aligned to the hg38 reference, SpecImmune initially extracts reads from specific regions of interest (HLA, KIR, IG, TCR, CYP) (Figure 1a,b). This step is skipped for targeted amplicon sequencing data. Subsequently, SpecImmune further bins extracted reads to their respective gene loci by aligning them to the allele database encompassing all alleles within the region of interest. Next, SpecImmune aligns the binned reads to all alleles at each respective gene locus in the database. These alignments for every read are preserved for subsequent analysis. For each gene locus, SpecImmune records the alignment identity and the number of matching DNA bases for each read aligned to each allele. It then evaluates all possible allele pairs by computing the total alignment identity and the number of matching bases from all reads for the locus. The allele pair that maximizes alignment identity and matching base count across all binned reads is selected as the best‐matched for the locus (Figure 1c).

SpecImmune subsequently reconstructs personalized haplotype sequences by leveraging reads aligned to the best‐matched allele pair. First, single‐nucleotide variants (SNVs) and structural variants (SVs) are detected based on the alignment. Joint phasing of SNVs and SVs is then performed using WhatsHap v2.3 [34] and Longphase v1.7.3 [35]. Based on the identified variants, linear haplotype sequences are reconstructed by applying the haplotype reconstruction algorithm [36, 37] to resolve non‐linear connections. This haplotype reconstruction process is iteratively refined, with haplotypes updated until no new variants are detected. A sliding‐window approach is then used to mask low‐depth regions, ensuring the generation of high‐quality personalized diploid haplotype sequences (Figure 1d,e). Finally, SpecImmune aligns each reconstructed sequence to the allele database and identifies the most matching alleles for the nomenclature of the sequence. To ensure the reliability of the typing results, the Specimmune generates a comprehensive visualization report, as exemplified in Figure 1f. In this IGV‐like interface, allele‐specific reads are aligned directly to their best‐matching reference alleles identified from the database. The visualization integrates metrics including the precise HLA typing resolution level, zygosity status, and the quantitative sequence identity percentage. Furthermore, it explicitly highlights mutational patterns relative to the reference genome. This granular view allows researchers to inspect read pileups and variant calls in context, thereby significantly facilitating the manual verification of putative novel alleles and the rigorous evaluation of overall typing confidence.

Moreover, SpecImmune is the most comprehensive method to type immune‐related genes from various sequencing settings (Table 1). All targeted gene loci analyzed by SpecImmune are listed in Table S5 (Supporting Information). The gene list is dependent on the reference database and may vary across different database versions. The nomenclature schemes for HLA/CYP/KIR/IG/TCR alleles are depicted in Figures S1 and S2 (Supporting Information). The showcase of SpecImmune typing results for the sample HG00377 from the 1kGP is depicted in Figure S3 (Supporting Information). Furthermore, SpecImmune is computationally efficient in typing immune‐related gene families (Note S1.1 and Figures S4 and S5, Supporting Information), and can be executed on a standard personal computer. The following sections showcase its typing accuracy and practical application in real‐world data analysis.

**TABLE 1:** Functionalities of state‐of‐the‐art tools in typing immune‐related genes from long‐read data. √ indicates the software has the feature. means whole genome sequencing.

Methods	HiFi	CLR	ONT	Amplicon	${\mathrm{WGS}}^{*}$	RNA‐Seq	HLA	KIR	IG	TCR	CYP
SpecImmune	√	√	√	√	√	√	√	√	√	√	√
SpecHLA [22]	√	√	√	√	√		√				
HLA*LA [28]	√	√	√	√	√		√				
NGSengine	√	√	√	√	√	√	√				
pbaa	√			√			√	√			
PLASTER [29]	√			√							√
pangu	√			√							√

### 2.2 High‐Resolution and Comprehensive HLA Typing Using Long‐Read Technologies

SpecImmune enables accurate four‐field HLA typing across various long‐read sequencing protocols, supports RNA‐seq data, and expands coverage to a larger set of HLA genes compared to existing methods. It identifies 39 MHC‐region genes from the IMGT database, including 6 HLA class I genes, 12 HLA class I pseudogenes, 17 HLA class II genes, and 4 non‐HLA genes. To validate SpecImmune for HLA typing, we compared SpecImmune with SpecHLA and HLA*LA in five real long‐read datasets (Table 2). The GenDX NGSengine (https://www.gendx.com/product_line/ngsengine/) software is excluded as it is not freely available. The benchmark datasets include 47 PacBio HiFi samples and 37 ONT samples from the HPRC project, 42 PacBio CLR samples and 9 PacBio HiFi samples from the HGSVC project, and 1,019 ONT sequencing samples from the 1kGP cohort [31]. The specific command parameters for SpecHLA, HLA*LA, and SpecImmune are outlined in Table S1 (Supporting Information).

**TABLE 2:** Summary of benchmark datasets in validation of SpecImmune. Due to varying read coverage among gene families in each sample, and the unreliable inference of ground truth for certain gene families in specific samples, the number of samples utilized differs between gene families.

Dataset	Sequencing protocol	Sequencing strategy	DNA/RNA	Phased assembly available	No. of samples
1kGP	ONT	WGS	DNA	No	1,019
HPRC HiFi	PacBio HiFi	WGS	DNA	Yes	47
HPRC ONT	ONT	WGS	DNA	Yes	37
HGSVC HiFi	PacBio HiFi	WGS	DNA	Yes	9
HGSVC CLR	PacBio CLR	WGS	DNA	Yes	42
*CYP2D6* Amplicon	PacBio HiFi	Targeted	DNA	No	10
RNA 1	Iso‐seq	RNA‐Seq	RNA	No	3
RNA 2	MAS‐seq	RNA‐Seq	RNA	No	3
RNA 3	ONT direct RNA	RNA‐Seq	RNA	No	3
Total	—	—	—	—	1,173

**FIGURE 2:**
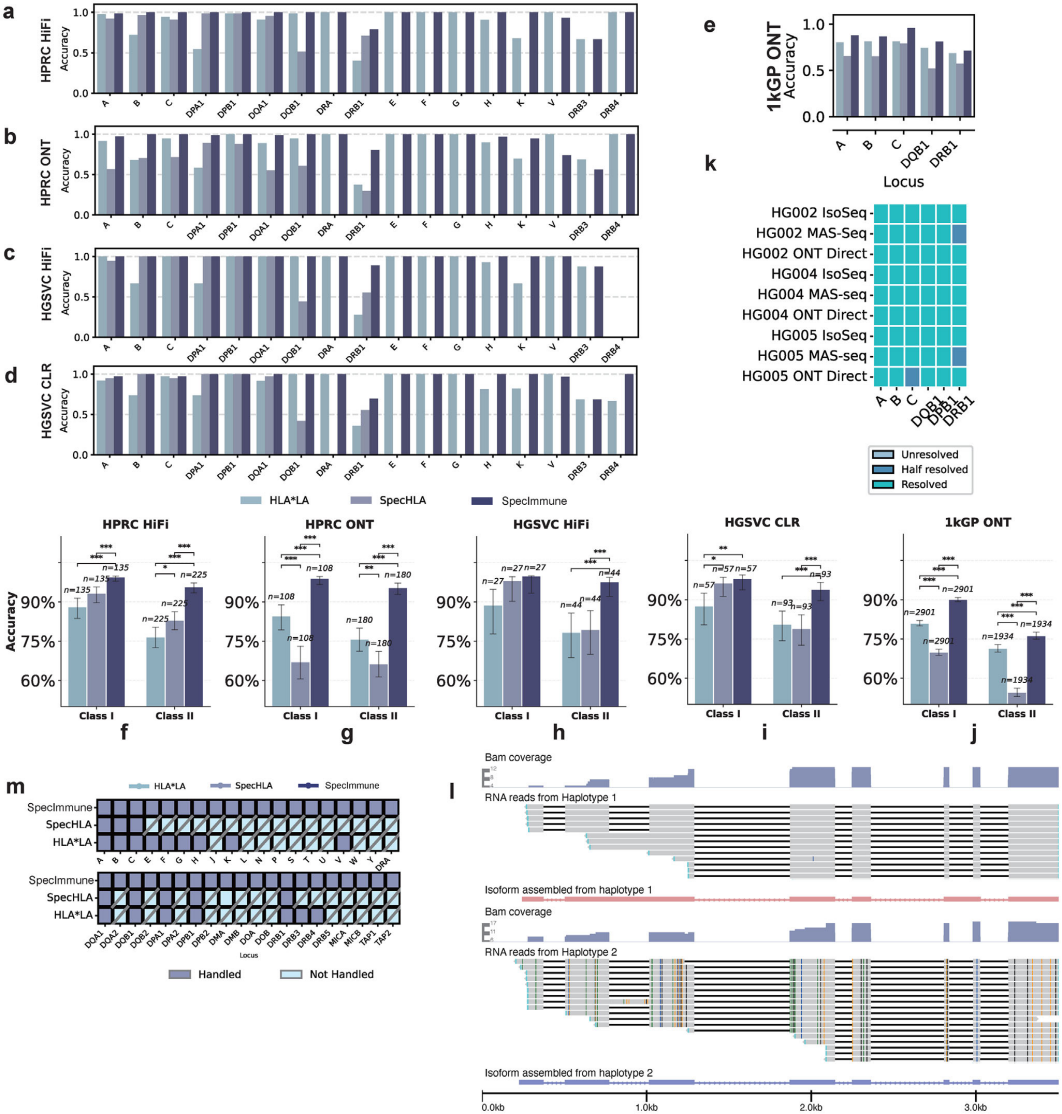
Evaluation of SpecImmune for typing HLA genes. (a–d) Accuracy comparisons among SpecHLA, HLA*LA, and SpecImmune, across common HLA loci of HLA*LA, SpecHLA and SpecImmune for the HPRC HiFi (a), HPRC ONT (b), HGSVC HiFi (c), and HGSVC CLR (d) datasets, respectively. (e) Accuracy comparisons across the *HLA‐A*, *HLA‐B*, *HLA‐C*, *HLA‐DQB1*, and *HLA‐DRB1* loci within the 1kGP ONT dataset. (f–j) Statistical significance of performance differences across the five datasets: HPRC HiFi (f), HPRC ONT (g), HGSVC HiFi (h), HGSVC CLR (i), and 1kGP ONT (j). P‐values were calculated using McNemar's test and adjusted for multiple comparisons using the Benjamini‐Hochberg (FDR BH) method. Asterisks indicate statistical significance levels: ∗ *p* < 0.05, ∗∗ *p* < 0.01, and ∗∗∗ *p* < 0.001. (k) Performance of SpecImmune across various long‐read RNA‐Seq technologies. Resolved indicates that both haplotypes of the locus were successfully typed, Half resolved indicates that only one of the two haplotypes was successfully typed, and Unsolved indicates that neither haplotype was successfully typed. (l) Phased long RNA‐Seq reads alongside allele‐specific isoform assemblies for *HLA‐A* in the HG002 sample. The first track shows the reads coverage of haplotype 1, the second track shows the alignment of haplotype 1 reads, and the third track shows the assembled RNA isoform expressed from haplotype 1. The fourth track shows the reads coverage of haplotype 2, the fifth track shows the alignment of haplotype 2 reads, and the sixth track shows the assembled RNA isoform expressed from haplotype 2. (m) Ability to handle HLA genes among the three methods.

SpecImmune consistently demonstrated the highest accuracy at the four‐field HLA typing, outperforming the other tools in every instance in the HPRC HiFi, HPRC ONT, HGSVC HiFi, and HGSVC CLR datasets. In the HPRC HiFi dataset, SpecImmune achieved an average typing accuracy of 98% (2361/2420), outperforming SpecHLA at 87% (626/720) and HLA*LA at 87% (1145/1320). In the HPRC ONT dataset, SpecImmune reached 98% (1865/1946) accuracy, while SpecHLA and HLA*LA achieved 69% (386/592) and 86% (910/1058), respectively. For the HGSVC CLR dataset, SpecImmune recorded an accuracy of 95% (1101/1154), with SpecHLA and HLA*LA reaching 86% (257/300) and 86% (500/570), respectively. In the HGSVC HiFi dataset, SpecImmune attained 98% (524/536) accuracy, compared to 87% (123/142) for SpecHLA and 88% (231/262) for HLA*LA (Figure 2a–d; Figure S28, Supporting Information). The number of processed gene loci varied among the tools, and we calculated the accuracy based on all processed gene loci for each tool. Moreover, we categorized the HLA genes into four classes: Class I, Class I pseudogenes, Class II, and Non‐HLA. Subsequently, we computed the accuracy for each class individually. In these four datasets, SpecImmune achieved typing accuracies of 99% (1007/1010), 96% (1088/1132), 93% (2034/2182), and 82% (551/674) across the four gene classes on average. In comparison, SpecHLA reached 88% (442/504) accuracy for class I genes and 76% (640/838) for class II genes. HLA*LA attained accuracies of 93% (944/1010), 80% (218/272), and 82% (886/1086) for class I, class I pseudogenes, and class II genes, respectively (Figure 2a–d). For each HLA gene class, SpecImmune demonstrated superior accuracy compared to the other two software tools. Additionally, SpecImmune consistently achieved higher accuracy for each HLA gene than the other two methods (Figures S6–S9, Supporting Information). Across the four datasets, after filtering out loci with sequencing depth below 10x, SpecImmune achieved an average HLA typing accuracy of 98%, representing an 11% improvement over HLA*LA and a 12% improvement over SpecHLA.

To ensure a rigorous statistical comparison, we evaluated accuracy on the intersection of samples successfully typed by all tools (common samples) and applied McNemar's test for significance (Figure 2f–j). In the HPRC HiFi dataset, within the common subset of samples typed by both SpecImmune and HLA*LA (n=660), SpecImmune achieved significantly higher accuracy (97.6%) compared to HLA*LA (86.7%; McNemar's $p<10^{-26}$). Similarly, in the intersection with SpecHLA (n=360), SpecImmune (97.2%) significantly outperformed SpecHLA (86.9%; McNemar's $p<10^{-13}$). In the HPRC ONT dataset, SpecImmune maintained its superiority. On the common samples shared with HLA*LA (n=529), SpecImmune achieved 95.8% accuracy versus 86.0% for HLA*LA ($p<10^{-15}$). Against SpecHLA (n=288), SpecImmune achieved 96.9% accuracy compared to 66.7% for SpecHLA ($p<10^{-31}$). Consistent results were found in the HGSVC datasets. In the HGSVC HiFi dataset, SpecImmune achieved 98.9% accuracy compared to HLA*LA's 88.2% (n=131; $p<10^{-6}$) and 98.6% compared to SpecHLA's 86.6% (n=71; $p<10^{-3}$). In the HGSVC CLR dataset, SpecImmune significantly outperformed HLA*LA (96.7% vs 87.7% on n=285; $p<10^{-11}$) and SpecHLA (95.7% vs 85.7% on n=150; $p<10^{-5}$). These results demonstrate that SpecImmune significantly outperforms both SpecHLA and HLA*LA across all tested datasets.

Further, among the three tools, SpecImmune again demonstrated the highest accuracy in the intermediate‐depth 1kGP ONT dataset. In this dataset, our evaluation focused on two‐field typing for *HLA‐A*, ‐B, ‐C, ‐DQB1, and ‐DRB1 genes. We filtered out low‐quality alignments and genes with an average depth below 10x. SpecImmune achieved accuracies of 94% (1134/1208), 89% (1402/1568), 98% (1561/1590), 85% (1209/1422), and 73% (1348/1858) across these five genes (Figure 2e). Statistical analysis on the common samples in the 1kGP ONT dataset confirmed these findings. SpecImmune significantly outperformed HLA*LA on the intersection of all genes (n=4,835), achieving 84.6% accuracy compared to 77.2% ($p<10^{-38}$). The difference was even more pronounced against SpecHLA (n=4,835), where SpecImmune achieved 84.6% vs 63.8% ($p<10^{-245}$). We also evaluated the accuracy of these tools at the G/P group resolution level (Table S4, Supporting Information). SpecImmune achieved the highest average accuracy at 88.0%, followed by HLA*LA at 86.6%.

Moreover, SpecImmune supports HLA typing from a variety of long‐read RNA‐seq technologies, including PacBio Iso‐Seq, ONT 2D cDNA‐seq, and Direct RNA‐seq, enabling allele‐specific isoform assembly and quantification. We validated its performance using nine datasets from the GIAB project [38], achieving an average accuracy of 97% (105/108) at the G group resolution (Figure 2k). Figure 2l illustrates that long‐read RNA‐seq reads can span all the exons of *HLA‐A* and SpecImmune accurately assembled the isoform. We further showed that SpecImmune is robust against RNA degradation (Figure S27 and Note S1.8, Supporting Information).

Additionally, SpecImmune is the most comprehensive long‐read‐based tool capable of handling the majority of HLA genes. As shown in (Figure 2m), SpecImmune can process 39 genes across four gene classes within the MHC region. In contrast, SpecHLA is limited to 8 genes from HLA class I and HLA class II, while HLA*LA is confined to 17 genes across HLA class I, HLA class I pseudogenes, and HLA class II. In all of the HPRC HiFi, HPRC ONT, HGSVC HiFi, and HGSVC CLR datasets, SpecImmune demonstrated the highest average accuracy for the eight genes that all three tools could process and achieved 94% (2395/2542) accuracy for the 22 genes that only SpecImmune could handle. To date, SpecImmune is the tool that can handle most of the HLA genes from long‐read data. HLA typing results from SpecImmune on benchmark datasets are provided in Table S6 (Supporting Information).

### 2.3 First Method for Accurate KIR Typing Using Long‐Read Sequencing

**FIGURE 3:**
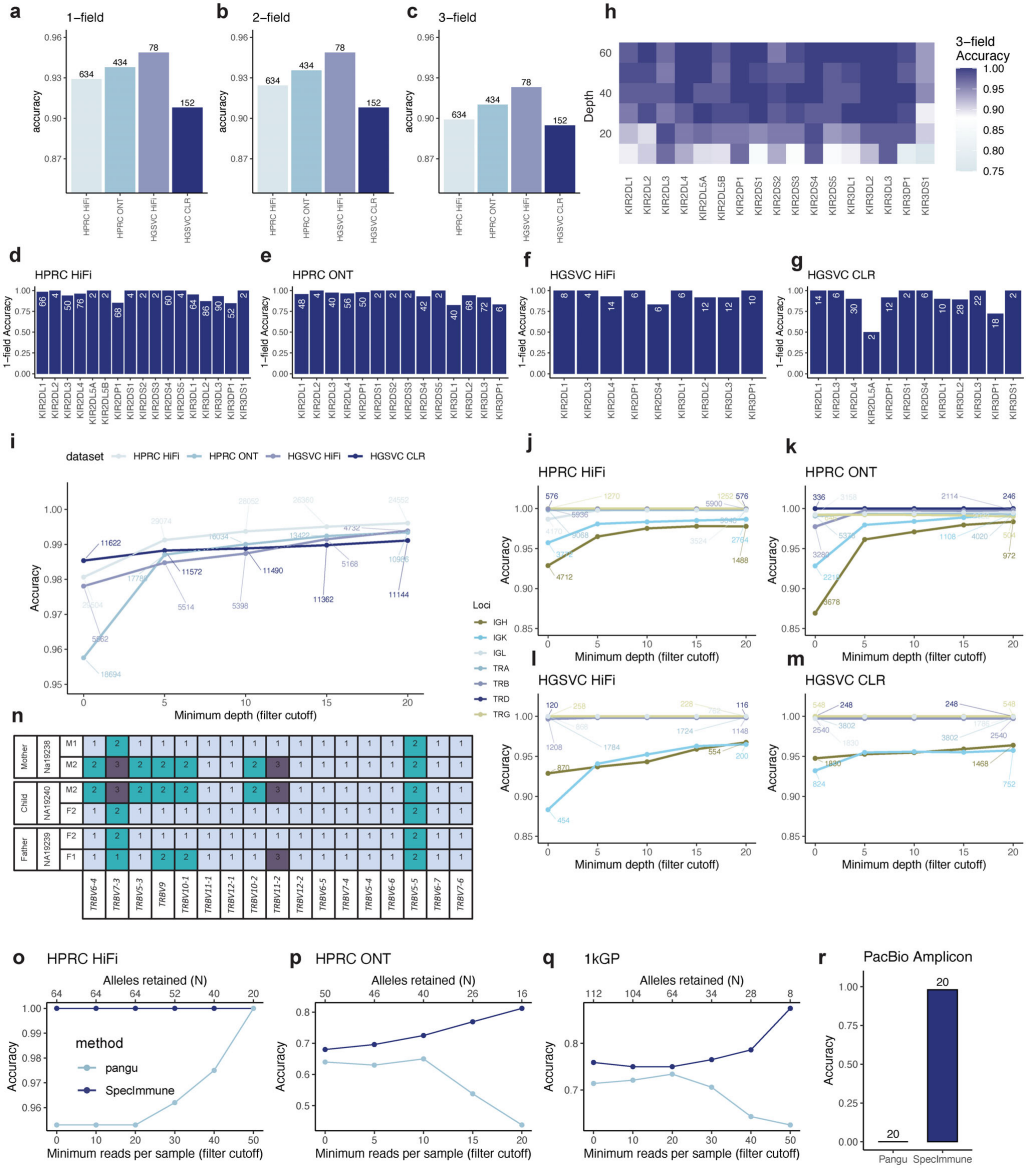
Evaluation of SpecImmune for typing KIR, IG/TCR, and CYP genes. (a–c) SpecImmune's KIR typing accuracy in four real datasets at 1‐field (a), 2‐field (b), and 3‐field (c) resolutions. The number of alleles used to calculate accuracy is shown above each bar. (d–g) The 1‐field typing accuracy at each KIR locus in the HPRC HiFi (d), HPRC ONT (e), HGSVC HiFi (f), and HGSVC CLR (g) datasets. The count of total detected alleles at each KIR locus is marked in each accuracy bar. (h) Accuracy of 3‐field KIR typing at various depth levels in simulated data, with 50 replicates per parameter combination. (i) Average typing accuracy of all IG/TCR genes with varying minimum depth cutoffs. The number of alleles used to calculate accuracy is shown for each data point. (j–m) Typing accuracy of different IG/TCR gene groups with varied sequencing depth cutoffs in the HPRC HiFi (j), HPRC ONT (k), HGSVC HiFi (l), and HGSVC CLR (m) datasets. The number of alleles used to calculate accuracy is shown for each data point. (n) Illustration of the IG/TCR loci phase block in two family trios. Numbers represent different alleles at each locus. (o‐q) Typing accuracy of *CYP2D6* by SpecImmune and pangu in the HPRC HiFi (o), HPRC ONT (p), and 1kGP (q) datasets. The bottom x‐axis shows the minimum read‐count cutoff. Accuracy at each cutoff was calculated using only samples with read counts ≥ the cutoff. The top x‐axis indicates the number of alleles contributing to the accuracy calculation. (r) Typing accuracy of *CYP2D6* by SpecImmune and pangu in HiFi amplicon data. Number of alleles to calculate the accuracy are above each bar.

SpecImmune demonstrated accurate and robust typing of KIR genes in four real datasets and one simulated dataset. The real datasets include 45 HPRC HiFi, 26 HPRC ONT, 9 HGSVC HiFi, and 19 HGSVC CLR WGS samples (Table 2). The published phased assemblies of these samples aided in inferring the reference allele types (Methods). SpecImmune achieved 1‐field KIR typing accuracies of 92.9% (589/634) in HPRC HiFi, 93.8% (407/434) in HPRC ONT, 94.9% (74/78) in HGSVC HiFi, and 90.8% (138/152) in HGSVC CLR datasets (Figure 3a–c). The highest accuracy was attained in the HGSVC HiFi dataset. While accuracy slightly declines with higher resolution, SpecImmune maintains high accuracy even at the 3‐field level, achieving 90.0% (570/634), 91.0% (395/434), 92.3% (72/78), and 89.5% (136/152) in the respective datasets. SpecImmune exhibited higher accuracy in the HPRC ONT dataset than in the HPRC HiFi dataset; however, the former has limited sequencing reads for the framework gene *KIR3DP1*. Figure 3d–g showcases the allele count and 1‐field accuracy of each locus inferred by SpecImmune in each dataset, revealing that the framework genes *KIR3DL3*, *KIR3DP1*, *KIR2DL4*, and *KIR3DL2* had more alleles detected compared to other KIR genes, with *KIR3DP1* showing particularly low counts in the HPRC ONT dataset. Additionally, we assessed the proportion of samples with at least five reads in the *KIR3DP1* locus in each dataset, yielding proportions of 88.9%, 32.4%, 100%, and 84.2% in the HPRC HiFi, HPRC ONT, HGSVC HiFi, and HGSVC CLR datasets, respectively. This suggests potential lower sequencing sensitivity for *KIR3DP1* with ONT data. SpecImmune demonstrated the lowest accuracy in the HGSVC CLR dataset. However, increasing the read cutoff significantly enhanced the accuracy of this dataset. At the 3‐field level, accuracy in the HGSVC CLR dataset surged to 92.4% with a minimum of 20 reads.

To assess SpecImmune's ability to resolve SVs, including gene fusions and CNVs, we compared it with previously reported KIR annotations in the HPRC dataset [39]. First, SpecImmune showed strong concordance with previous annotations, achieving 83% consistency (529 out of 639 alleles) in allele‐level annotations for samples with intermediate‐depth depth (≥5×) and excluding those with SVs. The consistency improved with increasing depth thresholds, reaching 100% at a minimum coverage of 30x (Figure S10a, Supporting Information). Notably, SpecImmune correctly identified two of the three previously reported gene fusion events, including the recurrent *KIR2DS4/3DL1* fusion in samples HG02630 and NA19240. It correctly annotated 5 out of 6 *KIR2DL4* deletion cases and fully captured the known deletion of the *KIR3DL2* framework gene in HG03540. For CNV analysis at the *KIR3DL1/S1* and *KIR3DL2* loci, SpecImmune achieved 84% concordance (134 out of 159 cases) with previous reports, underscoring the robustness and reliability of SpecImmune for comprehensive KIR haplotype characterization.

To further assess the accuracy of SpecImmune to resolve SVs, we compared SpecImmune's allele and SV annotations with those derived from short‐read data in the 1kGP cohort, as reported by Norman et al. [40]. A total of 781 samples overlapped between our ONT dataset and the short‐read‐based KIR cohort. Among alleles not impacted by SVs, we observed 87% concordance (1731 out of 1993 alleles) using a minimum depth threshold of 5x. Moreover, SpecImmune reliably detected gene fusion events consistent with prior findings: we identified all 16 *KIR3DL1/L2* gene fusion events, including well‐characterized alleles such as KIR3DL1*059, *060, and *061 [41]. In terms of CNV detection within *KIR3DL1/S1*, SpecImmune recovered 7 of 21 reported duplications and 31 of 51 deletions. These lower concordance rates for CNVs were primarily attributable to extremely low coverage in the *KIR3DL1* and *KIR3DL2* regions in the 1kGP ONT dataset, as illustrated in Figure S10b (Supporting Information). Despite these limitations, the high allele‐level consistency and accurate gene fusion detection underscore the strength of SpecImmune in resolving complex KIR variation from long‐read data.

Moreover, simulated data has demonstrated that SpecImmune excels in accurately typing each KIR gene. To carry out this evaluation, we simulated a dataset with sequencing depths ranging from 10x to 60x, with increments of 10x. Each depth setting was replicated 50 times, resulting in 3000 samples. The data were simulated using PBSIM3 [42], and the sequencing accuracy was set at 90%. At a sequencing depth of 10x, the accuracy for each gene was relatively lower compared to higher depths (Figure 3h). The accuracies among the genes ranged from 75% to 98%, with an average accuracy of 90.0% across all genes at this depth. As the sequencing depth increased, so did the accuracy. For depths exceeding 10x, the accuracy for all genes consistently remained at or above 90%. By the time the sequencing depth reached 60x, the average accuracy for all genes peaked at an impressive 98.9%. The evaluation in simulated data underscores the robustness and reliability of SpecImmune in KIR gene typing tasks. KIR typing results from SpecImmune on benchmark datasets are provided in Tables S7 and S8 (Supporting Information).

### 2.4 Accurate Germline IG/TCR Genotyping from Long‐Read Data using SpecImmune

SpecImmune accurately infers germline IG and TCR types and can infer haplotypes across different gene loci. SpecImmune enables accurate germline IG and TCR genotyping and infers haplotypes across gene loci. We validated SpecImmune using 45 HPRC HiFi, 26 HPRC ONT, 9 HGSVC HiFi, and 19 HGSVC CLR samples (Table 2), with ground truth alleles derived from phased genome assemblies (Methods). SpecImmune achieved high IG and TCR typing accuracies of 98.1% (28 933/29 504), 95.8% (17 901/18 694), 97.8% (5440/5562), and 98.5% (11 452/11 622) in the HPRC HiFi, HPRC ONT, HGSVC HiFi, and HGSVC CLR datasets, respectively (Figure 3i). SpecImmune exhibited lower accuracy with ONT data than PacBio HiFi and CLR data. As sequencing depth increases, the accuracy in these datasets also improves. With a minimum depth of 20x, in the HPRC HiFi, HPRC ONT, HGSVC HiFi, and HGSVC CLR datasets, SpecImmune achieved IG and TCR typing accuracies of 99.6% (24 456/24 552), 99.3% (10 914/10 986), 99.4% (4703/4732), and 99.1% (11 045/11 144), respectively. The accuracy in all four datasets exceeds 99% with a minimum depth of 20x. Furthermore, we classified IG and TCR loci into IGH, IGK, IGL, TRA, TRB, TRD, and TRG categories and counted the average accuracy of each category (Figure 3j‐m). Notably, SpecImmune demonstrated relatively lower accuracy for IGH and IGK loci compared to other loci consistently across the four datasets, possibly due to the higher diversity of IGH and IGK loci. With an increase in the sequencing depth, the typing accuracy overall increases, with a more pronounced improvement observed for IGH and IGK loci.

Furthermore, 11 targeted TCR amplicon HiFi sequencing samples validated SpecImmune's reliability for germline TCR typing. The reference allele types of the TRA/D and TRB loci were reported by the previous research [43]. The types reported as novel were discarded to ensure a fair evaluation. Also, the loci with sequencing depth lower than 2x were discarded. There were 3410 alleles of these TRA/D and TRB loci remaining after the discarding. In these alleles, SpecImmune successfully typed 3408 alleles, with an accuracy of 99.9% (Figure S10c, Supporting Information). Notably, SpecImmune achieved a 100% accuracy for all the loci except for *TRAV5* and *TRBV17*. Figure S10d (Supporting Information) shows the number of alleles used to evaluate each locus.

Additionally, SpecImmune infers the haplotype across different gene loci accurately. Leveraging the long reads, SpecImmune can group multiple gene loci into the same phase block. In the 9 HGSVC HiFi samples, the number of heterozygous gene loci of each phase block was counted. A phase block constructed by SpecImmune covers 4.5 heterozygous gene loci on average (Figure S10e, Supporting Information). The largest phase block covers 30 heterozygous gene loci. Further, the accuracy of the phase blocks was assessed by trio consistency. The 9 HGSVC HiFi samples consist of three family trios. For every pair of adjacent heterozygous gene loci within the same phase block in the child, we verify their trio consistency by confirming the presence of both haplotypes in the parents. In these three trios, 84.6% (115/136) of the adjacent heterozygous gene loci pairs are phased in a trio‐consistent manner. We illustrate the haplotypes across 17 gene loci inferred by SpecImmune fit trio consistency in two family trios (Figure 3n; Figure S10f, Supporting Information). Detailed family pedigree information for these trios is provided in Table S2 (Supporting Information). IG/TCR typing results from SpecImmune on benchmark datasets are provided in Table S9 (Supporting Information).

### 2.5 First Method for Multi‐Locus CYP Genotyping using Long‐Read Sequencing

SpecImmune excels in accurately typing *CYP2D6* over current methods, especially in error‐prone CLR and ONT data. Additionally, SpecImmune effectively types other CYP loci where other methods fall short. SpecImmune was compared with pangu (https://github.com/PacificBiosciences/pangu) for *CYP2D6* typing in 32 HPRC HiFi, 25 HPRC ONT, 10 HiFi amplicon, and 56 1kGP ONT samples (Table 2). The *CYP2D6* truth derives from the GeT‐RM project [44] and previous studies [29]. The method pangu is designed for HiFi data; as there is a lack of computational tools to type *CYP2D6* based on other long‐read sequencing protocols, pangu is also utilized as the baseline method in ONT data besides HiFi data. PLASTER is discarded in comparison as it is only designed for the amplicon data [29].

SpecImmune performs slightly better than pangu for *CYP2D6* typing in HiFi data (Figure 3o). In 32 HPRC HiFi samples, the *CYP2D6* typing accuracy of SpecImmune and pangu were 100% (64/64) and 95.3% (61/64), respectively. As read depth varies across samples, we evaluated the impact of sequencing depth on SpecImmune and pangu by applying a series of read‐count cutoffs; at each cutoff, accuracy was computed using only samples whose read counts met or exceeded the specified threshold. The accuracy of SpecImmune is 100% with different read‐count cutoffs. While increasing the number of reads, the accuracy of pangu increases. With no less than 50 reads, the accuracy of pangu also achieves 100% (20/20). We further quantified CNV/fusion concordance, defined as inference accuracy restricted to alleles exhibiting CNV or gene fusions. Both SpecImmune and pangu achieved 100% concordance (7/7) for these CNV/fusion alleles. The result shows that both SpecImmune and pangu are accurate with HiFi data.

Notably, in ONT data, SpecImmune consistently outperformed pangu in the inference of *CYP2D6* star alleles, with SpecImmune's advantage becoming more significant as the read‐count cutoff increased. Among the 32 HPRC ONT samples, the accuracies of SpecImmune and pangu were 68% (34/50) and 64% (32/50), respectively. As the read‐count cutoff increased, SpecImmune demonstrated a continuous improvement in accuracy (as shown in Figure 3p). Specifically, with a minimum of 20 reads, SpecImmune achieved an accuracy of 81.2% (13/16). Conversely, the performance of pangu deteriorated with the increasing read‐count cutoff, as its accuracy dropped to 43.8% (7/16) with at least 20 reads. The CNV/fusion concordance of both SpecImmune and pangu in the HPRC ONT dataset was 25% (1/4). In the 56 ONT samples from the 1kGP, the accuracies of SpecImmune and pangu in inferring *CYP2D6* star alleles were 75.9% (85/112) and 71.4% (80/112) respectively. With the increased read‐count cutoff, SpecImmune's accuracy continued to rise (refer to Figure 3q). Notably, with a minimum of 50 reads, SpecImmune achieved an accuracy of 87.5% (7/8). Conversely, the performance of pangu seemed to suffer from an increase in read‐count cutoff, dropping to 64.3% (18/28) with at least 40 reads, and further decreasing to 62.5% (5/8) with at least 50 reads. For CNV/fusion alleles, SpecImmune achieved higher concordance (42.9%, 3/7) than pangu (28.6%, 2/7). Both ONT datasets consistently illustrate that SpecImmune is a more reliable choice compared to pangu for analyzing ONT data. The increase in the number of reads consistently benefits SpecImmune while negatively impacting pangu's performance. Also, both tools exhibit limited accuracy in detecting CNV/fusion alleles in ONT data.

Additionally, SpecImmune is reliable for *CYP2D6* typing in HiFi amplicon data. We run SpecImmune and pangu on 10 HiFi *CYP2D6* amplicon data. The pangu's results cannot be used in this dataset; it generates over ten haplotypes for each sample. In contrast, SpecImmune achieves an accuracy of 98% (19/20) (Figure 3r). These results showed that SpecImmune is accurate for *CYP2D6* typing in both HiFi and ONT WGS data; it can also handle the amplicon sequencing data.

Furthermore, SpecImmune demonstrates accurate typing capabilities across multiple CYP loci beyond *CYP2D6*. We conducted simulations involving 50 samples with 95% sequencing accuracy and a depth of 100x for all 13 CYP loci. SpecImmune exhibited an overall accuracy of 99.1% (1288 out of 1300) in identifying star alleles across these loci (Figure S10g, Supporting Information). To gauge SpecImmune's resilience against sequencing errors, we simulated 50 samples with 90% sequencing accuracy and maintained a depth of 100x for all 13 CYP loci. In this scenario, SpecImmune achieved an accuracy of 98.9% (1286 out of 1300), slightly below the accuracy observed with 95% sequencing accuracy. These findings underscore SpecImmune's reliability in typing various CYP genes from long reads and its robustness in handling sequencing errors. CYP typing results from SpecImmune on benchmark datasets are provided in Table S10 (Supporting Information).

### 2.6 Heightened Germline IG/TCR Heterozygosity in African Populations

**FIGURE 4:**
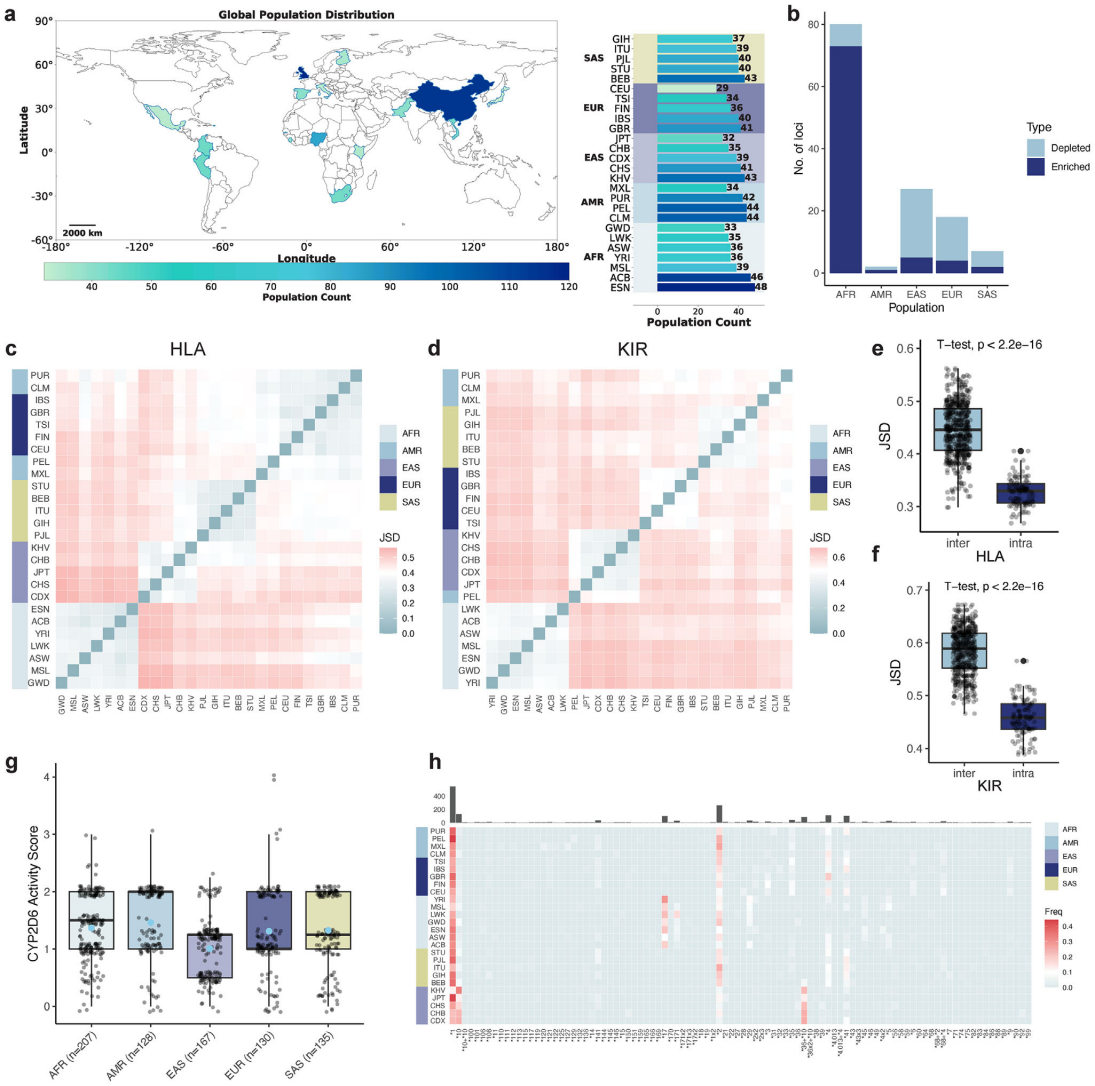
Association between population and immune gene allele distribution in 1kGP. (a) Sample sizes of different populations in the 1kGP dataset. (b) Counts of IG/TCR loci enriched and depleted heterozygous variants (p.adj<0.01) across all super populations. (c) Quantification of HLA allele frequency differences using JSD among different populations. (d) Quantification of KIR allele frequency differences using JSD among different populations. (e) Comparison of JSD of HLA allele frequency distributions within (intra) and between (inter) superpopulations. Boxplots summarize the distribution of JSD values, with individual points representing all pairwise population comparisons. (f) Comparison of JSD of KIR allele frequency distributions within (intra) and between (inter) super populations. Boxplots are overlaid with individual data points to illustrate the full distribution of pairwise population comparisons. (g) Boxplots of *CYP2D6* activity scores across super populations, with individual data points overlaid. (h) Heatmap displaying the frequency of each *CYP2D6* allele in each population. The top bar plot illustrates the count of each *CYP2D6* allele detected across all populations.

Using SpecImmune, we identified a novel observation: African populations exhibit elevated germline heterozygosity in IG and TCR loci. In addition, we confirmed geographic correlations in HLA, KIR, and CYP allele distributions, consistent with prior studies [25, 45, 46]. To systematically assess immune‐related gene diversity, we analyzed 1019 1kGP ONT WGS samples from 26 populations [31], categorized into five superpopulations: 189 from Europe (EUR), 192 from East Asia (EAS), 199 from South Asia (SAS), 275 from Africa (AFR), and 164 from the Americas (AMR) (Figure 4a).

Our analysis revealed that the African population harbors significantly higher germline IG/TCR gene heterozygosity compared to other populations. Specifically, among the 80 loci with significant differences in heterozygous variant counts between African and non‐African populations (p.adj < 0.01), 91.2% were enriched in the African population. Notably, loci *IGHV7‐81* and *TRGV10* exhibited the most pronounced enrichment. In other populations, the proportion of heterozygous variant enrichment was lower than in AFR, with values of 18.5% in EAS, 22.2% in EUR, 28.6% in SAS, and 50% in AMR (Figure 4b; Figure S13, Supporting Information). Furthermore, a multivariate regression analysis that accounted for per‐locus sequencing depth, read length, basecaller model, and mapping quality indicated that AFR populations exhibit a significantly higher number of heterozygous variants compared to non‐African populations (two‐sided t‐test, $p=9.45\times 10^{-86}$, Figure S14, Supporting Information). These results underscore the exceptional genetic diversity of African populations, consistent with their previously established higher overall genetic diversity [30, 31, 33, 47].

As anticipated, HLA, KIR, and CYP allele frequencies demonstrated significant population‐specific patterns. Populations within the same superpopulation displayed similar HLA allele frequencies (p < $2.2\times 10^{-16}$, Figure 4c,e), with KIR alleles showing similar geographic specificity (p < $2.2\times 10^{-16}$, Figure 4d,f). East Asian populations exhibited significantly lower *CYP2D6* activity scores (two‐sided t‐test, p = $3.45\times 10^{-12}$), with haplotypes *10 and *36+*10 being highly prevalent in this group, while haplotype *17 was enriched in African populations (Figure 4g,h). These findings align with prior research linking HLA, KIR, and CYP allele distributions to geographic origins [25, 45, 46].

Additionally, we observed distinct variations in HLA and KIR allele frequency and diversity across loci, while significant heterozygosity level disparities were detected among IG/TCR gene loci (Note S1.2–S1.4 and Figures S15–S22, Supporting Information).

### 2.7 Immune‐Related Genes Suggest Two Distinct Cross‐Family Co‐Evolutionary Communities

Population‐level analysis of immune gene association reveals two major cross‐family gene communities that might have evolved under distinct selective pressures. We analyzed allele frequency correlations across five immune‐related gene families (HLA, KIR, CYP, IG, and TCR) using SpecImmune's genotyping data in the 1,019 1kGP ONT samples from 26 globally distributed populations [31].

**FIGURE 5:**
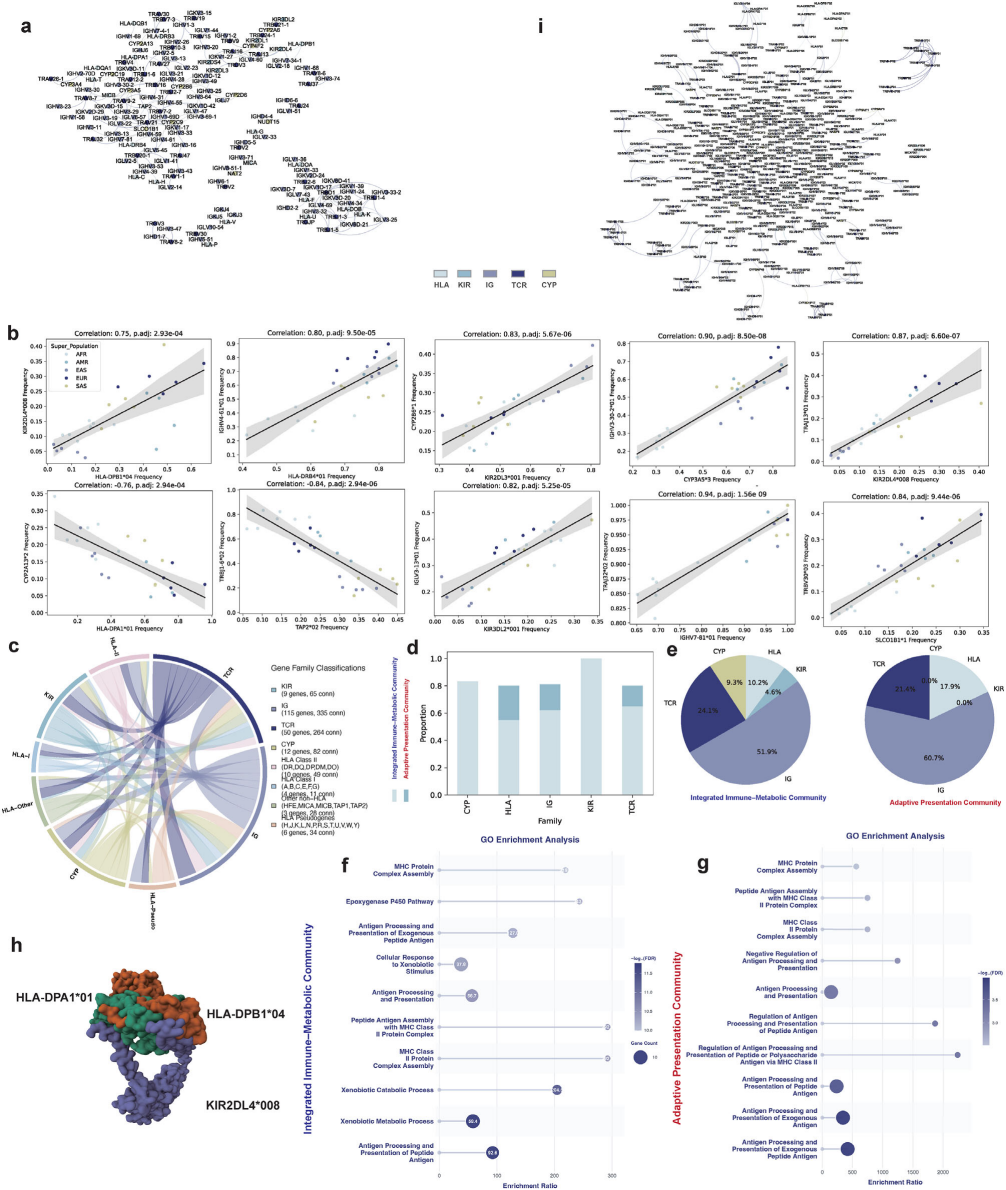
Co‐evolution analyses of immune‐related genes in the 1kGP cohort. (a) Gene association network: nodes represent gene loci, and edges indicate significant associations between genes. Only associations passing entropy filtering, population‐structure adjustment, empirical and permutation testing, and FDR correction were retained. Node colors distinguish different gene families. (b) Most significant allele–allele associations within each gene family pair. in each subplot, dots represent populations; the x‐ and y‐axes show allele frequencies in those populations, and dot colors indicate population identity. (c) Cross‐family correlation between gene subfamilies. The number of associated gene pairs is shown on a log scale. (d) Gene composition within the Integrated Immune–Metabolic Community and the Adaptive Presentation Community. (e) Proportion of each gene family represented in Integrated Immune–Metabolic Community and Adaptive Presentation Community. (f, g) GO enrichment analysis for genes in Integrated Immune–Metabolic Community (f) and Adaptive Presentation Community (g), respectively. (h) The HLA‐DP‐KIR2DL4 complex model predicted by AlphaFold 3 [79] and ClusPro 2 [49]. (i) Allele association network inferred from a precision matrix. Nodes represent alleles, and edges indicate direct associations inferred from the precision matrix. Only edges with values exceeding 0.02 in the precision matrix are retained. Node colors indicate gene families.

For each cross‐family allele pair, we computed Pearson correlations of allele frequencies across populations [48]. Significance was assessed using Benjamini–Hochberg false discovery rate correction, and only associations with adjusted p<0.05 were retained. We controlled for confounding population structure by requiring associations to survive multiple robustness checks, including empirical and permutation testing (p<0.05), PC‐based correction, block bootstrapping, and split‐sample replication as described in the Methods. The final set of significant correlated allele pairs is provided in Table S11 (Supporting Information). In total, 227 cross‐family allele pairs showed confident associations: the most significant correlation between HLA and KIR is the allele HLA‐DPB1*04 and KIR2DL4*008, while the most significant correlation between HLA and CYP is HLA‐DPA1*01 and CYP2A13*2, with correlations between other families also significant. Further, cross‐family correlations were constructed into an association graph where nodes represent gene loci and edge weights denote the highest pairwise correlation (r) between alleles from two loci [48]. The resulting graph comprises 167 nodes (20 HLA, 5 KIR, 12 CYP, 90 IG, and 40 TCR genes) with density 0.0143 (Figure 5a,b). The graph reveals extensive cross‐talk among immune and metabolic gene families (Figure 5c).

The graph resolved into two major connected components comprising 108 and 28 nodes, respectively, along with ten minor components containing fewer than five nodes each. The largest component included classical HLA class I and II loci, KIR loci, IG loci, TCR loci, and CYP loci; we name it as *Integrated Immune–Metabolic Community*. The second‐largest component was enriched for nonclassical HLA loci, TCR loci, IG loci, which is termed as *Adaptive Presentation Community* (Figure 5d,e). Functional Gene Ontology (GO) enrichment analysis showed that the Innate–Metabolic Integration Community uniquely coupled antigen presentation with xenobiotic metabolic and detoxification pathways, whereas the Adaptive Presentation Community was strongly and specifically enriched for MHC class II–mediated processing and presentation of exogenous peptide antigens (Figure 5f,g).

Further structural analysis suggests a potential interaction between HLA class II molecules and KIR loci. Specifically, HLA‐DPB1*04 encoding the beta chains of the HLA‐DP heterodimer–show significant allele‐level correlation with KIR2DL4*008. To examine whether this statistical signal could be consistent with a direct physical interaction, we performed protein–protein docking and evaluated binding strength using multiple independent docking engines to improve robustness. Across five docking platforms (ClusPro [49], ZDOCK [50], HDOCK [51], HawkDock [52], and GeoDock [53]), we consistently obtained high‐affinity complex models for the HLA‐DPB1*04–KIR2DL4*008 pair (Figure 5h), with PRODIGY [54] estimates of ΔG ranging from −15.6 to −17.3 kcal ${\mathrm{mol}}^{-1}$ and predicted $K_{d}$ values on the order of $∼10^{-12}$ M at 25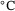 (Table S12, Supporting Information). The inferred interfaces were broadly concordant in their physicochemical composition—featuring substantial charged–apolar and apolar–apolar contacts (e.g., 34–41 and 51–59 interactions, respectively) together with recurrent charged–charged and charged–polar interactions—suggesting a plausible and stable binding mode despite methodological differences among docking algorithms. While prior studies have suggested that HLA class II molecules may modulate NK‐cell activity indirectly [55] (e.g., via NKp44), these results raise the possibility that HLA‐DPB1*04 may engage KIR2DL4*008 more directly, providing a structural hypothesis consistent with the observed genetic association.

Moreover, precision matrix analysis confirms extensive cross‐family direct allele associations. We utilized the precision matrix technique to remove the indirect allele associations. To infer direct allele–allele associations, we constructed a precision‐matrix network from PC‐adjusted allele‐frequency residuals. Edges were initially defined by an absolute precision value $|\Theta_{ij}|>0.02$, a cutoff chosen based on a sharp sparsity transition (Figure S22, Supporting Information). To ensure robustness, we retained only edges that exceeded this threshold in at least 75% of bootstrap resamples of populations, yielding a high‐confidence direct association network (Methods). The precision‐matrix network revealed that many direct associations occur within the same gene family, or even within the same gene, consistent with shared functional and evolutionary constraints (Figure 5i). Importantly, the network also identified a subset of robust cross‐family associations, such as between HLA‐DPB1*13 and CYP2C19*17, providing independent evidence for direct co‐evolutionary interactions across distinct immune‐related gene families.

Overall, our findings reveal association among immune‐related gene families, consistent with, but not conclusive of, a shared evolutionary history. The two major communities identified may represent semi‐independent immune strategies shaped by different selective pressures. Further investigation is needed to elucidate the underlying mechanisms driving these patterns.

### 2.8 Enabling Personalized Drug Guidance Through HLA and CYP Genotyping

Leveraging HLA and CYP typing, SpecImmune enables allele‐based drug recommendations. By integrating PharmGKB's clinical guidelines and FDA drug label annotations [56], it offers allele‐based dosing recommendations tailored to individual genetic profiles. Its comprehensive database, encompassing 154 472 allele annotation entries from the HLA and CYP gene families, links alleles to clinically relevant phenotypes such as drug toxicity and efficacy, supported by evidence scores to ensure reliability and applicability [57].

In precision medicine, accurate genotyping is essential for optimizing drug metabolism and treatment regimens. In the HPRC HiFi dataset, for sample HG00673, SpecImmune correctly identified the *CYP2D6* genotype as *1/*10, while pangu misclassified it as *1/*75. Given the *10 allele's decreased function (activity score 0.25) and the *75 allele's even lower activity, this error could lead to inappropriate dosing adjustments, undermining treatment safety and efficacy. Similarly, for sample HG01071, SpecImmune accurately identified the *CYP2D6* genotype as *4/*41, corresponding to an intermediate or poor metabolizer phenotype, while pangu reported it as *39/*39. Misclassifying *4/*41 (no‐function and decreased‐function alleles) as *39/*39 (normal‐function equivalent) could significantly overestimate metabolic capacity, risking drug toxicity or therapeutic failure for narrow therapeutic index drugs like tricyclic antidepressants or opioids.

## 3 Methods

### 3.1 Allele Database Collection

The allele database serves the purpose of read binning and allele sequence nomenclature within the SpecImmune tool. We obtained the IPD‐IMGT/HLA v3.56.0 database [58] for HLA (https://www.ebi.ac.uk/ipd/imgt/hla/index.html), the IPD‐KIR v2.13.0 database [59] for KIR (https://www.ebi.ac.uk/ipd/kir/), the IMGT/V‐QUEST database [60] (2024‐07‐10) for IG and TCR (https://www.imgt.org/IMGTindex/V‐QUEST.php), and the PharmVar v6.1.2.1 database [61] for CYP (https://www.pharmvar.org/).

### 3.2 SpecImmune Algorithm

#### Reads Binning

To enhance accuracy and reduce the computational complexity in genotyping, we allocate reads to their respective gene loci, building upon our prior HLA‐typing approach, SpecHLA [22]. This methodology assumes that a read exhibits the highest identity to the allele from its originating locus when compared to alleles from other loci. For WGS data, we extract reads from specific regions such as HLA and KIR based on their alignment positions on the hg38 reference genome (Figure 1a,b). Subsequently, these extracted reads are aligned to all alleles in the database. SpecImmune supports BWA MEM [62] and Minimap2 [63] as alignment methods. During each read alignment, the identity between the read and the mapped allele is computed as

(1) $$\begin{array}{c} \text{identity}=1-\frac{\text{No. of mismatch bases}}{\text{alignment length}} \end{array}$$

The highest identity among all alleles of a gene locus is designated as the identity for the locus. Subsequently, a read is allocated to the gene locus with the highest identity (Figure 1b). Those with an identity lower than a specific threshold (default set to 85%) were excluded to mitigate noisy reads that likely originated from unrelated genomic regions.

A long read can span multiple gene loci. SpecImmune can assign a read to multiple gene loci to address this scenario. A criterion assigns a read to multiple loci to ensure precision. The distance between every pair of gene loci on the hg38 reference genome is logged. When a read maps to two gene loci, the mapping intervals on the read for the two loci are denoted as [$s_{1}$, $e_{1}$] and [$s_{2}$, $e_{2}$]. The distance between the alignments of the two loci on the read ($D_{reads}$) can be calculated as:

(2) $$\begin{array}{c} D_{reads}=\max\left(0,s_{2}-e_{1},s_{1}-e_{2}\right) \end{array}$$

Assuming the distance between the two loci on the hg38 reference is denoted as $D_{\text{ref}}$, we postulate that the gene distance on hg38 and in an individual genome should align closely. This assumption serves as a constraint for read assignments. If $|D_{reads}-D_{ref}|<\vartheta$ and $D_{reads}>0$, the read is assigned to these two loci. Here, ϑ defaults to 2000. Otherwise, the read is only assigned to the locus with the highest identity.

#### Best‐Matched Alleles Selection

A pair of alleles is meticulously chosen from the database for each gene locus to optimize the fit with the reads binned to it. If the allele pair exists in an individual's genome, the reads will be mapped to it with greater identity and a higher number of matched DNA bases than other allele pairs. We select the best‐matched allele pair based on this assumption. We select a condensed candidate set of alleles from the database for each locus to decrease computational complexity. Within each locus, reads are aligned to all locus alleles in the database using Minimap2 [63] with default parameters. The alignment depth of each allele is tallied. Subsequently, only the top 200 alleles with the highest depth are chosen to create the condensed candidate allele set. Then, the reads are realigned to the condensed candidate allele set using Minimap2 [63] with the parameter ‐p 0.1 ‐N 100000. This parameter is expressly set to retain all potential alignment records for every read. Three matrices, $M^{R\times L}$, $S^{R\times L}$, and $D^{R\times L}$, are established to house alignment metrics between each read r∈R and each allele a∈L. When a read aligns to an allele, $M^{R\times L}$, $S^{R\times L}$, and $D^{R\times L}$ store the count of matched bases, the count of mismatched bases, and the alignment identity, respectively (Figure 1c).

Subsequently, an appropriate allele pair is determined by maximizing the total count of matched bases and the alignment identity across all reads. For every pair of alleles $a_{i}$ and $a_{j}$, the aggregate counts of matched bases ($\alpha_{ij}$), mismatched bases ($\beta_{ij}$), and alignment identity ($\gamma_{ij}$) from all reads are computed (Figure 1c). This computation assigns each read to the allele with the higher identity. Specifically, if $D_{ra_{i}}>D_{ra_{j}}$, the read r is assigned to allele $a_{i}$; if $D_{ra_{i}}<D_{ra_{j}}$, it is assigned to allele $a_{j}$. In the case of $D_{ra_{i}}=D_{ra_{j}}$, the read r is assigned randomly with an equal probability. Let $\xi_{r}=i\;or\;j$ store which allele the read r is assigned to. Only the alignment between the read and the assigned allele is utilized in determining the values of $\alpha_{ij}$, $\beta_{ij}$, and $\gamma_{ij}$ for the given allele pair. The calculation is as follows:

(3) $$\begin{array}{c} \alpha_{ij}=\sum_{r\in R}\left(M_{ra_{i}}1_{\xi_{r}=i}+M_{ra_{j}}1_{\xi_{r}=j}\right) \end{array}$$

(4) $$\begin{array}{c} \beta_{ij}=\sum_{r\in R}\left(S_{ra_{i}}1_{\xi_{r}=i}+S_{ra_{j}}1_{\xi_{r}=j}\right) \end{array}$$

(5) $$\begin{array}{c} \gamma_{ij}=\alpha_{ij}/\left(\alpha_{ij}+\beta_{ij}\right) \end{array}$$

An empirical approach is utilized to determine the optimal allele pair by effectively balancing the maximization of matched bases and alignment identity. α, β, and γ values are computed for each pair of alleles. The allele pairs are then arranged based on the α values. Those pairs with α values lower than the highest α value by a specified cutoff τ form a candidate set. Within this candidate set, the allele pairs are further sorted according to the γ values. The highest γ value is selected as the best‐matched allele pair (Figure 1c).

The beta distribution is employed to determine the heterozygosity or homozygosity of a gene locus initially. For the selected allele pair ($a_{i}$ and $a_{j}$), we calculate the depth of each allele ($d_{i}$ and $d_{j}$) based on the reads assigned to them. Assuming the lengths of the two alleles are $l_{i}$ and $l_{j}$, $d_{i}$ and $d_{j}$ are computed as:

(6) $$\begin{array}{c} d_{i}=\frac{\sum_{r\in R}\left(M_{ra_{i}}+S_{ra_{i}}\right)\cdot 1_{\xi_{r}=i}}{l_{i}} \end{array}$$

(7) $$\begin{array}{c} d_{j}=\frac{\sum_{r\in R}\left(M_{ra_{j}}+S_{ra_{j}}\right)\cdot 1_{\xi_{r}=j}}{l_{j}} \end{array}$$

The frequencies of the two alleles are estimated using the beta distribution B(α,β). The frequency of allele $a_{i}$ ($\kappa_{i}$) is given by:

(8) $$\begin{array}{c} \kappa_{i}=\frac{\alpha +d_{i}}{\alpha +\beta +d_{i}+d_{j}} \end{array}$$

Similarly, the frequency of allele $a_{j}$ ($\kappa_{j}$) is calculated as:

(9) $$\begin{array}{c} \kappa_{j}=\frac{\alpha +d_{j}}{\alpha +\beta +d_{i}+d_{j}} \end{array}$$

By default, α and β are both set to 1. Subsequently, the minor allele frequency (MAF) is determined as $\min(\kappa_{i},\kappa_{j})$. The locus is considered homozygous if MAF<χ and heterozygous otherwise, where χ defaults to 0.3. The following step utilizes the identified best‐matched alleles as personalized reference alleles.

#### Personalized Haplotypes Reconstruction

We reconstruct personalized haplotypes by leveraging the reads aligned to the best‐matched personalized reference alleles. At first, variants are identified based on the reads alignment for both heterozygous and homozygous gene loci. We use Longshot v1.0.0 [64] to call SNVs and identify SVs through Sniffles v2.2 [65], cuteSV [66], and dysgu [67]. The individual SV callsets are merged and consolidated using Truvari [68] to eliminate redundant calls. Specifically, we executed truvari collapse with –intra to consolidate outputs from the three callers into a single sample representation, and ‐k first to prioritize the attributes of the first caller (order: Sniffles, cuteSV, then dysgu). We also applied –gt het to restrict collapsing based on genotypes, while utilizing Truvari's default matching thresholds. Additionally, SpecImmune supports DeepVariant [69] as an alternative small variant caller. Since the reads are already phased, we only perform variant calling without further phasing for heterozygous gene loci. Allele‐specific reads are aligned separately to the two best‐matched personalized reference alleles to identify variants. For homozygous gene loci, as the heterozygous gene loci might be falsely identified as homozygous, these regions may contain heterozygous variants. Therefore, we first perform variant calling, followed by phasing. In this case, locus‐specific reads are aligned to the single best‐matched personalized reference allele. Subsequently, haplotype phasing of SNVs is conducted using WhatsHap v2.3 [34], while joint phasing of both SNVs and SV breakpoints is performed with Longphase v1.7.3 [35].

Next, we reconstruct personalized linear haplotype sequences based on the identified variants. We first directly generate consensus sequences based on phased SNVs on the personalized reference allele for each haplotype. After that, since the connections between phased SV breakpoints on the same haplotype could be non‐linear (Figure 1e), a *haplotype reconstruction* algorithm is applied to obtain entirely linear haplotypes. We segment the personalized reference allele and link the segments to reconstruct the individual haplotype (Figure 1g). We segment the consensus sequences based on the SV breakpoints for each haplotype and then estimate the copy number of each segment. The resulting segments are denoted as $S=\left\{s_{1},s_{2},\text{…},s_{n}\right\}$, where n represents the number of segments.

SpecImmune estimates the copy numbers of genomic segments, denoted as $\left\{c_{1},c_{2},\text{…},c_{n}\right\}$, using a two‐step approach. First, the mean read alignment depth across all n segments is calculated as:

(10) $$\bar{d}=\frac{1}{n}\sum_{i=1}^{n}d_{i},$$

where $d_{i}$ is the read alignment depth of segment i. Second, the copy number $c_{i}$ of the segment i is estimated as:

(11) $$c_{i}=\left\lfloor \frac{d_{i}}{\bar{d}}\right\rfloor ,$$

where ⌊·⌋ denotes the floor function. The weights of the edges are set to the read counts derived from spanning reads, which quantify the strength of adjacency between segments. SpecImmune uses Minimap2 [63] to align and identify all reads spanning two segments. After constructing the graph, we adopt the *haplotype reconstruction* [36, 37] algorithm to reconstruct the haplotype sequence [36, 37].

SpecImmune iteratively performs haplotype sequence reconstruction; we realign the allele‐specific reads to the haplotype sequences obtained from the *haplotype reconstruction* [36, 37]. Following this, we perform variant calling, phasing, segmentation, and *haplotype reconstruction* [36, 37]. This entire process is iteratively repeated until no variant is identified. Then, the personalized diploid haplotype sequences are reconstructed for each locus. To mitigate noise resulting from low sequencing depth, a window‐sliding method is applied to mask low‐depth regions, inspired by previous work [22]. This method involves sliding a 20 bp window along the gene locus, masking any window with a mean depth below a specified threshold value (defaulting to five) with the character N (Algorithm 1, Supporting Information).

#### Nomenclature

SpecImmune assigns an official designation to each personalized haplotype sequence, corresponding to the best‐matched alleles retrieved from the allele database (Figure 1f). This assignment thoroughly compares the reconstructed sequence and all alleles cataloged in the database, utilizing Blastn v2.15.0 [70]. An empirical approach is utilized to select the best‐matched alleles. For each allele, Blastn reports the number of mismatches (m), the length of gaps (g), and the mapped length (L). The identity (ι) is calculated as:

(12) $$\begin{array}{c} \iota =\frac{m+g*w}{L}, \end{array}$$

where w is a hyperparameter defaulting to 0.4. This parameter is utilized to modulate the significance of gaps in identity calculation. Given that the predominant errors in long reads are insertion/deletion errors [64], we employ this hyperparameter to adjust the weighting of gaps, thereby mitigating the influence of insertion/deletion errors.

We select the best‐matched alleles based on the mapped length and identity to prevent cases where alleles exhibit short local alignments but high identity. The process involves creating a candidate set where alleles with a mapped length lower than the highest mapped length value by a specific cutoff (defaulting to 0.02) are included. This candidate set arranges alleles based on their identity (ι) values. Alleles with an ι lower than the highest ι value by a specific cutoff (defaulting to 4e‐4) are designated as the best‐matched alleles. The nomenclature schemes for HLA, KIR, CYP, and IG/TCR alleles are depicted in Figures S1 and S2 (Supporting Information).

#### Report Visualization

To depict novel variants and demonstrate the confidence level of the reported alleles, we visualize the typing alleles and supporting reads within SpecImmune's output report. We have developed a visualization module based on the GenomeView engine [71]. By default, the visualization report comprises six tracks: sample information, results track, coverage track, variant track, alignment track, and candidate alleles track (Figure 1f). The module incorporates various features to improve the visualization of noisy long‐read sequencing data, including from PacBio and ONT platforms. Notably, it consists of a quick‐consensus mode that masks potential sequencing errors while highlighting probable variants. The module supports output in high‐quality formats such as SVG, PDF, and PNG, facilitating the automated generation of visualizations within analysis pipelines.

#### KIR Annotation

To accommodate gene content variation and gene fusions in the KIR loci, we integrate SKIRT into our analysis pipeline. Using the reconstructed haplotype sequences as input, SKIRT is adopted to annotate gene content variations–including deletions, duplications, and gene fusions [39].

#### IG/TCR Typing

Given the relatively short nature of IG and TCR alleles, binning reads to the gene locus poses a challenge. We have devised a specialized pipeline for IG/TCR typing. Initially, all IG/TCR reads are aligned to the no‐alt hg38 reference using Minimap2 [63], which contains only the main chromosomes. Subsequently, SNVs are called using Longshot v1.0.0 [64], and SVs are called using pbsv (https://github.com/PacificBiosciences/pbsv). The variant files of SNVs and SVs are merged using bcftools (https://samtools.github.io/bcftools/bcftools.html). Following this, all variants undergo phasing using WhatsHap v2.3 [34]. Based on the phased variants, two haplotype sequences are then generated for each gene locus. Regions with low sequencing depth are masked utilizing the method above. We deduce the nomenclature for each haplotype sequence employing the specified method. Furthermore, we provide the phase set at the locus for loci with heterogeneous variants. This conveys the phase information between different gene loci.

#### CYP Typing

Copy number variations (CNVs) and gene fusions are commonly observed at the *CYP2D6* locus, presenting a challenge for our core typing method. To tackle this challenge, we have devised a strategy that integrates the results of pangu with our core method for *CYP2D6* typing (https://github.com/PacificBiosciences/pangu). We have adapted the pangu approach with minor adjustments to effectively type alleles that exhibit CNVs and gene fusions. Both our core typing method and pangu are executed independently. To utilize pangu, reads are aligned to the non‐alt hg38 reference. To enhance typing accuracy and mitigate potential errors and ambiguities in the alignments, we refine the alignments as follows. Initially, alignments with an identity below 0.85 are excluded from further analysis. Additionally, in scenarios where alleles may result from a fusion of *CYP2D6* and *CYP2D7*, reads are assigned to the gene with the higher identity. Given pangu's optimization for PacBio HiFi data, we have enriched it with additional allele markers from various data protocols to cater to diverse data sources. If pangu's results suggest the presence of CNVs or gene fusions, we consider pangu's outputs as the final result. Conversely, if the locus represents a standard diploid gene without such variations, we rely on the results generated by our core typing method. The typing of other CYP loci is exclusively conducted utilizing our core algorithm.

We employ the previous Stargazer [72] method to forecast the phenotype using the star alleles as a foundation. *CYP2D6* haplotypes are translated into the standard unit of enzyme activity, referred to as an activity score. These activity scores play a crucial role in predicting the four metabolizer classes, categorized as follows: poor metabolizers with a score of 0; intermediate metabolizers with a score of 0.5; normal metabolizers ranging from 1 to 2; and ultrarapid metabolizers with a score exceeding2.

#### RNA‐Based HLA Typing

For RNA sequencing data, we align locus‐specific reads to the hg38 reference genome and perform variant calling using Longshot v1.0.0. We then phase the variants using WhatsHap v2.3 [73]. Subsequently, allele‐specific reads are generated by leveraging WhatsHap haplotag assignments. These allele‐specific reads are assembled in a reference‐guided manner using StringTie version 2.2.3 [34]. Finally, we generate diploid haplotype sequences by masking intronic regions with Ns.

### 3.3 Benchmark of SpecImmune

#### Benchmark Data

To validate SpecImmune, we gathered a 1kGP dataset [30, 31], two HPRC datasets, and two HGSVC datasets (Table 2). The 1kGP dataset comprises 1,019 ONT WGS samples. The reference HLA types of 1kGP individuals were acquired from https://ftp.1000genomes.ebi.ac.uk/vol1/ftp/data_collections/HLA_types/ [74]. Furthermore, we obtained a HiFi WGS and an ONT WGS dataset from the HPRC project [32]. Additionally, we collected a HiFi WGS dataset and a PacBio CLR WGS dataset from the HGSVC project [33]. Phased assemblies are accessible for samples from the HPRC and HGSVC projects. Sequencing reads of HLA, KIR, IG, TCR, and CYP loci were extracted by aligning the WGS data to the hg38 reference. For CYP typing validation, we also gathered 10 PacBio HiFi amplicon datasets with reference *CYP2D6* alleles from prior research [29]. The reference *CYP2D6* alleles of HPRC datasets were obtained from previous research, inferred from matched short‐read data (https://pacbio.cn/wp‐content/uploads/poster_harting.pdf). The reference *CYP2D6* alleles in the 1kGP dataset were sourced from the GeT‐RM project [44]. Additionally, we collected RNA sequencing data for HG002 LCL and iPSCs, and HG004 and HG005 LCLs, including PacBio Iso‐seq, MAS‐seq, and ONT direct RNA, from the GIAB project [38]. The detailed command parameters employed for SpecHLA, HLA*LA, and SpecImmune are available in Table S1 (Supporting Information).

Additionally, we employed simulated data to assess the performance of SpecImmune. We randomly chose a pair of alleles for each sample for each locus. Subsequently, we generated long reads for each selected allele sequence using PBSIM3 [42] with the configuration –hmm_model P4C2.model. Finally, the simulated reads were consolidated into a single fastq file for each sample. For KIR simulations, 50 replicates were generated for each parameters combination.

#### Allele Annotation on Phased Genomes

To evaluate SpecImmune, we deduce the reference alleles from the phased assemblies of the HPRC and HGSVC samples. For identifying alleles of the intricate genes on a phased reference genome, we align the assembled genome to the allele database of HLA, KIR, IG, TCR, and CYP loci. We employed BWA‐MEM for the alignment process to achieve precise base‐level alignments, as referenced in prior work [75]. Subsequently, reference alleles were chosen based on these alignments. Reference alleles were meticulously selected to optimize alignment length and identity with the phased assembly. For each gene, we compiled the alignment ratio (η), alignment length, and alignment identity of all alleles within the gene locus. The alignment ratio, calculated as the alignment length divided by the allele's length, excluded alleles with η<0.95. Next, a candidate set was created, encompassing alleles with an alignment length lower than the highest value by a specified cutoff. Within this set, alleles were organized based on their identity values, retaining those below the highest identity value by a designated cutoff. These curated alleles constitute the reference set used to evaluate the accuracy of SpecImmune.

#### Benchmark Metrics

The typing accuracy is defined as the ratio of correctly inferred alleles to all inferred alleles. To account for ambiguities in the ground truth and the inferred gene types when counting correctly inferred alleles, we employ a compatible function F to evaluate typing accuracy, as outlined in prior work [22]. The function F(R,I) yields a value of 1 only if the intersection between sets R and I is not empty. Considering a locus, where the reference gene type tuples are denoted as $R_{1}$ and $R_{2}$, and the inferred gene type tuples are represented by $I_{1}$ and $I_{2}$, the calculation for the number of correctly inferred alleles involves determining:

(13) $$\begin{array}{c} \max\{F\left(R_{1},I_{1}\right)+F\left(R_{2},I_{2}\right),F\left(R_{1},I_{2}\right)+F\left(R_{2},I_{1}\right)\}. \end{array}$$

We employ the identical allele database version throughout the evaluation to maintain nomenclature consistency. Given that the HLA typing method HLA*LA prohibits database version alterations, we align the results from HLA*LA to our IMGT/HLA database version. Furthermore, we harmonize the reference HLA alleles reported by the 1kGP with our IMGT/HLA database version to ensure uniformity. Some KIR alleles in the database contain only CDS. Norman et al. [40] reported such CDS‐only alleles. For consistency assessment in KIR typing, we aligned CDS‐only alleles reported by Norman et al. [40] with full‐length alleles to evaluate concordance.

### 3.4 Quantification of Gene Allele Diversity

Allelic diversity at each gene locus was quantified using entropy‐based diversity metrics, with the *Shannon diversity index* [76] serving as the primary measure. This index, rooted in information theory, captures the uncertainty associated with predicting the allelic identity of a randomly selected gene copy, and is sensitive to both allelic richness and evenness. Formally, the Shannon diversity index H is defined as:

(14) $$H\left(p_{1},p_{2},\text{…},p_{n}\right)=-\sum_{i=1}^{n}p_{i}\cdot \log\left(p_{i}\right),$$

where $p_{i}$ denotes the relative frequency of the i‐th allele among the n alleles observed at the locus. This formulation corresponds to the *maximum likelihood estimator* (MLE) of Shannon entropy, which is widely adopted due to its simplicity and interpretability. However, it has been well‐established that $H_{\mathrm{MLE}}$ exhibits a systematic negative bias under limited sample sizes, particularly when rare alleles are present but not observed. Such biases can lead to underestimation of true diversity, especially in highly polymorphic loci such as HLA genes. To mitigate this issue and obtain more accurate estimates of allelic diversity, we additionally employed *unbiased or nearly unbiased estimators* of Shannon entropy, including the first‐order jackknife correction [77] and the nonparametric estimator [78]. These alternative formulations are specifically designed to account for sample‐size‐dependent biases and the presence of undetected low‐frequency alleles.

### 3.5 Quantify Allele Frequency Difference Between Populations

Utilizing SpecImmune, we derive a set of alleles inferred from all 1kGP samples. For each allele, we calculate its frequency across all 26 populations. Subsequently, we quantify the disparity in allele frequencies between every pair of populations. Considering two populations, let $p_{i}$ represent the i‐th allele in the first population, and $q_{i}$ represent the i‐th allele in the second population. Assuming there are a total of n alleles, we have $\sum_{i=1}^{n}p_{i}=1$ and $\sum_{i=1}^{n}q_{i}=1$. The distinction in allele frequencies between the two populations is assessed using the Jensen–Shannon distance (JSD), which is computed as:

(15) $$\begin{array}{c} JSD=\sqrt{\frac{1}{2}\left(D_{KL}\left(p|m\right)+D_{KL}\left(q|m\right)\right)}, \end{array}$$

where m denotes the pointwise mean of p and q, and $D_{KL}$ signifies the Kullback–Leibler divergence. This calculation uses the distance.jensenshannon function provided in the Python Scipy package.

### 3.6 Differential IG/TCR Loci of Heterozygous Variants

SpecImmune infers the count of heterozygous variants at each IG/TCR locus. Subsequently, we identify the differential IG/TCR loci of heterozygous variants, characterized by significantly divergent counts of heterozygous variants between populations. A vector is allocated to each locus to document the number of heterozygous variants in every sample in a group. We assess the two vectors for each locus using a *t*‐test when comparing two groups. The *t*‐test is achieved by the Python scipy.stats.ttest_ind function. The resulting p‐values are adjusted utilizing the Bonferroni method. This is implemented by the Python statsmodels.stats.multitest.multipletests function. Loci with adjusted p‐values below 0.01 are designated as significant differential loci. Further, we performed multivariate linear regression to assess population differences in IG/TCR heterozygosity while controlling for technical covariates (log‐transformed sequencing depth, average read length, mapping quality, and basecaller mode), then compared residual heterozygosity between AFR and non‐AFR populations using *t*‐tests to determine if biological differences persist after accounting for technical variation.

### 3.7 Correlation Estimation of Cross‐Family Alleles

We assessed cross‐family co‐evolution by computing Pearson correlations between allele‐frequency vectors across 26 worldwide 1000 Genomes populations for all allele pairs drawn from different immune‐related gene families, following population‐correlation frameworks [48]. To exclude uninformative variants, alleles were filtered by mean binary entropy across populations (H≥0.15 bits). To control for population structure, correlations were recomputed after regressing out the top five principal components derived from the global allele‐frequency matrix, and only pairs significant in both raw and PC‐adjusted tests (two‐sided p<0.05) were retained. Robustness was further evaluated using population‐level bootstrap resampling (1000 replicates), permutation testing within each gene‐family pair (10 000 random allele pairings), and an empirical null distribution derived from inter‐chromosomal correlations in the ALFRED database [48]. Reproducibility was assessed via split‐sample replication by randomly partitioning allele counts within each population into two halves across 100 valid splits; replication significance was quantified using an upper‐tail binomial test under a chance replication probability $p_{0}=0.5\alpha$ (with α=0.1), followed by FDR correction. Only associations passing all statistical filters were retained for network construction (Note S1.5, Supporting Information).

Precision‐matrix edges were selected using a stability‐based criterion. Allele‐frequency matrices were first adjusted for population structure by regressing out the top five principal components and then standardized across populations. A precision matrix was estimated using the Ledoit–Wolf shrinkage estimator, and edge robustness was evaluated by stability selection through 100 bootstrap resamples of populations (80% sampled with replacement per iteration). An edge was counted in a resample if its absolute precision value exceeded 0.02, and its stability score was defined as the fraction of resamples in which it was selected. The final network retained only high‐confidence edges with stability score ≥0.75.

### 3.8 Protein Structural Analysis

Protein structural analysis was performed using AlphaFold‐predicted protein structures [79]. To model the HLA‐DP heterodimer used in docking, we generated an allele‐specific HLA‐DP complex composed of the disease/association‐linked HLA‐DPB1*04 β chain together with the most frequent population background HLA‐DPA1*01 α chain. Full‐length allele sequences were obtained from the IPD‐IMGT/HLA database, and extracellular domains were retained for structural modeling (signal peptides and transmembrane/cytoplasmic regions were excluded to match the soluble ectodomains used for docking). The HLA‐DPA1*01/HLA‐DPB1*04 heterodimer structure was then predicted using AlphaFold 3 (multimer mode), and the top‐ranked model was used as the receptor for subsequent docking analyses. Protein‐protein docking predictions were conducted using the ClusPro 2.0 [49], ZDOCK 3.0.2 [50], HDOCK 1.0 [51], HawkDock 2.0 [52], and GeoDock 2.0 [53] online servers to generate conformational sampling and clustering of potential binding modes. Contact‐based prediction of binding affinity in protein‐protein complexes was performed using the PRODIGY [54] online server to estimate binding free energy and dissociation constants.

## 4 Discussion

Several bioinformatics methods have recently been developed to genotype immune‐related genes using long‐read sequencing data. Early efforts primarily relied on in‐house scripts to infer immune gene types from long reads [80, 81, 82]. However, the lack of systematic benchmarking in these studies limits the robustness and generalizability of such approaches. To address these limitations, multiple dedicated software tools have since been introduced. The GenDX NGSengine software enables HLA typing from long‐read data but is a commercial tool and not freely available (https://www.gendx.com/product_line/ngsengine/). HLA*LA achieves long‐read HLA typing by projecting read alignments onto a population‐scale HLA reference graph [28]. While effective, this graph‐based strategy is computationally and memory intensive, particularly for ONT data, where read length and error profiles further increase computational burden [27]. SpecHLA performs HLA typing by binning long reads to their source loci and inferring alleles through variant calling and phasing [22]. However, SpecHLA relies on a single default reference allele per locus for read alignment, which can limit variant discovery and phasing accuracy in highly polymorphic regions. Several tools have also been developed for targeted loci. The pbaa pipeline generates high‐quality HLA and *CYP2D6* sequences but is restricted to PacBio HiFi amplicon data (https://github.com/PacificBiosciences/pbAA). Similarly, the *CYP2D6* typing method pangu exclusively supports PacBio HiFi data (https://github.com/PacificBiosciences/pangu). The PLASTER pipeline enables *CYP2D6* typing from long‐read data but is limited to PacBio SMRT amplicon sequencing and does not generalize to whole‐genome or ONT datasets [29]. Collectively, most existing methods are tailored to specific gene families or sequencing protocols, limiting their applicability for joint analysis across diverse immune‐related loci. In this study, we present a unified framework that integrates read binning, optimal allele selection, and iterative graph‐based haplotype reconstruction for immune gene typing. This framework demonstrates high computational efficiency (Note S1, Supporting Information) and is readily extensible across sequencing technologies and immune‐related gene families.

In HLA genotyping from sequencing data, the conventional method of selecting the best‐matched allele pair from the database based on sequencing reads alignment is a well‐established approach known for its reliability. For instance, OptiType [20] maximizes read alignment to chosen alleles using integer linear programming, while PolySolver [21] integrates aligned read base qualities and observed insert sizes through a Bayesian model to select alleles. HLA‐VBSeq [83] optimizes read alignment and quantities to alleles using variational Bayesian inference. However, existing methods following this strategy are primarily tailored for short‐read sequencing data. Long reads present distinct challenges compared to short reads due to their extended length, higher error rates, and predominantly single‐end. The highly variable length of long reads presents a challenge for existing methods. Despite benefiting from their length, long reads can map to nearly all alleles of a specific gene locus, making it impractical to rely solely on maximizing the number of reads mapped to inferred alleles. Our approach proposes a novel method to maximize the alignment identity between reads and alleles. Given that a read is typically derived from a specific allele, the alignment identity between the read and its source allele should surpass that of other alleles. However, a brief partial alignment between the read and a non‐source allele may demonstrate a higher level of identity than a full‐length alignment between the read and the source allele. To address this challenge, we introduce the concept of matched base numbers. Our strategy involves selecting an allele pair that maximizes alignment identity and matched base numbers for all reads. Benchmark tests have demonstrated the effectiveness and reliability of this strategy for choosing the best‐matched allele pair from long‐read data. The selection of the best‐matched allele pair may be incorrect, and novel alleles may also be present. To address these challenges, we map the reads to the best‐matched allele and apply an iterative graph‐based algorithm to reconstruct haplotypes. Extensive benchmarking has consistently demonstrated the reliability of this framework for genotyping from long‐read data across diverse gene families, including HLA, KIR, IG, TCR, and CYP genes. Notably, the underlying framework is highly versatile and can be readily extended to genotype other polymorphic gene families. To support this, we have incorporated a built‐in module named extend into SpecImmune, which enables users to define custom genes and build personalized allele databases. This flexibility empowers users to apply the pipeline beyond the loci evaluated in this study. Comprehensive documentation is provided to guide users in adapting SpecImmune to their genes of interest (Note S1.6, Supporting Information).

SpecImmune can conduct gene typing swiftly, often completing the process within a few hours. By harnessing long‐read real‐time sequencing, SpecImmune can acquire real‐time data, resulting in prompt immune‐related gene typing outcomes. This real‐time functionality proves particularly beneficial in urgent clinical scenarios such as emergency organ transplant matching. Furthermore, SpecImmune offers reconstructed allele sequences, and visual representations of aligned reads supporting these sequences, facilitating users in evaluating the accuracy of the typing outcomes.

In addition to the HLA genes, several other immune‐related genes within the HLA region contribute significantly to immune regulation. For example, *HFE* encodes a membrane protein similar in structure to HLA class I molecules and plays a key role in regulating iron balance [84]. *MICA* and *MICB* are stress‐induced antigens that serve as ligands for the activating receptor NKG2D, which is expressed in NK and T cells [85]. *TAP1* and *TAP2*, members of the ATP‐binding cassette transporter family, are critical for transporting peptide antigens into the endoplasmic reticulum, where they are loaded onto HLA class I molecules for presentation to CD8+ T cells [86]. Additionally, genotyping pseudogenes improves both typing accuracy and biological insight. Due to high sequence similarity with functional genes (e.g., *HLA‐Y* and *HLA‐A*), pseudogenes can cause read misassignment if excluded, leading to incorrect allele calls. Including them enhances read binning and reduces false positives [87]. Also, pseudogenes provide evolutionary context. As remnants of ancestral genes, they reflect historical duplication and mutation patterns, offering valuable information for population and evolutionary studies [88]. The comprehensive locus coverage underscores the utility of SpecImmune for both clinical applications and immunogenomic research.

SpecImmune is explicitly designed to accommodate heterogeneity in long‐read data characteristics, including variation in read length, sequencing accuracy, and coverage depth, through multiple adaptive components of its workflow. First, read binning is performed using alignment identity rather than read count alone, allowing both short and ultra‐long reads to contribute evidence proportionally to their informative content. This design mitigates biases arising from variable read lengths across platforms, particularly between PacBio CLR, PacBio HiFi, and ONT data. Second, SpecImmune employs platform‐agnostic alignment and variant calling strategies, coupled with an explicit mapping identity filter, to tolerate elevated base‐level error rates while suppressing spurious alignments from off‐target or low‐quality reads. This enables robust performance across a wide range of sequencing accuracies without relying on platform‐specific error models. Third, the iterative haplotype reconstruction framework allows genotyping accuracy to improve progressively with increasing read depth, while still producing reliable results at moderate coverage by leveraging long‐range phasing information intrinsic to long reads. Together, these design choices enable SpecImmune to adapt dynamically to diverse long‐read sequencing conditions, providing consistent and reliable immune gene typing across platforms with distinct read length distributions, error profiles, and depth characteristics.

To facilitate the practical application of SpecImmune across different long‐read sequencing platforms, we conducted a systematic evaluation of typing accuracy across a range of read depths for PacBio CLR, PacBio HiFi, and ONT datasets. By titrating the read depth from low to moderate coverage, we assessed the robustness of our method under varying data conditions. As shown in Figure S11 (Supporting Information), SpecImmune consistently maintained high accuracy across tested loci, even at relatively low coverage levels. Based on these analyses, we propose recommended platform‐specific read‐depth thresholds that achieve reliable performance across all loci. These thresholds, summarized in Table S3 (Supporting Information), may serve as general guidance for researchers employing different long‐read sequencing technologies in immune gene typing.

In addition to read depth, sequencing error rates are a critical factor influencing the reliability of typing. To systematically assess the impact of error rates on typing performance, we simulated HLA datasets with read‐level accuracies ranging from 75% to 99% in 2% increments, with 20 replicates per condition. Remarkably, SpecImmune maintained 98.2% typing accuracy across all simulated error profiles (Figure S12, Supporting Information), highlighting its robustness to base‐level errors in long‐read data. However, we recognize that sequencing errors are not the sole challenge–misaligned or off‐target reads can also introduce noise. To address this, we incorporated a mapping identity filter, excluding reads with alignment identity below 85%. This threshold was empirically determined to strike a balance between retaining informative reads and filtering out potential contaminants or spurious alignments.

Our systematic analysis identified several major factors contributing to genotyping inaccuracies. One primary source of error is read misassignment, which commonly arises when reads are shorter than the full gene length and span homologous regions with high sequence similarity. Our method primarily relies on alignment identity to determine read assignment. In ambiguous cases, a read may align with high identity but over a short region to one locus, while also aligning with lower identity but over a longer region to another locus. These trade‐offs present challenges in confidently determining the read's true origin. We also observed errors in allele‐specific read assignment following the best‐matched allele selection step. In some cases, the selected allele pair differed by only a few variants, making accurate read discrimination difficult. Due to the error‐prone nature of long reads, individual reads were sometimes misassigned to the incorrect allele. This misassignment led to incorrect variant identification, ultimately compromising the accuracy of the haplotype reconstruction step. Another significant challenge lies in the detection of SVs. Although long‐read sequencing provides enhanced capability for SV discovery, current SV callers still struggle with highly polymorphic and structurally complex loci, such as *HLA‐DRB1*. In our analysis, even at high sequencing coverage (e.g., 30–45x), we observed instances where ambiguous read alignments and inconclusive split‐read signals led to unreliable or missed SV calls.

For the HPRC and HGSVC datasets, reference alleles used for evaluation were inferred from phased *de novo* assemblies by alignment to curated allele databases. While this strategy provides a practical and high‐quality benchmark for immune‐gene genotyping, it may be affected by residual assembly or annotation errors, particularly in regions with extreme sequence homology or complex structural variation. In addition, the assignment of closely related alleles can be influenced by database completeness and alignment ambiguity. Nevertheless, phased long‐read assemblies from HPRC and HGSVC currently represent the most comprehensive and reliable resource available for establishing allele‐level reference standards across diverse immune loci, and therefore serve as an appropriate benchmark for assessing SpecImmune performance.

While SpecImmune utilizes sequence identity deviation (<100%) as the primary flag for potential novel alleles, statistical inference alone is insufficient given the inherent error rates in current variant calling and phasing algorithms. False positives can frequently arise from technical artifacts, including PCR chimeras, homopolymer‐associated errors, or allele dropout. Consequently, the Haplotype Visualization module serves not merely as a graphical output, but as a critical manual Quality Control interface. This tool allows researchers to visually inspect read alignment and phasing consistency, providing the necessary granularity to discriminate true biological novelty from sequencing noise. To further standardize the verification process for database submission (e.g., IPD, PharmVar), we propose a tiered evidence framework. At a minimum, candidates must meet Essential Evidence criteria: high bidirectional read depth, unambiguous assignment to a single haplotype, and reproducibility across independent library preparations. Ideally, this is substantiated by Confirmatory Evidence, where orthogonal validation (e.g., Sanger sequencing or NGS cross‐validation) and full‐length gene characterization are employed to ensure precise nomenclature assignment. We also performed a comprehensive benchmark assessing the accuracy of SpecImmune's *de novo* allele sequence reconstruction, the results of which demonstrate robust performance and are detailed in Note S1.7 and Figures S24–S26 (Supporting Information).

By extending co‐evolution analysis to the full repertoires of CYP, IG, and TCR genes, we reveal a highly integrated network of immune‐related loci. This network resolves into two major communities– Integrated Immune–Metabolic Community and Adaptive Presentation–suggesting semi‐independent functional modules shaped by distinct evolutionary pressures. The Integrated Immune–Metabolic Community, enriched for classical HLA, KIR, and CYP genes, is associated with pathways linked to innate immunity and metabolic response. In contrast, the Adaptive Presentation Community, dominated by and nonclassical HLA genes, is specialized for adaptive antigen presentation and T cell activation. Although modular, these communities exhibit selective cross‐family associations–such as TCR–IG and CYP–KIR/HLA linkages–indicating functional crosstalk between adaptive, innate, and metabolic systems. Precision matrix analysis supports direct inter‐family interactions, pointing to shared regulatory or selective constraints. To ensure the robustness of the precision matrix‐derived network, we explicitly assessed the sensitivity of the precision matrix edges to the choice of cutoff. We combined a sparsity‐path analysis with stability selection to validate our parameters. The sparsity path analysis revealed a sharp transition in the number of retained edges around an absolute precision threshold of 0.02, motivating this initial cutoff (Figure S23, Supporting Information). Furthermore, we performed stability selection by bootstrap resampling of populations (100 iterations, sampling 80% of populations per iteration). Edges were retained only if they exceeded the precision threshold in at least 75% of resamples. This analysis confirmed that the reported allele‐level network is robust to subsampling variability and not sensitive to the specific cutoff choice. These findings are currently based solely on the 1kGP cohort and warrant validation in independent populations. Expanding this framework to include non‐immune gene families could further illuminate the broader landscape of immune system regulation. Ultimately, a deeper investigation is needed to uncover the molecular mechanisms driving these co‐evolutionary patterns and their implications for immune diversity and disease.

Furthermore, our study faces two primary limitations. First, our study predominantly focuses on typing individual gene loci, lacking information on phasing across different loci. We only inferred the haplotype among genes for IG/TCR typing. Long reads offer valuable long‐range phasing details, presenting an opportunity to reconstruct haplotypes spanning multiple gene loci. Such haplotypes hold significance for medical research and evolutionary studies. Secondly, our core methodology does not account for gene fusion events because we concentrate independently on each gene locus. Complex genes like *CYP2D6* and KIR loci often feature gene fusion events. To address these variants within *CYP2D6*, we incorporate the third‐party tool pangu. The pangu employs an empirical approach that utilizes specific SNPs or SV breakpoints to infer allele types. pangu was primarily designed for high‐accuracy PacBio HiFi reads, and its variant‐centric workflow is therefore more sensitive to sequencing error profiles. When applied to error‐prone ONT data, this design leads to an increased rate of false‐positive variant calls, which in turn degrades allele typing performance. As read depth increases, the accumulation of such spurious variants further amplifies this effect, explaining the observed decline in pangu accuracy with higher ONT read‐count cutoffs. To reduce the impact of sequencing errors, we integrated pangu into our analysis with several targeted adjustments. Specifically, we removed low‐identity read alignments to limit the contribution of error‐driven mappings. In addition, for cases involving potential *CYP2D6–CYP2D7* fusion alleles, reads were assigned to the gene with the higher alignment identity to reduce ambiguity. Finally, we expanded the allele marker set. To address gene fusions in the KIR loci, we incorporate the method SKIRT to annotate such variants based on haplotype sequences reconstructed by SpecImmune. A method that analyzes the entire gene family region, rather than focusing on individual loci, is essential for resolving these complex variant events effectively.

## Author Contributions

Shuai Wang and Xuedong Wang contributed equally to this work. S.C.L. designed and supervised the study. S.W. and X.D.W. developed the software and wrote the manuscript. M.Y.W. and L.S.W. were involved in the software development. Q.Z. was involved in the drug recommendation procedure development and software testing. All authors contributed to the manuscript revisions.

## Conflicts of Interest

The authors declare no conflict of interest.

## Supporting information


**Supporting File 1**: advs74316‐sup‐0001‐SuppMat.pdf.

**Supplemental Video 2**: advs74316‐sup‐0002‐TableS5‐S12.zip.

## Data Availability

The 1KG‐ONT panel was retrieved from https://ftp.1000genomes.ebi.ac.uk/vol1/ftp/data_collections/1KG_ONT_VIENNA/. Sequencing data for the HPRC and HGSVC projects can be accessed at https://humanpangenome.org/ and https://www.internationalgenome.org/data‐portal/data‐collection/hgsvc3, respectively. Corresponding assemblies are available at https://github.com/human‐pangenomics/HPP_Year1_Assemblies for HPRC and https://www.hgsvc.org/resources for HGSVC. GIAB RNA data can be found at https://ftp‐trace.ncbi.nlm.nih.gov/ReferenceSamples/giab/data_RNAseq/. The software package SpecImmune is freely available at https://github.com/deepomicslab/SpecImmune, and the scripts required to reproduce the results are also included in this repository. In this study, we utilized SpecImmune v0.0.1. Additionally, the original source code of SpecImmune has been deposited at Zenodo (https://doi.org/10.5281/zenodo.17196167).
