# Supplementary material for: A Scalable Framework for Comprehensive Typing of Polymorphic Immune Genes from Long‐Read Data
Source: Adv Sci (Weinh). 2026 Feb 11;13(22):e21531. doi: 10.1002/advs.202521531 (PMC13088316; doi:10.1002/advs.202521531)
Supplement: Supplementary file 1 — Supporting File 1: advs74316‐sup‐0001‐SuppMat.pdf. [file ADVS-13-e21531-s001.pdf]

A Scalable Framework for Comprehensive Typing of Polymorphic Immune Genes from Long-Read Data
-
Supplementary Materials

January 23, 2026

Contents

1 Supplemental Note **3**
- 1.1 Computational resource evaluation of SpecImmune . . . 3
- 1.2 HLA and KIR allele frequency exhibit variation across loci . . . 3
- 1.3 HLA and KIR allele diversity exhibit variation across loci . . . 3
- 1.4 Heterozygosity levels vary significantly across IG/TCR gene loci . . . 4
- 1.5 Correlation estimation of cross-family alleles . . . 4
- 1.6 Extending SpecImmune to Additional Gene Loci . . . 5
- 1.7 SpecImmune outperforms a typing baseline and de novo assemblers in novel allele reconstruction 6
- 1.8 SpecImmune exhibits exceptional resilience to transcriptomic fragmentation across long-read sequencing platforms . . . 6

List of Algorithms

1 **Iterative Haplotype Reconstruction** . . . 8

List of Figures

S1 Nomenclature description of HLA/KIR/CYP alleles. . . . 9
S2 Nomenclature description of IG (A) and TCR (B) alleles. . . . 10
S3 Summary of SpecImmune typing results for HG00377 in 1kGP. . . . 11
S4 Computational resource evaluation for HLA typing in HPRC HiFi samples. . . . 12
S5 Computational resource evaluation of SpecImmune for KIR, IG/TCR, and CYP typing on HPRC HiFi samples. . . . 12
S6 Performance of HLA*LA, SpecHLA, and SpecImmune on HGSVC CLR dataset. . . . 13
S7 Performance of HLA*LA, SpecHLA, and SpecImmune on HGSVC HiFi dataset. . . . 14
S8 Performance of HLA*LA, SpecHLA, and SpecImmune on HPRC HiFi dataset. . . . 15
S9 Performance of HLA*LA, SpecHLA, and SpecImmune on HPRC ONT dataset. . . . 16
S10 Evaluation of SpecImmune for typing KIR, IG/TCR, CYP genes. . . . 17
S11 Performance of SpecImmune across sequencing platforms and depths. . . . 18
S12 SpecImmune performance for the HLA genes across sequencing reads accuracy. . . . 18
S13 Landscape of immune-related gene alleles in 1kGP population. . . . 19
S14 Population differences in IG/TCR heterozygosity after controlling for technical covariates. . . . 20
S15 Frequencies of common, low-frequency, and rare alleles at each HLA and KIR locus. . . . 20
S16 Allelic diversity across HLA loci based on Shannon's original entropy formula. . . . 21
S17 Allelic diversity across HLA loci estimated using the unbiased Chao entropy estimator. . . . 21
S18 Allelic diversity across HLA loci estimated using the jackknife Shannon entropy estimator. . . . 22
S19 Allelic diversity across KIR loci based on Shannon's original entropy formula. . . . 22
S20 Allelic diversity across KIR loci estimated using the unbiased Chao entropy estimator. . . . 23

S21 Allelic diversity across KIR loci estimated using the jackknife Shannon entropy estimator. . . . 23
S22 Heterozygous frequencies at IG and TCR loci. 24
S23 Sparsity and stability of precision-matrix–derived networks. 24
S24 Performance comparison of HLA assembly tools on novel alleles from IMGT/HLA-3.62.0. . . . 25
S25 Comparison of assembled sequences for the *HLA-DRB1*08:01:01:05* allele across different software tools. 25
S26 Comparison of assembled sequences for the *HLA-DRB1*11:01:01:22* allele across different software tools. 26
S27 Robustness of SpecImmune to transcriptomic fragmentation across long-read platforms. . . . 26
S28 Evaluation of SpecImmune for full typing HLA genes. 27

List of Tables

S1 Commands used for SpecHLA, HLA*LA, and SpecImmune in evaluation. 28
S2 Trio information in 1kGP 28
S3 Recommended Read-Depth Thresholds for Specimmune 28
S4 HLA Genotyping Accuracies in 1kGP 29

1 Supplemental Note

1.1 Computational resource evaluation of SpecImmune

SpecImmune represents a swift method for immune-related gene typing. In assessing the computational resource utilization of SpecImmune, we undertook HLA, KIR, IG+TCR, and CYP typing individually utilizing the HPRC HiFi dataset, which encompassed 50 PacBio HiFi WGS samples. Reads from each gene family were isolated, and SpecImmune was independently executed for each gene family. Subsequently, we documented the wall-clock time, CPU time, and peak memory usage for each execution. Initially, we juxtaposed the computational resource utilization of SpecImmune against SpecHLA and HLA*LA for HLA typing. We performed the three methods with default parameters using 20 threads. Given the variance in the number of supported gene loci among these three HLA typing methods, we normalized the wall-clock time and CPU time concerning the number of supported gene loci. Notably, the normalized wall-clock time was found to be the shortest for SpecImmune (Figure S4). The normalized CPU time and peak memory usage exhibited similarities between SpecImmune and SpecHLA. In contrast, HLA*LA exhibited significantly higher peak memory consumption and normalized CPU time than the other two methods. On average, SpecImmune required around 10.85G of Peak RAM, 2.22 hours of CPU time, and 0.45 hours of wall-clock time per sample.

In KIR typing, the average CPU time and peak memory usage are 8.78 hours and 0.94 GB, respectively (Figure S5). SpecImmune facilitates simultaneous IG and TCR typing. For IG+TCR typing, SpecImmune exhibits an average CPU time of 8.72 hours and a peak memory usage of 7.33 GB (Figure S5). In CYP loci, the average CPU time and peak memory usage are 0.49 hours and 11.85 GB, respectively (Figure S5). SpecImmune demonstrates efficient memory and time management in typing immune-related genes, making it suitable for execution on standard personal computers. This capability enables SpecImmune to provide real-time gene typing results in tandem with contemporary sequencing technologies, enhancing its utility for clinical applications.

1.2 HLA and KIR allele frequency exhibit variation across loci

Common, low-frequency, and rare HLA allele frequencies exhibit variation across gene loci. We categorized the alleles based on their frequencies within the population (Methods); 9.4% (187), 23.1% (459), and 67.6% (1345) of these identified alleles were classified as common, low-frequency, and rare, respectively (Figure S15). We then analyzed the frequency distribution of these three groups within each gene locus. Notably, none of the *HLA-B* and *HLA-DRB1* alleles were classified as common, while all *HLA-DPB2*, *HLA-V*, *HLA-S*, and *HLA-Y* alleles fell into this category. Among the genes, *MICB* exhibited the highest rare allele frequency at 87.2%, followed by *HLA-DPB1* at 84.8% and *HLA-A* at 83.6%.

Furthermore, the occurrence of rare alleles varies significantly among the KIR loci. We examined the frequency distribution of common, low-frequency, and rare alleles for each locus (Figure S15). Drawing from prior research [1], we delineated alleles into distinct categories based on their occurrence frequencies: common alleles ($> 5\%$), low-frequency alleles (between 0.5% and 5%), and rare alleles ($< 0.5\%$). At each locus, the allele frequency is determined by dividing the number of a specific category by the total number of alleles identified within the population. *KIR2DL2* and *KIR2DL5B* exhibit no rare alleles, with relatively low frequencies of rare alleles observed in *KIR2DL3*, *KIR2DS1*, and *KIR2DS4*. In contrast, the majority of alleles in *KIR2DL1* and *KIR3DL1* are classified as rare.

1.3 HLA and KIR allele diversity exhibit variation across loci

The gene diversity within each of the 26 populations was quantified using three different methods (see Methods). Notably, there was significant variation in diversity across the HLA loci (Figure S16-S18). All three methods consistently identified *HLA-DRB1* as displaying the highest mean diversity among the populations, followed by *HLA-DPB1*, *HLA-A*, and *HLA-B*. For the fifth and sixth positions, the Shannon Diversity Index placed *HLA-C* followed by *HLA-DQB1*, while the other two methods reversed this order, ranking *HLA-DQB1* ahead of *HLA-C*. The notable diversity of these genes is consistent with previous findings in the Han Chinese population [1]. The hierarchy of diversity among *HLA-B*, *HLA-A*, and *HLA-C* conforms to previous research, suggesting that *HLA-B* is the oldest and most diverse locus, while *HLA-C* has evolved more recently [2]. Conversely, *HLA-Y* had the lowest mean diversity, with *HLA-U*, *HFE*, and *HLA-L* following suit. Furthermore, there were no significant differences observed in gene diversity between populations.

The Shannon diversity exhibits significant variation across the KIR loci. We quantified the allelic diversity as distinct from gene content polymorphism at each KIR (Methods). All three methods consistently identified

KIR2DL1 as possessing the highest allelic diversity, followed by *KIR3DP1* (Figure S19-S21). Conversely, *KIR2DL2* demonstrated the lowest allelic diversity among all loci, with *KIR2DS4* exhibiting the second lowest diversity values. The concordance across multiple diversity assessment methodologies substantiates the robustness of these observed patterns of differential allelic diversity across the KIR gene family.

In certain loci, a small number of alleles exhibited notably high frequencies. For instance, the allele KIR3DS1*0130101 (23.2%) emerged as the most frequent among all KIR alleles, closely trailed by KIR2DS1*0020103 (22.6%).

1.4 Heterozygosity levels vary significantly across IG/TCR gene loci

The heterozygosity levels vary significantly across IG/TCR gene loci. A locus was considered heterozygous if two different alleles were present and homologous otherwise within a sample. We calculated the heterozygous frequencies for each locus across all samples (Figure S22a-b). Interestingly, 57.4% (220 out of 402) of the loci exhibit a 0% heterozygous frequency. On the other hand, *IGHV1-69* exhibited the highest heterozygous frequency at 62.4%, followed by *IGHV3-48* at 58.4%, and *TRAV12-2* at 56.8%.

1.5 Correlation estimation of cross-family alleles

Cross-family allele frequency correlations. To investigate population-level co-evolution between immune-related gene families, we analyzed correlations between allele frequencies measured across populations, following the general strategy of Single *et al.* [3]. For each gene family (HLA, KIR, IG, TCR, CYP), we estimated allele frequencies in each of 26 globally distributed 1000 Genomes populations, and computed Pearson correlation coefficients for all cross-family allele pairs. Raw Pearson p-values were adjusted using the Benjamini–Hochberg FDR procedure applied separately within each cross-family comparison.

Entropy-based filtering of uninformative alleles. Alleles that are nearly fixed or uniformly rare across populations provide little information for population-level association tests and can inflate unstable correlations. We therefore filtered alleles based on their mean binary entropy across populations. For an allele with frequency f_p in population p, we computed the binary entropy $h(f_p) = -f_p \log_2 f_p - (1 - f_p)\log_2(1 - f_p)$ (with $h(0) = h(1) = 0$), and defined the allele informativeness as $H = \frac{1}{P}\sum_{p=1}^{P} h(f_p)$, where P is the number of populations. Alleles with $H < 0.15$ bits were excluded prior to all association testing.

Principal component adjustment for population structure. To reduce confounding due to shared demographic history and population stratification, we computed principal components (PCs) from the matrix of allele frequencies across all retained alleles (populations as observations). For each allele pair, we regressed the top five PCs out of both allele-frequency vectors and computed Pearson correlation on the residuals. Associations were retained only if they were significant in both the raw Pearson test and the PC-adjusted residual test (two-sided $p < 0.05$ for each), ensuring that correlations were not solely driven by genome-wide population structure.

Bootstrap stability across populations. To assess robustness to population sampling variability, we performed bootstrap resampling at the population level ($n = 1000$ iterations), sampling populations with replacement and recomputing the correlation each time (using PC-adjusted residuals when applicable). We summarized each association by a 95% bootstrap confidence interval and a sign stability score (fraction of bootstrap replicates preserving the observed correlation direction). Associations exhibiting unstable directionality under bootstrap resampling were excluded.

Permutation testing within gene-family pairs. As an orthogonal null model that preserves family-specific allele frequency distributions, we performed permutation testing by randomly pairing alleles drawn from the two gene families under comparison (n = 10,000 random pairs). The permutation p-value was defined as the fraction of permuted pairs whose absolute correlation exceeded that of the observed allele pair.

Empirical null distribution from unlinked loci. To further control for correlation induced by shared population history, we used an empirical background distribution derived from the ALFRED dataset [3], consisting of correlations among randomly selected polymorphic sites from unlinked genes on different chromosomes profiled across the same populations. We used 10,000 such inter-chromosomal pairs to form an

empirical null distribution of absolute correlations. The empirical p-value for an observed allele pair was computed as the fraction of null pairs with absolute correlation exceeding the observed value.

Split-sample replication and replication-rate significance. We evaluated reproducibility using an internal split-sample replication procedure that partitions allele counts within each population into two equal subsets (A and B) via binomial sampling, recomputes allele frequencies, and tests the association independently in each subset. In each split, an association was considered *discovered* in subset A if the two-sided Pearson test satisfied $p < \alpha$ (with $\alpha = 0.1$). Discovery edges were then evaluated in subset B and were declared *replicated* if (i) the correlation sign matched subset A and (ii) the two-sided p-value satisfied $p < 2\alpha$ (equivalent to one-sided $p < \alpha$ under the direction constraint). This procedure was repeated for 100 valid splits per allele pair (excluding splits in which either allele had zero frequency in any population).

For each allele pair with n discoveries in subset A and x successful replications in subset B, we computed the replication rate $r = x/n$ and assessed its significance using an upper-tail binomial test. Under the null, a replicate occurs by chance when the sign matches (probability 0.5) and the one-sided test is nominally significant (probability α), yielding $p_0 = 0.5\alpha$. We therefore report a replication-rate p-value as $P(X \geq x \mid n, p_0)$, and applied Benjamini–Hochberg FDR correction to replication-rate p-values across tested pairs.

Summary of filtering. Allele pairs were retained if they satisfied all of the following criteria: (1) FDR-adjusted Pearson $p < 0.05$, (2) PC-adjusted residual test $p < 0.05$, (3) empirical null $p < 0.05$, (4) permutation $p < 0.05$, and (5) split-sample replication-rate FDR < 0.05 (binomial test described above).

1.6 Extending SpecImmune to Additional Gene Loci

SpecImmune is designed to be easily extensible to additional gene loci beyond the built-in immune gene families. To facilitate user-defined extensions, SpecImmune provides a dedicated gene family label, `extend`, which allows custom gene sets to be incorporated with minimal modification to the pipeline. Below, we demonstrate how to extend SpecImmune to support non-human KIR (NHKIR) genes as an example.

Step 1: Prepare a Custom Database First, download the reference sequences for the target gene set. For NHKIR genes, the reference FASTA file can be obtained from the IPDNHKIR repository:

```
wget https://raw.githubusercontent.com/ANHIG/IPDNHKIR/refs/heads/Latest/NHKIR_gen.fasta
```

Next, build the SpecImmune database using the provided `make_db.py` script. All extended genes must be assigned to the gene family `extend`:

```
python make_db.py -i extend \
  --extend_fa /path/to/NHKIR_gen.fasta \
  -o /path/to/db
```

Step 2: Update the Gene List To specify which genes should be processed during annotation, edit the gene list in `annoExtend.pl`. For example, to include only the gene `Mafa-KIR3DL20`, update the following block:

```
my @genes = (
    "Mafa-KIR3DL20"
);
```

Additional genes can be included by appending them to this list.

Step 3: Run the Pipeline Prepare the input read file (FASTQ format) and execute the SpecImmune pipeline using the `extend` family:

```
python main.py -i extend --db /path/to/db \
  -r /path/to/test_reads.fq.gz \
  -n test \
  -o /path/to/extend_test
```

Step 4: Inspect the Output The output format is consistent with other SpecImmune analyses and includes inferred genotypes, read support, and matching information. An example result is shown below:

```
# version: N/A
Locus           Chromosome  Genotype
Mafa-KIR3DL20   1           Mafa-KIR3DL20*032:01:01
Mafa-KIR3DL20   2           Mafa-KIR3DL20*002:01:01
```

This example illustrates that SpecImmune can successfully infer extended gene alleles using the same workflow as for built-in gene families.

1.7 SpecImmune outperforms a typing baseline and de novo assemblers in novel allele reconstruction

To evaluate the capacity of SpecImmune for novel allele reconstruction, we benchmarked it against SpecHLA[4] and three widely used *de novo* assemblers (Canu[5], Flye[6], and Hifiasm[7]). The evaluation utilized 700 novel allele sequences introduced in IMGT/HLA release 3.62.0-alpha [8] as the ground truth. We simulated PacBio HiFi reads from these strictly validated sequences across a gradient of sequencing depths (4×, 6×, 8×, 10×, 20×, 30×, 40×, and 50×) and executed all tools using default parameters. Notably, HLA*LA[9] was excluded from this analysis as it is designed exclusively for allele typing and does not support the full sequence reconstruction required for characterizing novel alleles. Performance was assessed based on assembly accuracy (identity, mismatch rate, gap rate) and completeness/contiguity (coverage, total length, number of contigs), using paired t-tests with Benjamini–Hochberg FDR correction.

Relative to the typing baseline SpecHLA, SpecImmune provided consistently higher-fidelity reconstructions across all depths. At low coverage (4×), SpecImmune increased identity by 14.61 percentage points (95% CI 14.08–15.15; $q = 8.01 \times 10^{-249}$), with corresponding significant reductions in mismatch rate (−0.146; $q = 8.01 \times 10^{-249}$) and gap rate (−0.0504; $q = 2.37 \times 10^{-258}$). Although the magnitude of improvement diminished with increasing depth, the advantage remained statistically supported even at 50× (identity: +2.09 percentage points, 95% CI 1.91–2.26; $q = 1.33 \times 10^{-96}$; mismatch rate: −0.0209; gap rate: −0.00805). Regarding completeness, SpecImmune reached 100% coverage at mid-to-high depths, consistently exceeding SpecHLA (e.g., 50×: +3.26 percentage points; $q = 6.42 \times 10^{-148}$) and producing longer assemblies closer to the expected target length (50×: +157.82 bp; $q = 1.50 \times 10^{-111}$). Both SpecImmune and SpecHLA produced single-contig assemblies, yielding no difference in contig count (Figure S24).

Moreover, when compared with *de novo* assemblers, SpecImmune demonstrated substantially larger and highly consistent gains in both accuracy and completeness. Many BH-adjusted q values approached zero due to numerical underflow, reflecting the vast performance gap. At 4×, SpecImmune increased identity versus Canu, Flye, and Hifiasm by +75.71, +70.31, and +60.61 percentage points, respectively, and improved coverage by approximately 60 percentage points (all $q \approx 0$). This dominance persisted at 50×, where SpecImmune continued to outperform Canu (+12.01 pp identity), Flye (+8.97 pp identity), and Hifiasm (+8.23 pp identity).

Crucially, SpecImmune yielded superior contiguity, consistently producing a single contig across depths. Conversely, the *de novo* methods were significantly more fragmented (e.g., at 4×: ∼3 fewer contigs for SpecImmune; all $q < 10^{-250}$). An inherent limitation of these *de novo* assemblers is that they are unphased, often generating multiple fragmented primary sequences rather than a single coherent haplotype. Furthermore, the resulting assemblies are frequently incomplete. This lack of continuity and phasing integrity significantly compromises downstream HLA typing accuracy, as illustrated in Figure S25 and Figure S26. Collectively, these results indicate that SpecImmune provides statistically supported improvements over a dedicated typing approach and delivers substantial advantages over general-purpose *de novo* assembly tools across a wide range of sequencing depths.

1.8 SpecImmune exhibits exceptional resilience to transcriptomic fragmentation across long-read sequencing platforms

To rigorously assess the susceptibility of SpecImmune to RNA degradation—specifically the 5' truncation artifacts characteristic of long-read RNA-seq workflows—we executed a systematic simulation study utilizing ground truth HLA alleles derived from verified coding sequences within the IMGT/HLA database. For each iteration, reference alleles were randomly selected to ensure unbiased representation of HLA diversity. Independent replicates ($n = 500$) were synthesized using the PBSIM simulator with a default sequencing

depth of 50x across three distinct sequencing modalities (PacBio HiFi, CLR, and ONT), subjecting them to a gradient of degradation coefficients ranging from mild (0.1) to severe (0.5).

Our quantitative analysis demonstrates that transcript degradation exerts a negligible influence on the predictive fidelity of SpecImmune, underscoring the algorithm's intrinsic robustness (Figure S28). Notably, the PacBio HiFi platform demonstrated superior stability, maintaining an accuracy of 99.20% at mild degradation (0.1) and sustaining 98.85% even under severe conditions (0.5), representing a minimal decline ($\Delta = -0.35\%$). Furthermore, even on platforms characterized by higher stochastic error profiles, the impact of degradation was effectively mitigated. Under the most stringent degradation conditions, the PacBio CLR platform exhibited a reduction of only 1.25% (declining from 98.35% to 97.10%), while the ONT platform demonstrated a similarly constrained decrease of 0.80% (from 98.65% to 97.85%).

Across all experimental conditions, SpecImmune achieved a global average accuracy of 98.34%, with Class I and Class II genes averaging 98.65% and 98.36%, respectively. The consistently narrow confidence intervals (mean width = 1.106%, max = 1.481%) observed across the 500 replicates further corroborate that SpecImmune preserves high-precision HLA typing capabilities despite significant compromises in transcript integrity.

Algorithm 1 Iterative Haplotype Reconstruction

Input:
Aligned reads R, personalized reference alleles P, window size for masking low-depth regions w (default = 20 bp), depth threshold τ (default = 5).

Output:
Reconstructed personalized diploid haplotype sequences $H = \{H_1, H_2\}$.

Initialize haplotypes: $H \leftarrow \emptyset$
Initialize variant identification flag: variants_identified $\leftarrow$ True
while variants_identified = True **do**
  (a) Realign reads:
  $R' \leftarrow$ Realign(R, H or P)
  (b) Variant calling:
  SNVs $\leftarrow$ CallSNVs(R')
  SV_breakpoints $\leftarrow$ CallSVBreakpoints(R')
  (c) Variant phasing:
  Phased_SNVs $\leftarrow$ PhaseSNVs(SNVs, R')
  Phased_SNVs_and_SVs $\leftarrow$ JointPhase(Phased_SNVs, SV_breakpoints)
  (d) Consensus sequence reconstruction:
  Consensus $\leftarrow$ GenerateConsensus(Phased_SNVs_and_SVs, P)
  (e) Segmentation:
  $S = \{s_1, s_2, \ldots, s_n\} \leftarrow$ SegmentConsensus(Consensus, SV_breakpoints)
  (f) Copy number estimation:
  for $s_i \in S$ **do**
    $c_i \leftarrow \left\lfloor \frac{d_i}{\frac{1}{n}\sum_{j=1}^{n} d_j} \right\rfloor$
  end for
  (g) Conjugate graph construction and haplotype reconstruction:
  Construct a conjugate graph $G(S, E)$ with edge weights:

$$w(s_j, s_i) = \Theta(s_j, s_i) \quad \text{(spanning read count between } s_j \text{ and } s_i\text{)}$$

  $H \leftarrow$ PerformHaplotypeReconstruction(G)
  (h) Mask low-depth regions:
  $H \leftarrow$ MaskLowDepthRegions(H, R', w, τ)
  (i) Variant identification:
  New_Variants $\leftarrow$ IdentifyNewVariants(H, R')
  variants_identified $\leftarrow$ (New_Variants $\neq \emptyset$)
end while
Return: $H = \{H_1, H_2\}$

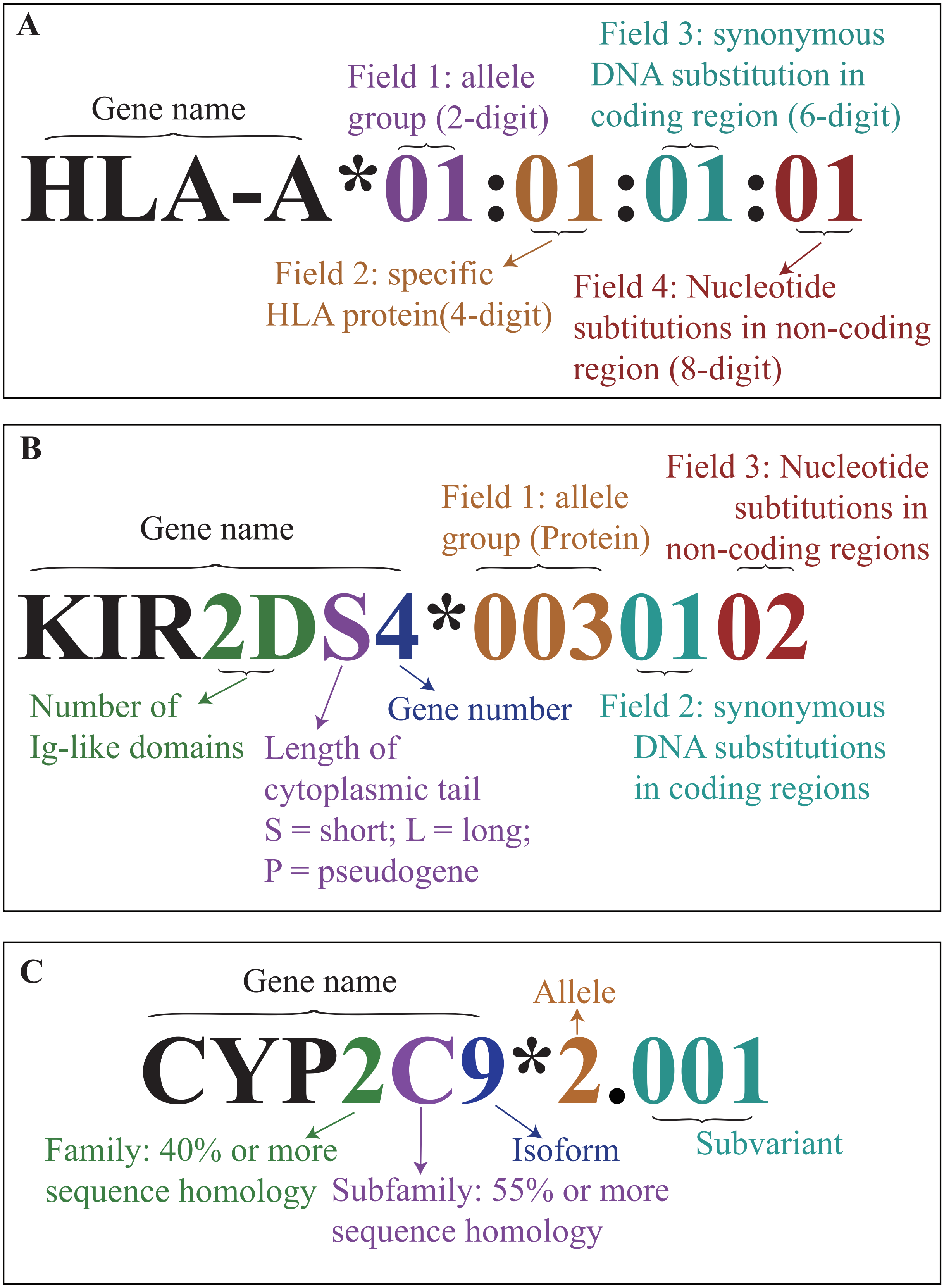

Supplementary Figure S1: Nomenclature description of HLA/KIR/CYP alleles. (A) HLA, (B) KIR, and (C) CYP nomenclature with different allele resolutions [10, 11, 12, 8].

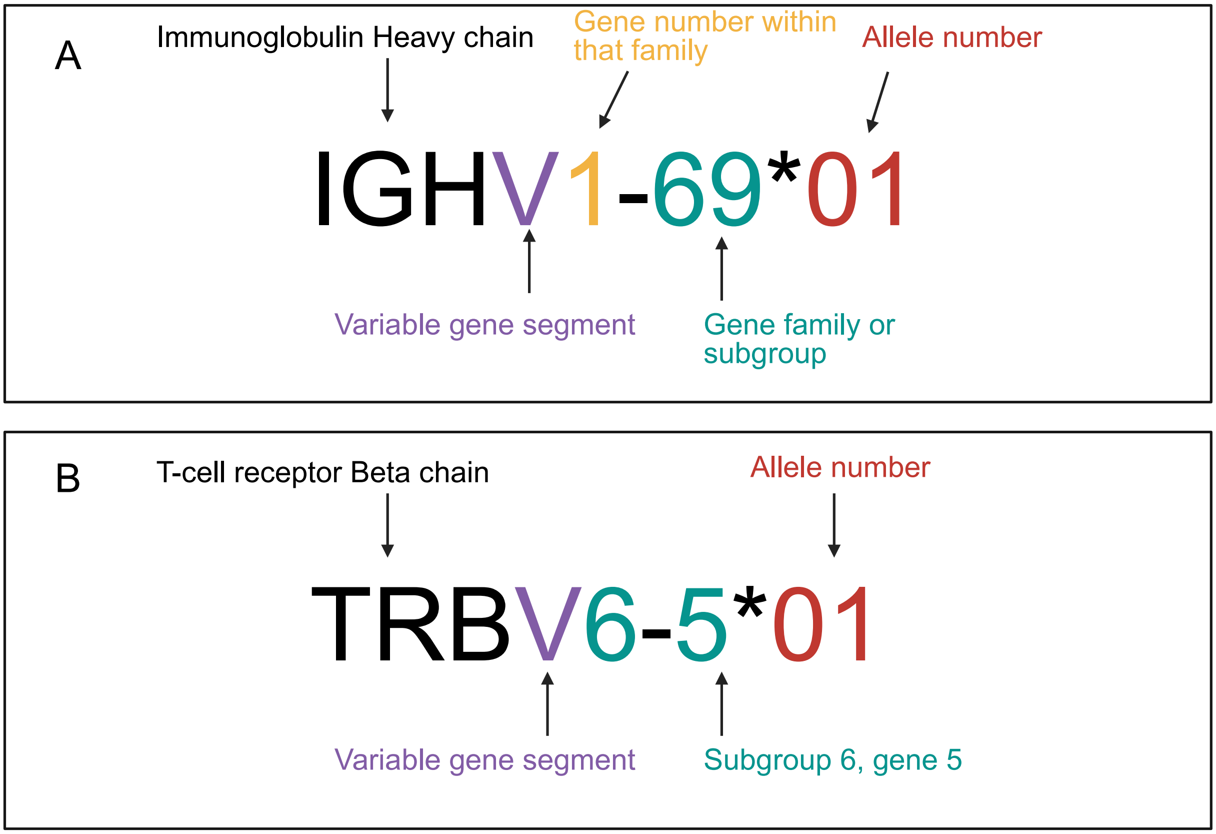

Supplementary Figure S2: Nomenclature description of IG (A) and TCR (B) alleles. Refer to [8].

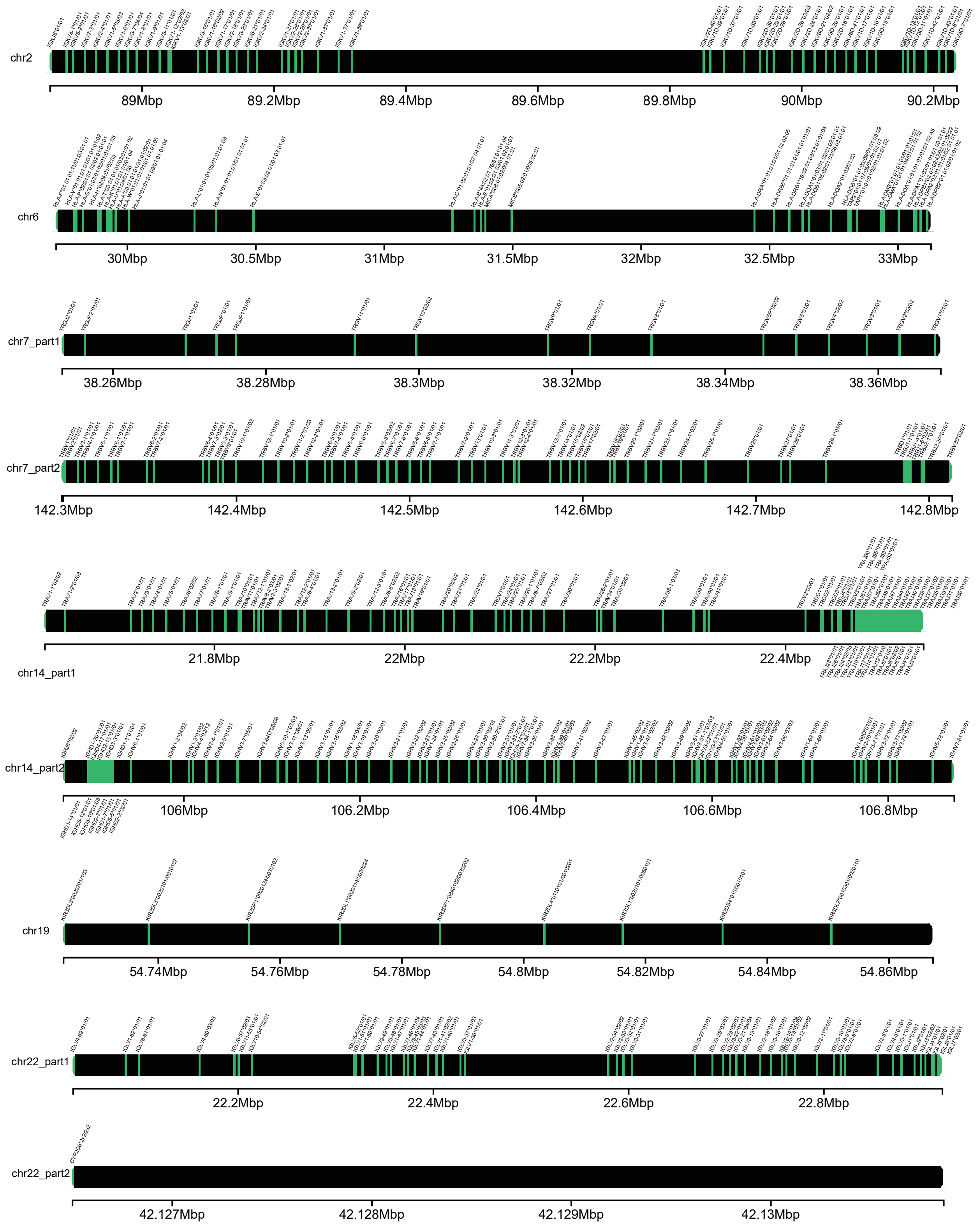

Supplementary Figure S3: Summary of SpecImmune typing results for HG00377 in 1kGP. Visualization includes only loci on the main chromosome of hg38.

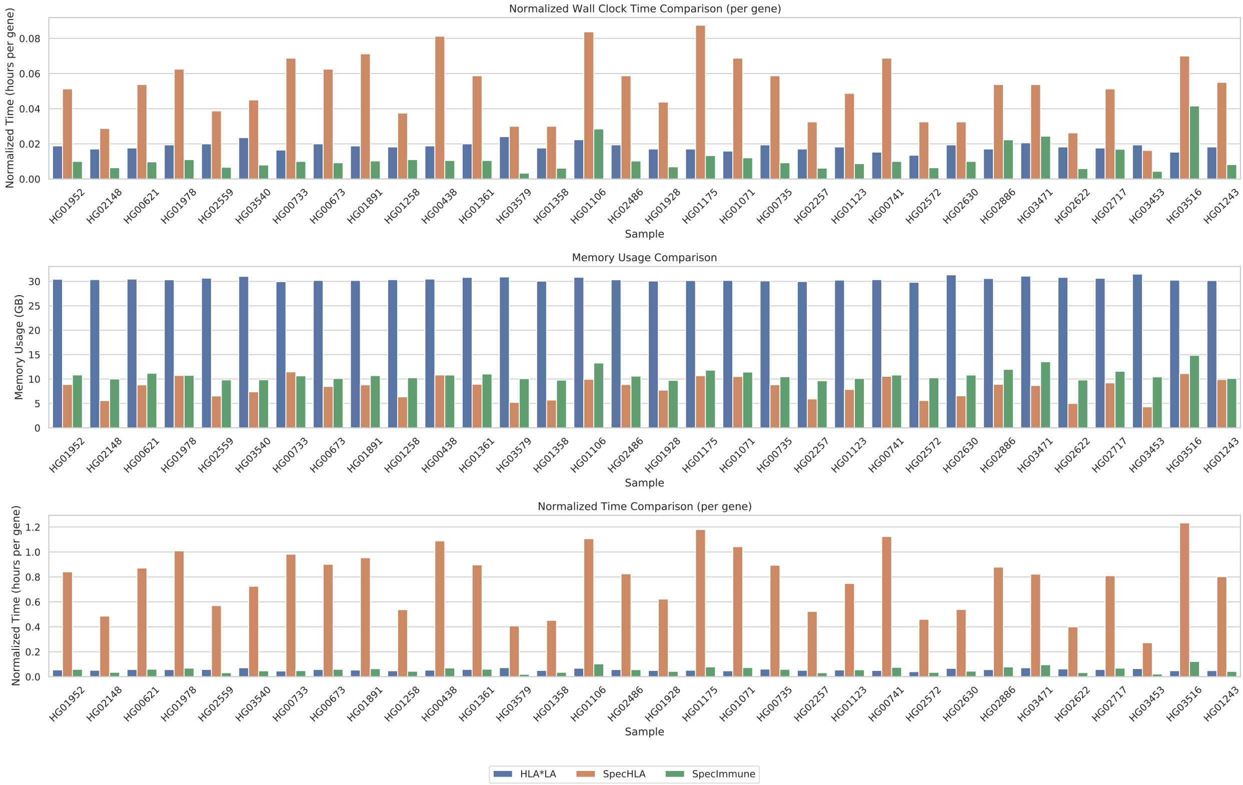

Supplementary Figure S4: Computational resource evaluation for HLA typing in HPRC HiFi samples.

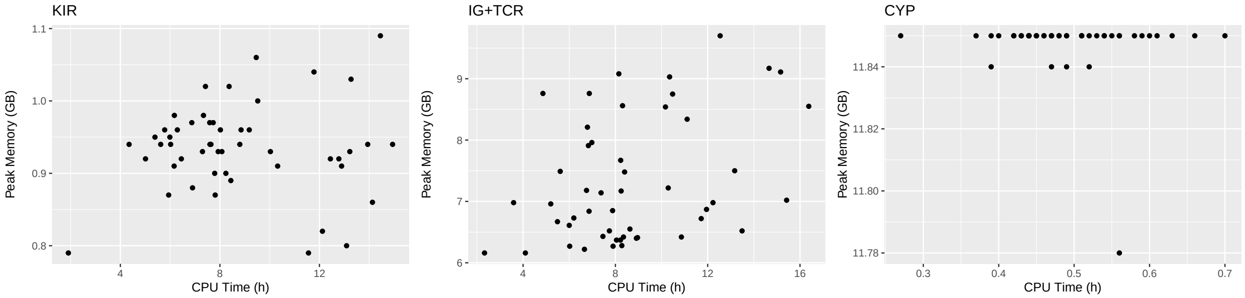

Supplementary Figure S5: Computational resource evaluation of SpecImmune for KIR, IG/TCR, and CYP typing on HPRC HiFi samples.

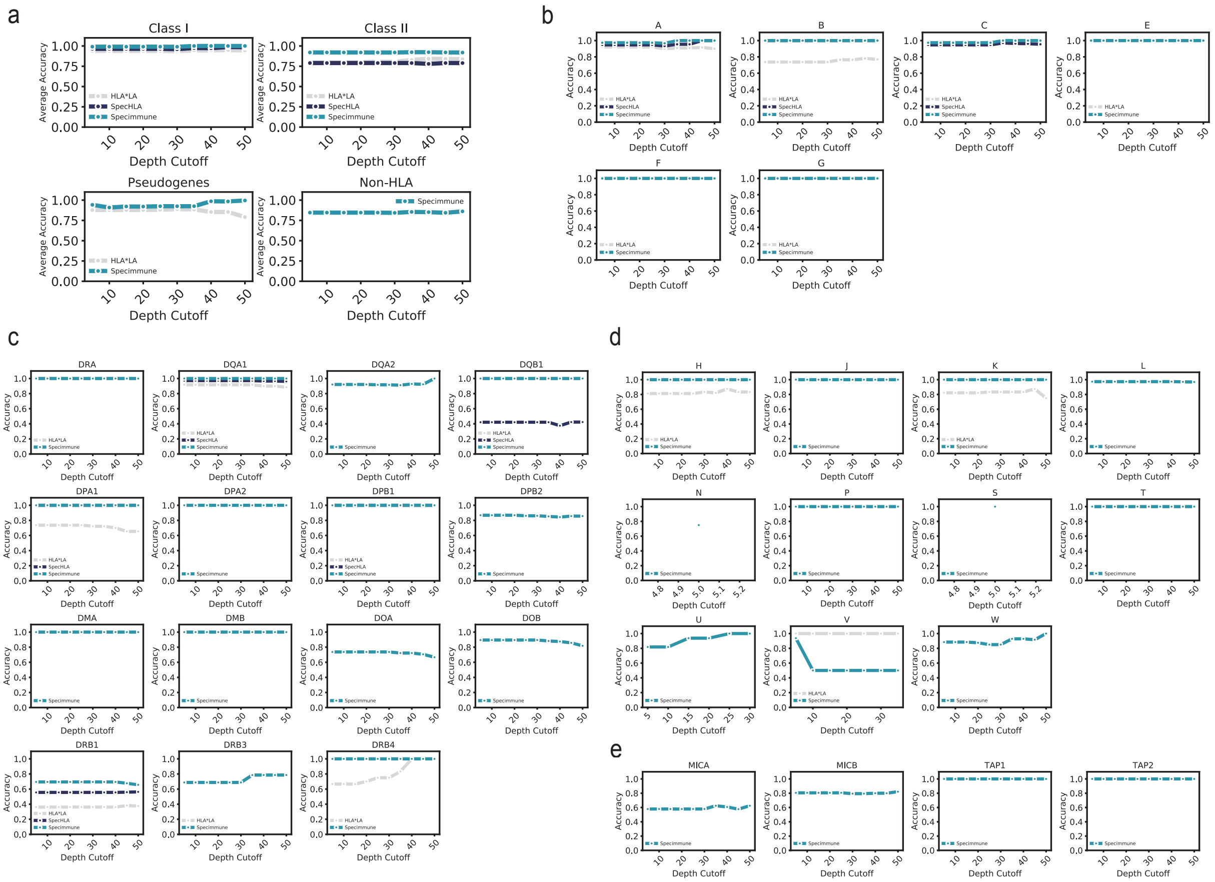

Supplementary Figure S6: Performance of HLA*LA, SpecHLA, and SpecImmune on HGSVC CLR dataset. (a) Accuracy of HLA*LA, SpecHLA, and SpecImmune of 4 HLA gene classes. (b) Accuracy of HLA*LA, SpecHLA, and SpecImmune of HLA class I genes. (c) Accuracy of HLA*LA, SpecHLA, and SpecImmune of HLA class II genes. (d) Accuracy of HLA*LA, SpecHLA, and SpecImmune of HLA Pseudogenes genes. (e) Accuracy of HLA*LA, SpecHLA, and SpecImmune of Non-HLA genes.

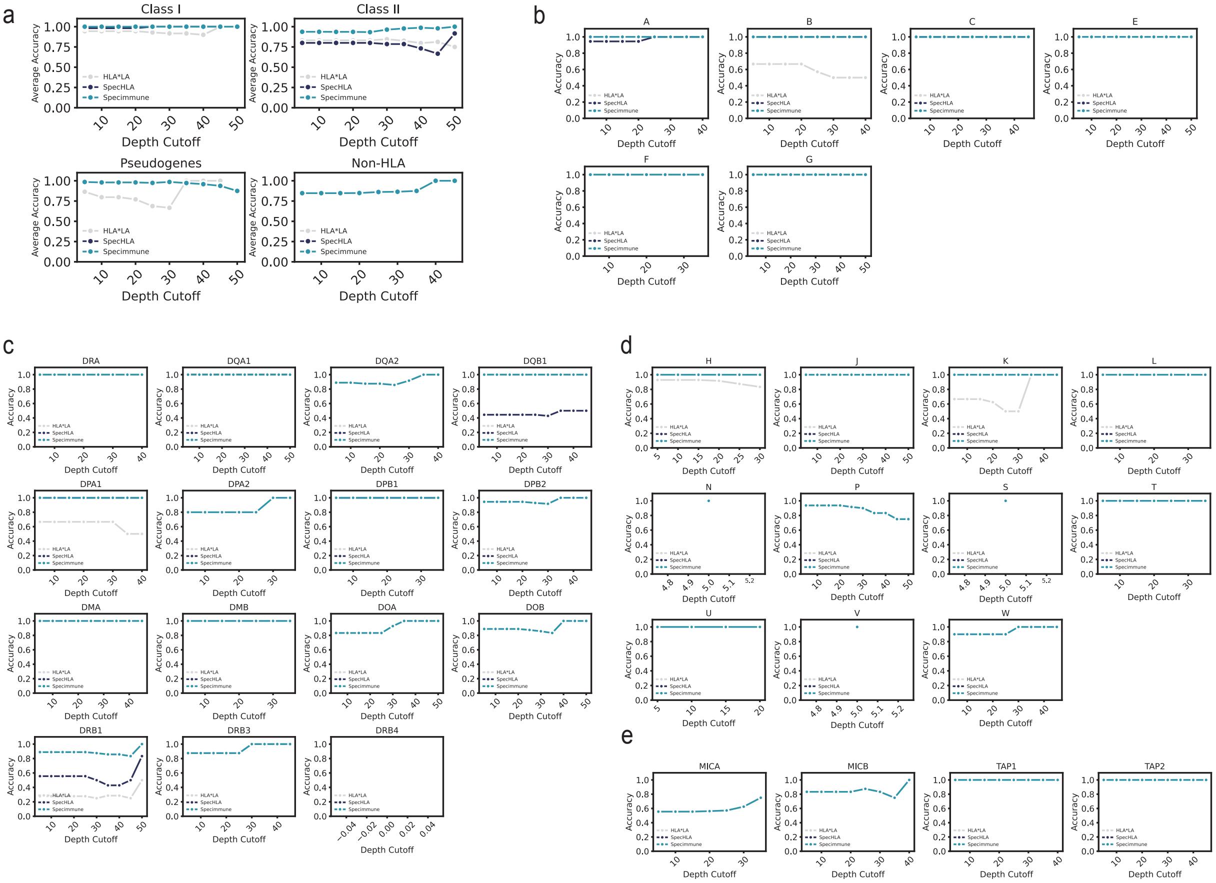

Supplementary Figure S7: Performance of HLA*LA, SpecHLA, and SpecImmune on HGSVC HiFi dataset. (a) Accuracy of HLA*LA, SpecHLA, and SpecImmune of 4 HLA gene classes. (b) Accuracy of HLA*LA, SpecHLA, and SpecImmune of HLA class I genes. (c) Accuracy of HLA*LA, SpecHLA, and SpecImmune of HLA class II genes. (d) Accuracy of HLA*LA, SpecHLA, and SpecImmune of HLA Pseudogenes genes. (e) Accuracy of HLA*LA, SpecHLA, and SpecImmune of Non-HLA genes.

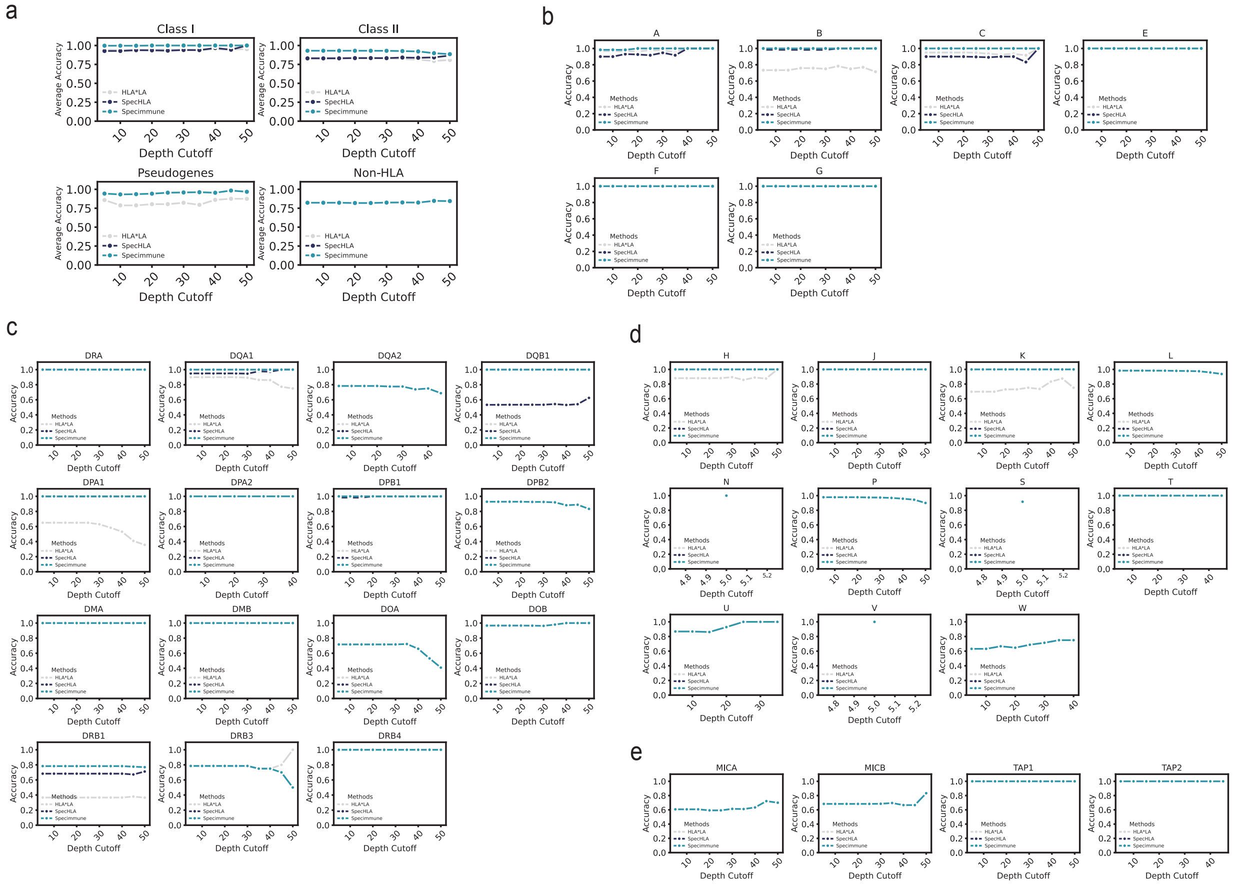

Supplementary Figure S8: Performance of HLA*LA, SpecHLA, and SpecImmune on HPRC HiFi dataset. (a) Accuracy of HLA*LA, SpecHLA, and SpecImmune of 4 HLA gene classes. (b) Accuracy of HLA*LA, SpecHLA, and SpecImmune of HLA class I genes. (c) Accuracy of HLA*LA, SpecHLA, and SpecImmune of HLA class II genes. (d) Accuracy of HLA*LA, SpecHLA, and SpecImmune of HLA Pseudogenes genes. (e) Accuracy of HLA*LA, SpecHLA, and SpecImmune of Non-HLA genes.

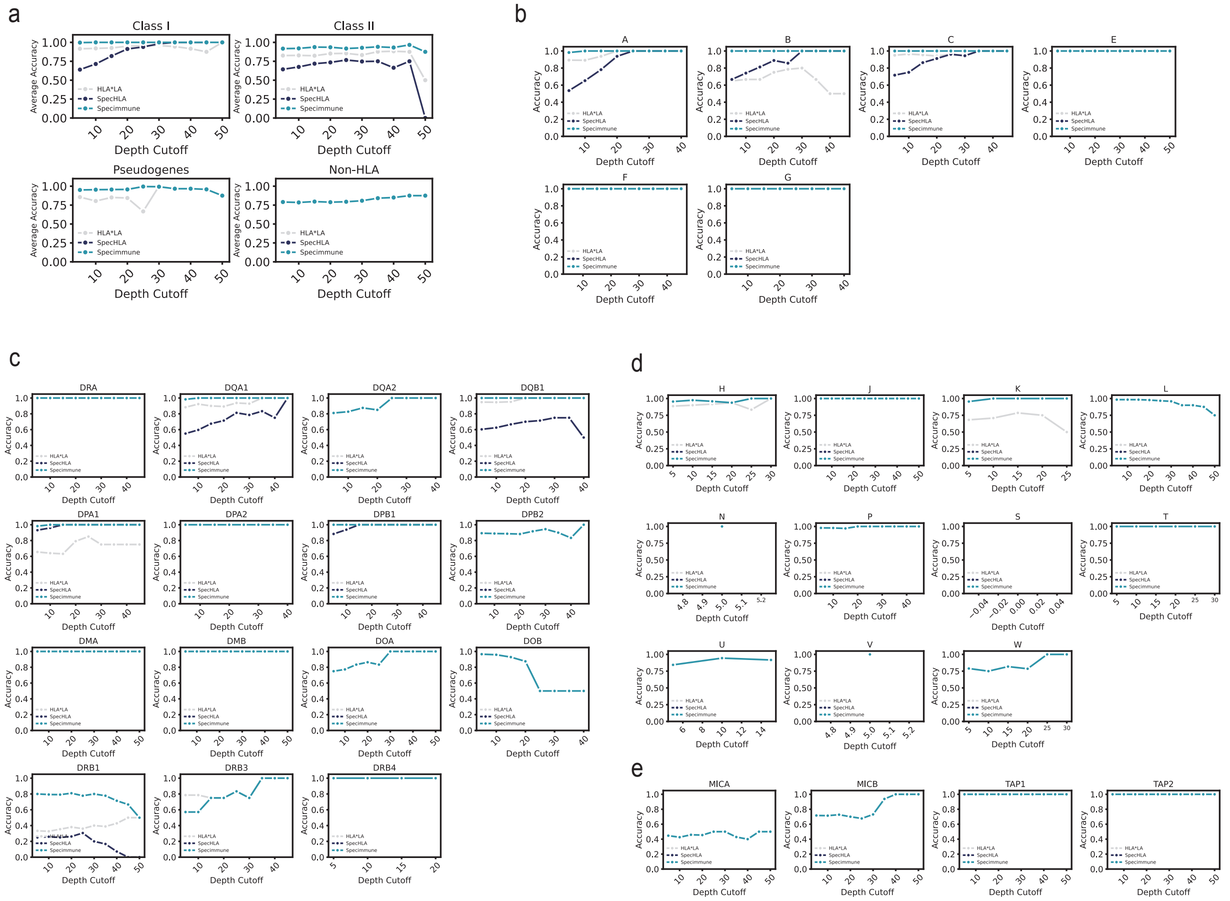

Supplementary Figure S9: Performance of HLA*LA, SpecHLA, and SpecImmune on HPRC ONT dataset. (a) Accuracy of HLA*LA, SpecHLA, and SpecImmune of 4 HLA gene classes. (b) Accuracy of HLA*LA, SpecHLA, and SpecImmune of HLA class I genes. (c) Accuracy of HLA*LA, SpecHLA, and SpecImmune of HLA class II genes. (d) Accuracy of HLA*LA, SpecHLA, and SpecImmune of HLA Pseudogenes genes. (e) Accuracy of HLA*LA, SpecHLA, and SpecImmune of Non-HLA genes.

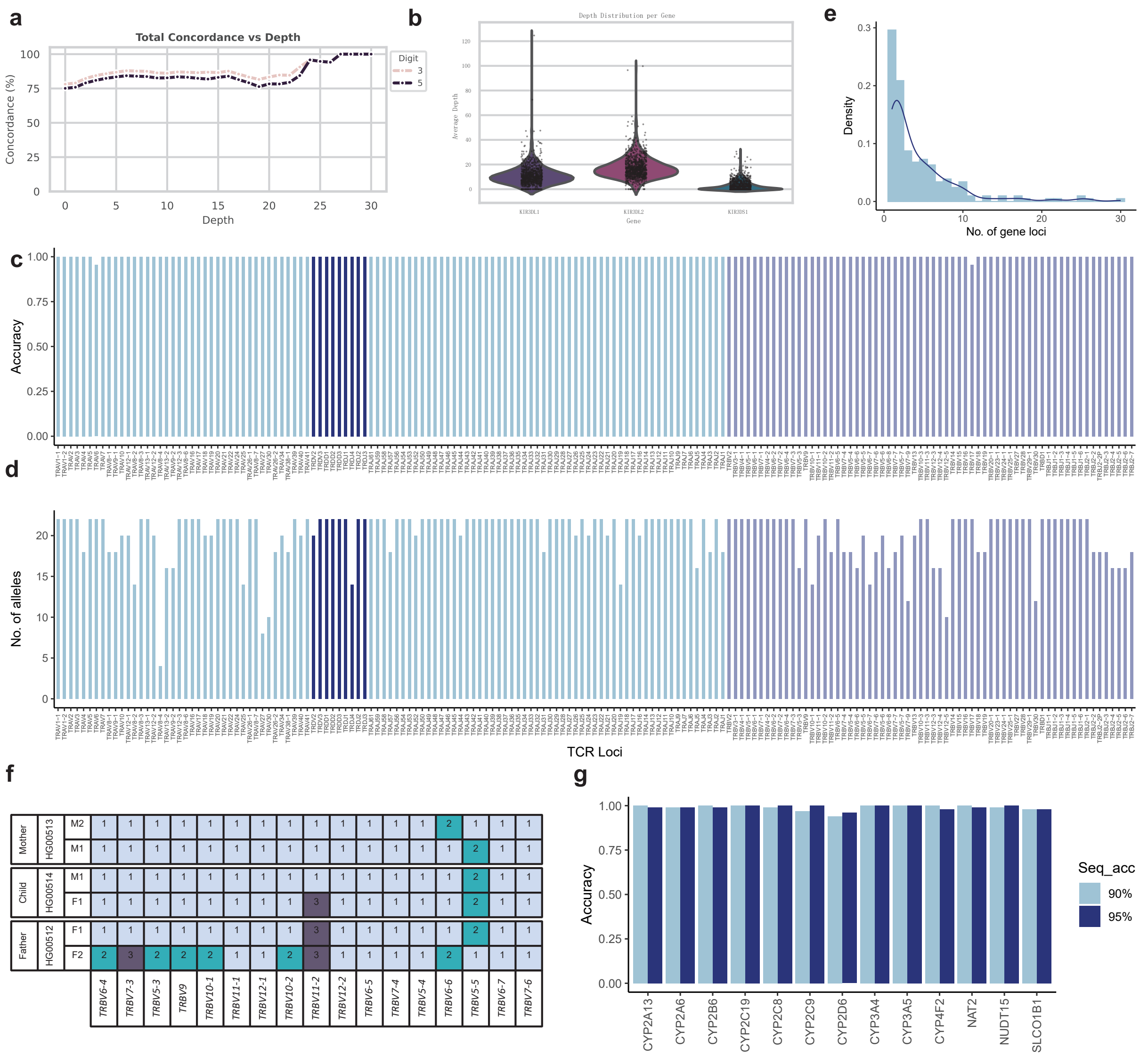

Supplementary Figure S10: Evaluation of SpecImmune for typing KIR, IG/TCR, CYP genes. (a) KIR typing concordance of SpecImmune and SKIRT in the HPRC dataset. (b) Depth distribution of the three low-depth KIR loci in the 1kGP ONT VIENNA cohort. (c-d) Accuracy (c) and number (d) of detected alleles at each TCR locus in the targeted TCR HiFi sequencing data. (e) Distribution of heterozygous gene loci count in each phase block. (f) Illustration of the phase block in two family trios. Numbers represent different alleles at each locus. (g) Accuracy of SpecImmune in typing different CYP loci using simulated data. Bar color indicates sequencing read accuracy at either 90% or 95%.

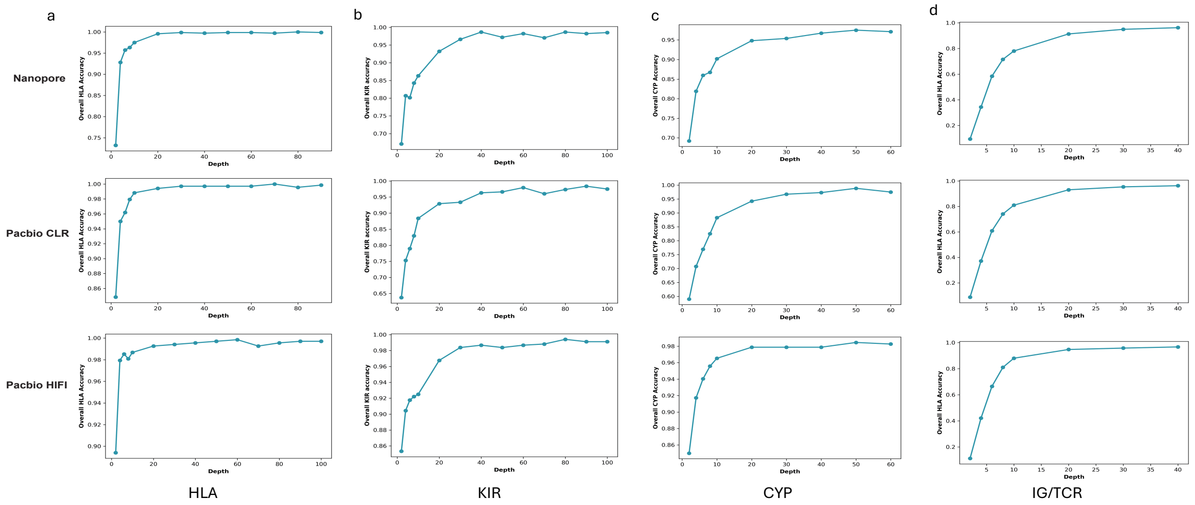

Supplementary Figure S11: Performance of SpecImmune across sequencing platforms and depths. Performance of SpecImmune for the HLA (a), KIR (b), CYP (c), and IG/TCR (d). The figure illustrates the performance of SpecImmune on three sequencing platforms: Nanopore, PacBio CLR, and PacBio HiFi, at varying sequencing depths.

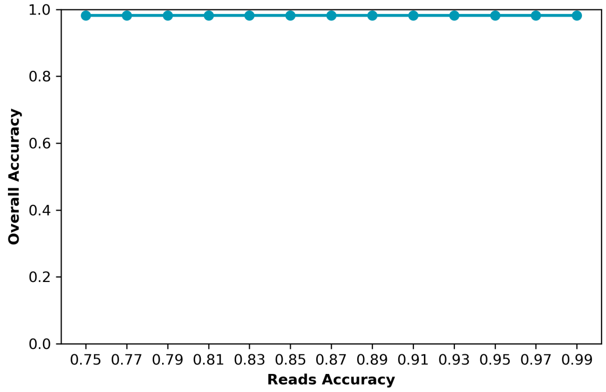

Supplementary Figure S12: SpecImmune performance for the HLA genes across sequencing reads accuracy.

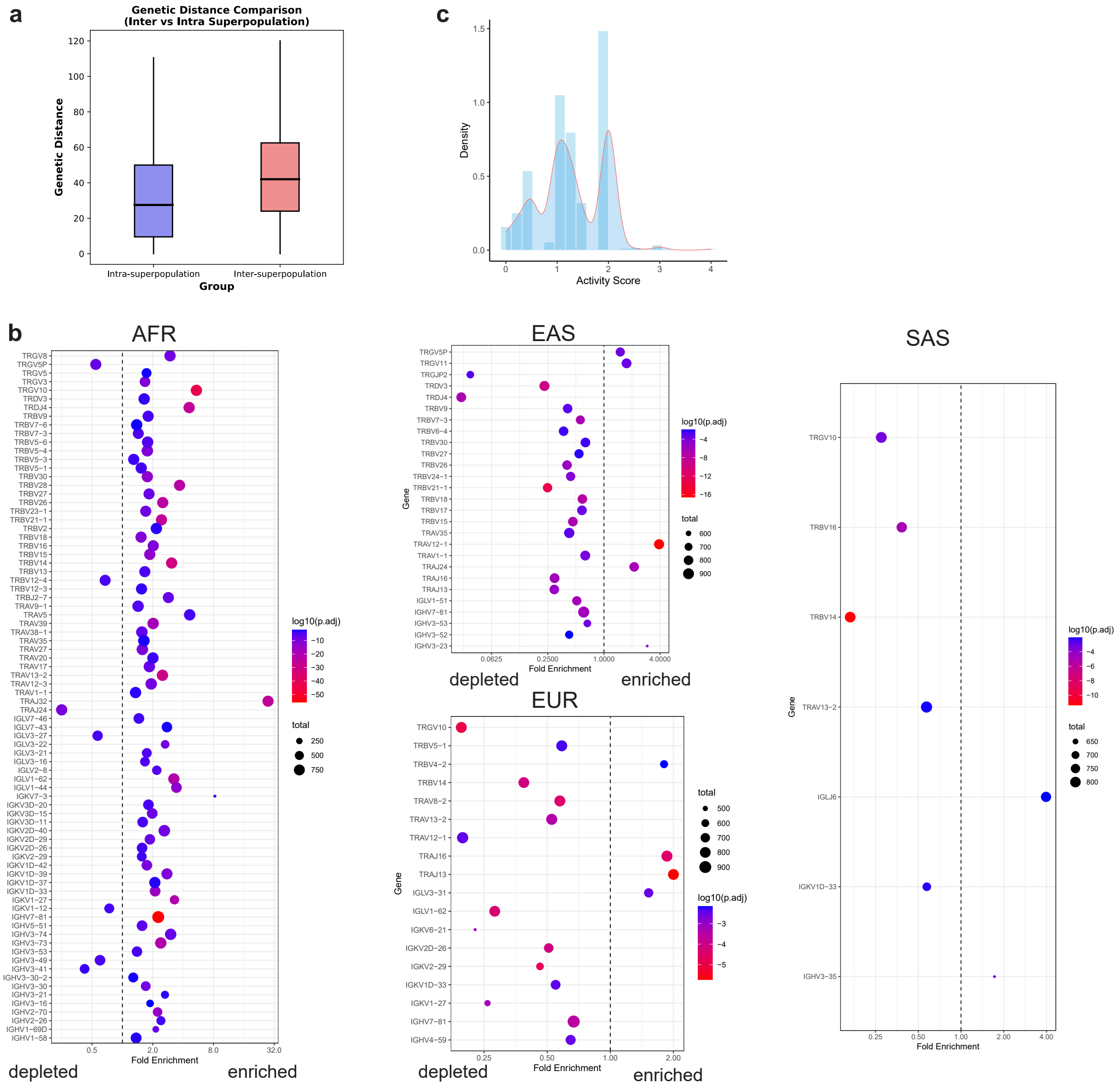

Supplementary Figure S13: Landscape of immune-related gene alleles in 1kGP population. (a) Comparison of HLA genetic distance within the same super populations and between different super populations. (b) Genes with enriched and depleted heterozygous variants in the populations. (c) Distribution of *CYP2D6* activity scores across all samples.

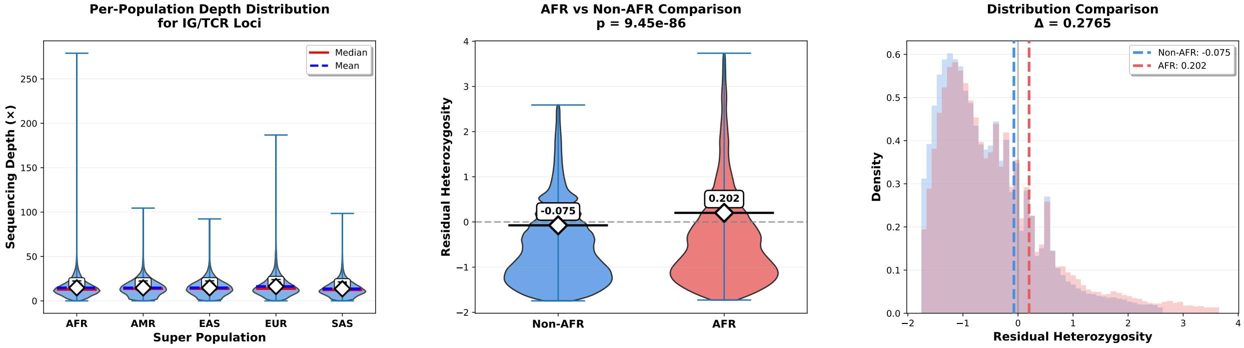

Supplementary Figure S14: Population differences in IG/TCR heterozygosity after controlling for technical covariates.

Multivariate linear regression was used to remove the effects of sequencing depth (log-transformed), read length, mapping quality, and basecaller mode from observed heterozygous variant counts across IG/TCR genes in 1kGP samples. (Left) Per-population distributions of sequencing depth for IG/TCR loci. (Center) Violin plot comparing residual heterozygosity between African (AFR, $n = 104{,}747$) and non-African populations (Non-AFR, $n = 283{,}170$). White diamonds indicate mean values, and horizontal black lines mark the mean position. (Right) Density distributions of residual heterozygosity for both groups. Dashed vertical lines indicate population means (Non-AFR: -0.075, AFR: 0.202). After accounting for all technical factors, AFR populations exhibit significantly higher IG/TCR heterozygosity compared to non-AFR populations ($\Delta = 0.2765$, t-test $p = 9.45 \times 10^{-86}$, Cohen's $d = 0.071$).

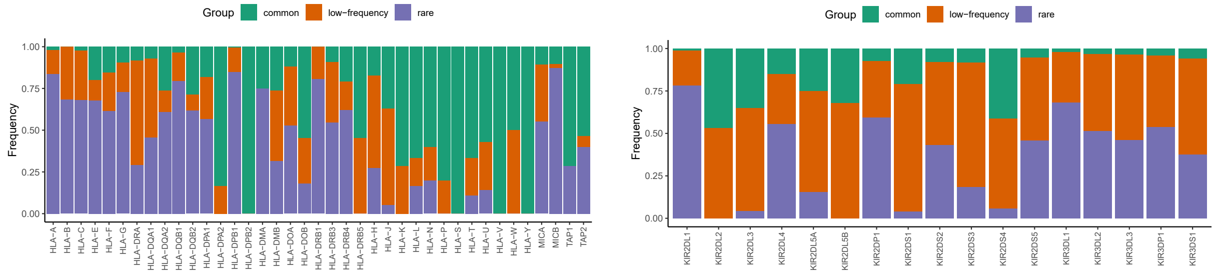

Supplementary Figure S15: Frequencies of common, low-frequency, and rare alleles at each HLA and KIR locus.

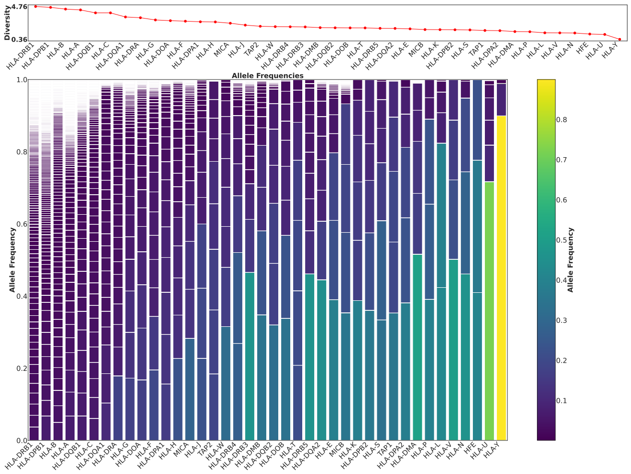

Supplementary Figure S16: Allelic diversity across HLA loci based on Shannon's original entropy formula. The top line plot shows the Shannon diversity index calculated using the maximum likelihood estimator (MLE) at each locus [13]. The bottom stacked bar plot displays the allele frequencies across loci.

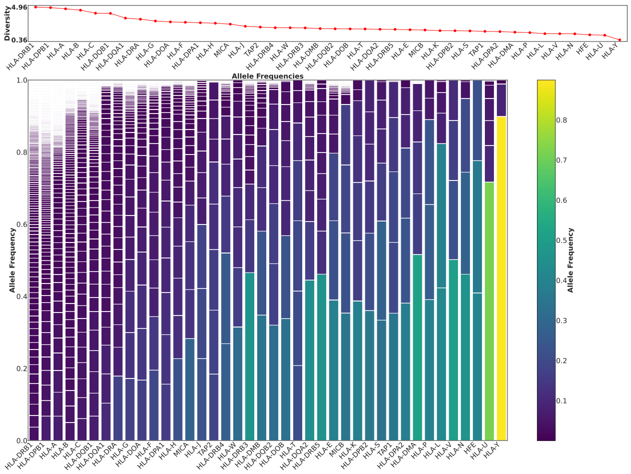

Supplementary Figure S17: Allelic diversity across HLA loci estimated using the unbiased Chao entropy estimator.

The top line plot shows Shannon diversity values corrected for unseen alleles using the nonparametric unbiased estimator proposed by Chao et al. (2013) [14]. The bottom stacked bar plot displays the observed allele frequencies at each locus.

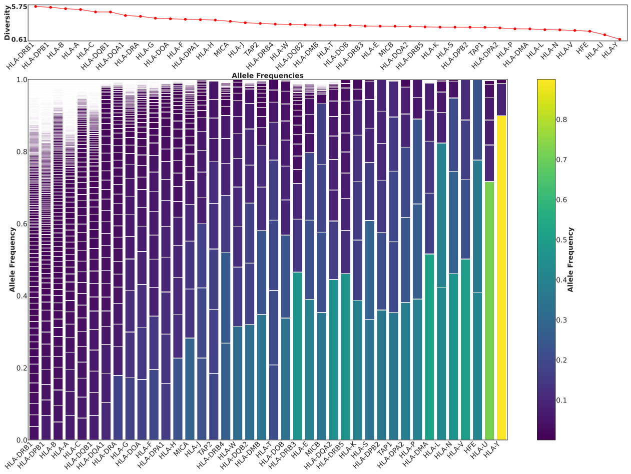

Supplementary Figure S18: Allelic diversity across HLA loci estimated using the jackknife Shannon entropy estimator.

The top line plot shows Shannon diversity values estimated using the first-order jackknife correction method described by Zahl (1977) [15], which reduces bias from limited sampling. The bottom stacked bar plot displays the observed allele frequencies at each locus.

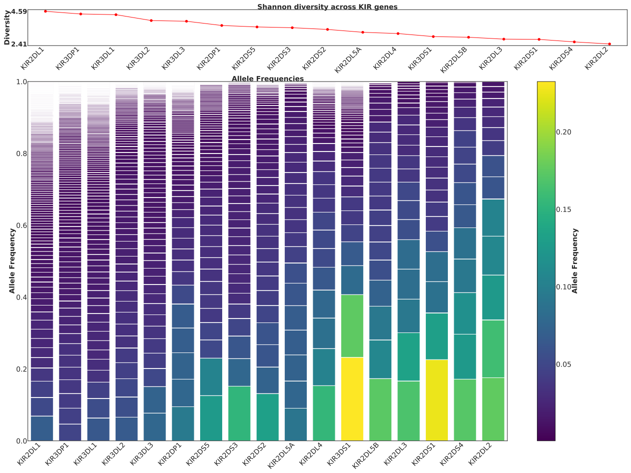

Supplementary Figure S19: Allelic diversity across KIR loci based on Shannon's original entropy formula. The top line plot shows the Shannon diversity index calculated using the maximum likelihood estimator (MLE) at each locus [13]. The bottom stacked bar plot displays the allele frequencies across loci.

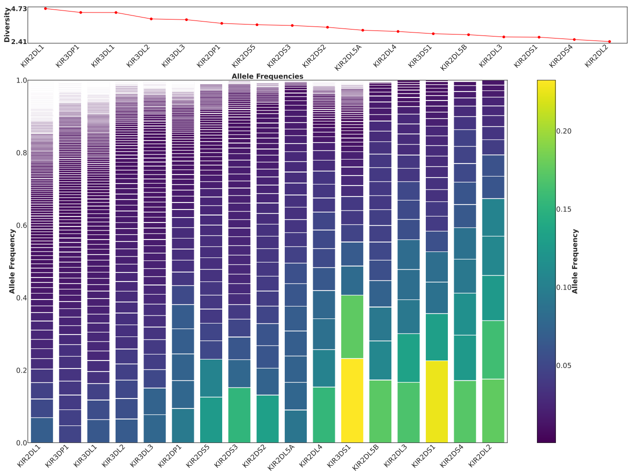

Supplementary Figure S20: Allelic diversity across KIR loci estimated using the unbiased Chao entropy estimator.

The top line plot shows the Shannon diversity index calculated using the maximum likelihood estimator (MLE) at each locus [13]. The bottom stacked bar plot displays the allele frequencies across loci.

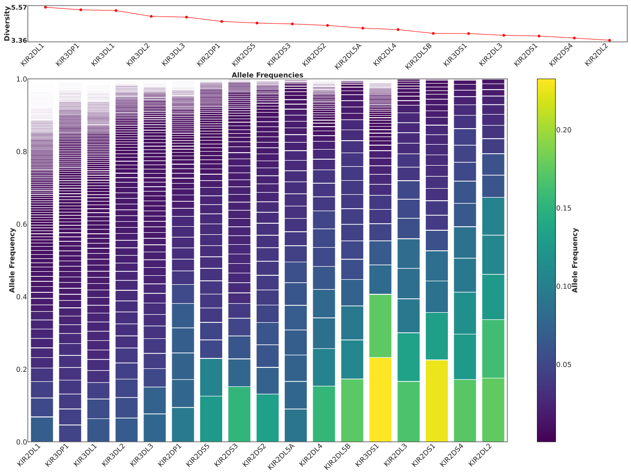

Supplementary Figure S21: Allelic diversity across KIR loci estimated using the jackknife Shannon entropy estimator.

The top line plot shows the Shannon diversity index calculated using the maximum likelihood estimator (MLE) at each locus [13]. The bottom stacked bar plot displays the allele frequencies across loci.

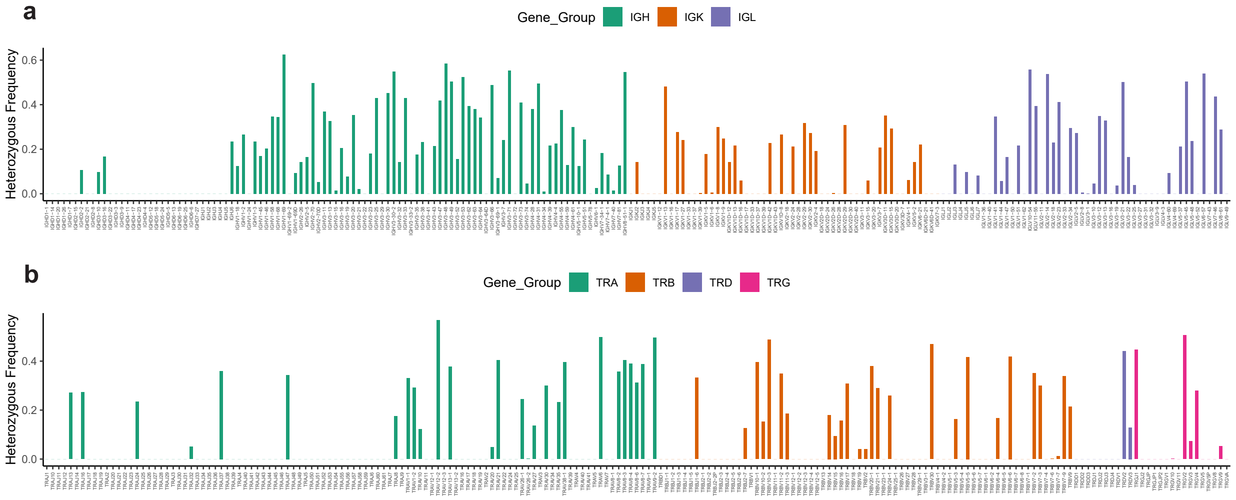

Supplementary Figure S22: Heterozygous frequencies at IG and TCR loci.
(a–b) Heterozygous frequencies at each IG (a) and TCR (b) locus.

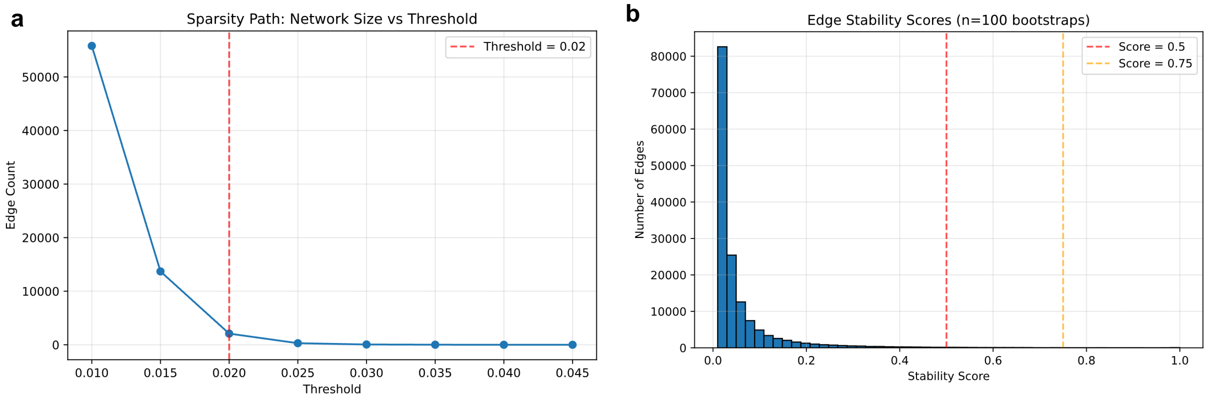

Supplementary Figure S23: Sparsity and stability of precision-matrix–derived networks.
(a) Number of edges retained in the allele association graph as a function of the absolute precision threshold, illustrating the sparsity path of the inverse-covariance network. The dashed line indicates the threshold ($|\Theta_{ij}| = 0.02$) used for subsequent stability selection. (b) Distribution of edge stability scores obtained from 100 bootstrap resamples of populations. Stability is defined as the fraction of resamples in which an edge exceeds the precision threshold. Dashed lines indicate stability cutoffs of 0.5 and 0.75; edges with stability ≥ 0.75 were retained in the final high-confidence network.

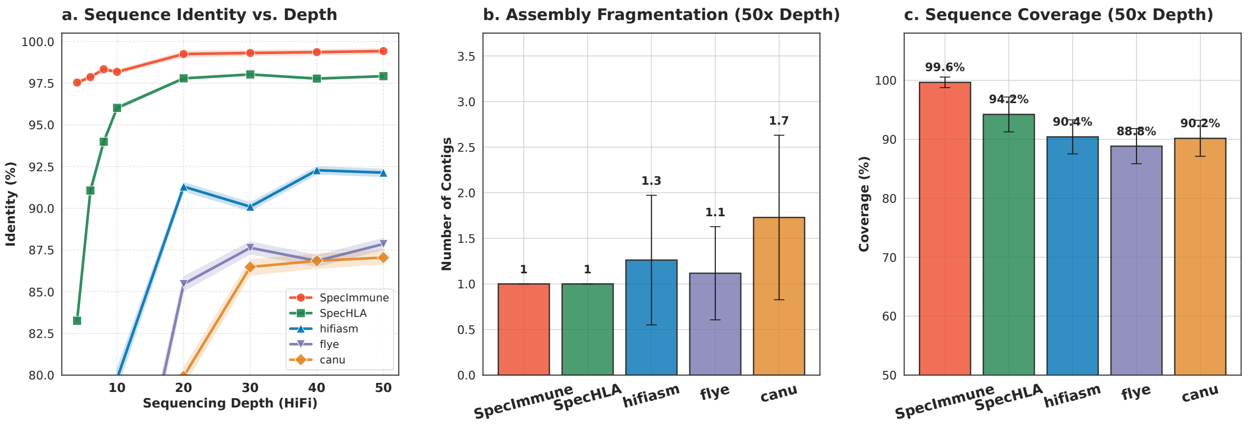

Supplementary Figure S24: Performance comparison of HLA assembly tools on novel alleles from IMGT/HLA-3.62.0.

The figure evaluates the assembly quality of SpecImmune, SpecHLA, and three *de novo* assemblers (Hifiasm, Flye, Canu) when reconstructing novel HLA alleles. **(a)** Sequence identity between the assembled contigs and the ground truth reference sequences across varying sequencing depths (4x, 6x, 8x, 10x, 20x, 30x, 40x, and 50x). Higher values indicate higher base-level accuracy. **(b)** The average number of contigs generated per haplotype. A value closer to 1 indicates a more contiguous and less fragmented assembly. **(c)** The alignment coverage of the assembled sequences against the true reference alleles. This metric reflects the completeness of the assembly in capturing the full length of the novel alleles.

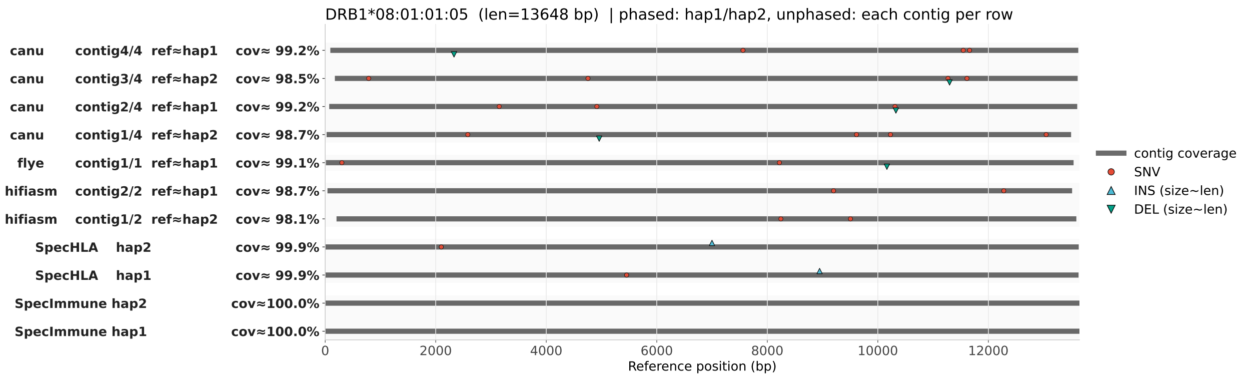

Supplementary Figure S25: Comparison of assembled sequences for the *HLA-DRB1*08:01:01:05* allele across different software tools.

The visualization displays the sequence alignment and structural consistency of the *DRB1*08:01:01:05* allele as reconstructed by SpecImmune, SpecHLA, Hifiasm, Flye, and Canu.

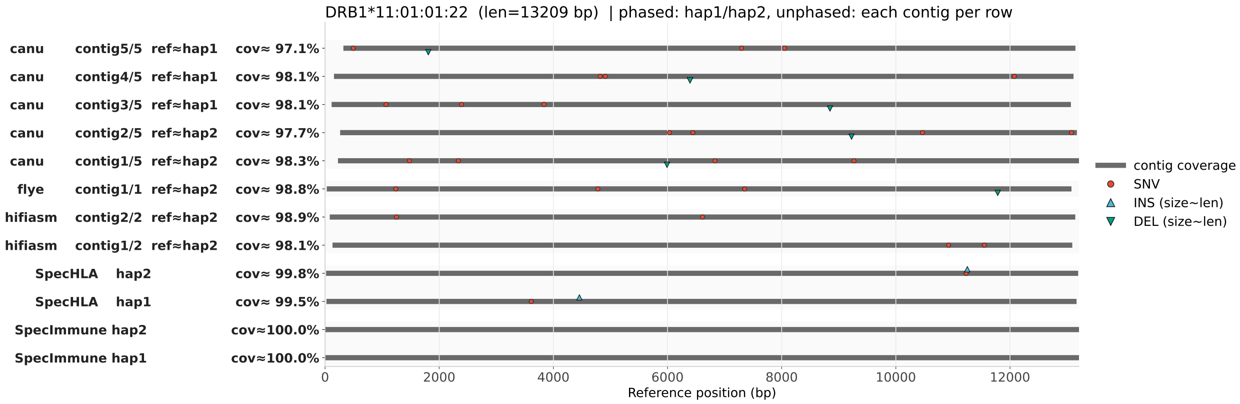

Supplementary Figure S26: Comparison of assembled sequences for the *HLA-DRB1*11:01:01:22* allele across different software tools.

The visualization displays the sequence alignment and structural consistency of the *HLA-DRB1*11:01:01:22* allele as reconstructed by SpecImmune, SpecHLA, Hifiasm, Flye, and Canu.

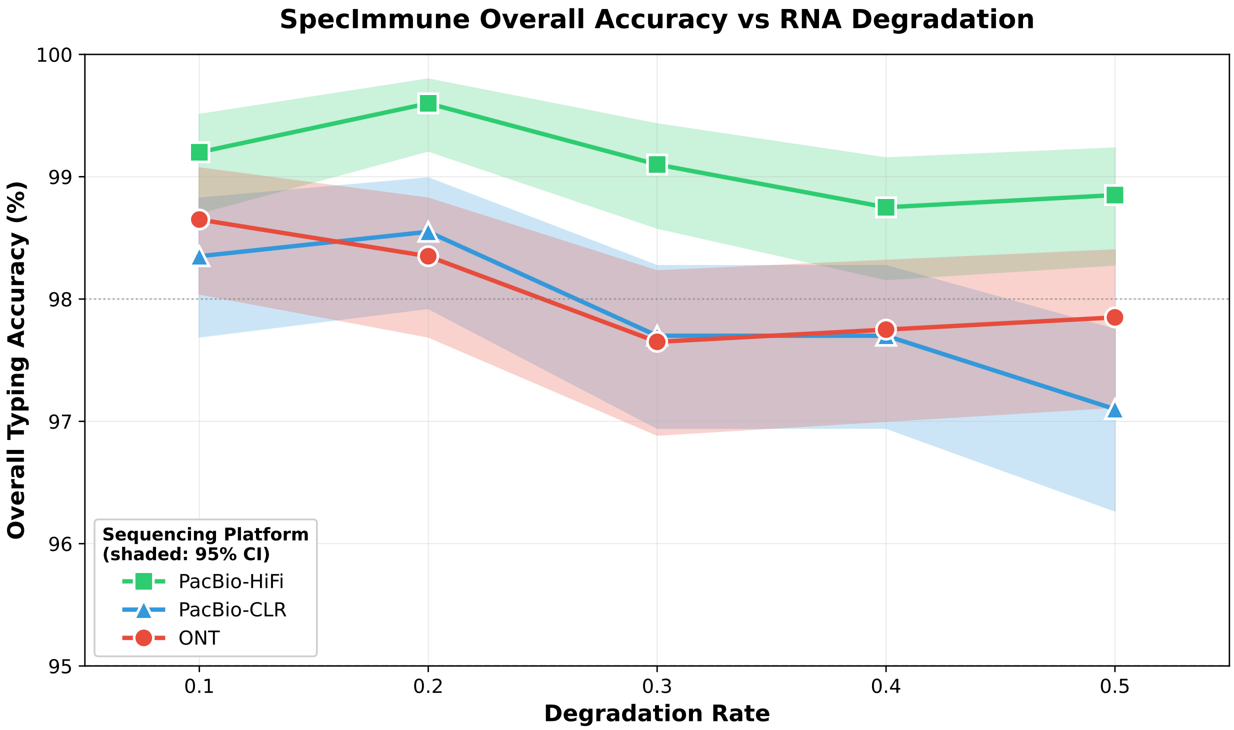

Supplementary Figure S27: Robustness of SpecImmune to transcriptomic fragmentation across long-read platforms.

Performance was evaluated on PacBio HiFi, CLR, and ONT platforms under varying RNA degradation coefficients (0.1–0.5) using $n = 500$ simulated replicates. SpecImmune demonstrated exceptional resilience, maintaining a global average accuracy of 98.34%. Notably, the PacBio HiFi platform sustained 98.85% accuracy even under severe degradation conditions, while CLR and ONT platforms exhibited negligible performance declines ($< 1.3\%$).

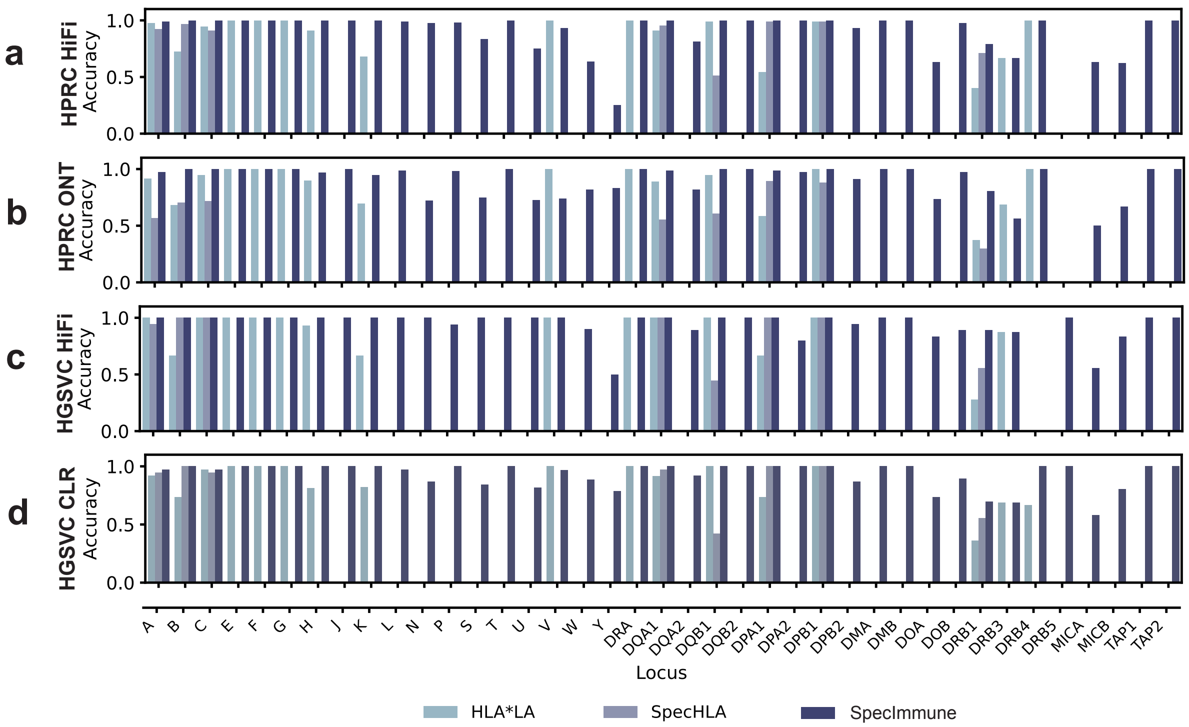

Supplementary Figure S28: Evaluation of SpecImmune for full typing HLA genes.
(a–d) Accuracy comparisons among SpecHLA, HLA*LA, and SpecImmune, across HLA loci of HLA*LA, SpecHLA and SpecImmune for the HPRC HiFi (a), HPRC ONT (b), HGSVC HiFi (c), and HGSVC CLR (d) datasets, respectively.

Supplementary Table S1: Commands used for SpecHLA, HLA*LA, and SpecImmune in evaluation.

Software	Command	Gene Family
**SpecHLA V1.0.6**	`python3 SpecHLA/script/long_read_typing.py -r $fastq -j 20 -n $sample_id -o $outdir -y $datatype`	HLA
**HLA*LA V1.0.4**	`HLA-LA/src/HLA-LA.pl --BAM $sample_bam --graph PRG_MHC_GRCh38_withIMGT --sampleID $sample_id --maxThreads 20 --longReads $datatype --samtools_T $REFERENCE_GENOME --workingDir $outdir`	HLA
**SpecImmune V0.0.1**	`python3 SpecImmune/main.py -r $fastq -j 20 -i HLA -n $sample_id -o $outdir --db SpecImmune/db -y $datatype --align_method_1 minimap2`	HLA
**SpecImmune V0.0.1**	`python3 SpecImmune/main.py -r $fastq -j 20 -i KIR -n $sample_id -o $outdir --db SpecImmune/db -y $datatype --align_method_1 minimap2 --hete_p 0.2`	KIR
**SpecImmune V0.0.1**	`python3 SpecImmune/main.py -r $fastq -j 20 -i CYP -n $sample_id -o $outdir --db SpecImmune/db -y $datatype`	CYP
**SpecImmune V0.0.1**	`python3 SpecImmune/main.py -r $fastq -j 20 -i IG_TR -n $sample_id -o $outdir --db SpecImmune/db -y $datatype --hg38 $no_alt_ref`	IG and TCR

Supplementary Table S2: Trio information in 1kGP

Trio ID	Child	Father	Mother
2418	NA19828	NA19818	NA19819
CLM16	HG01258	HG01256	HG01257
1463-Paternal	NA12877	NA12889	NA12890
1463-Maternal	NA12878	NA12891	NA12892
SH006	HG00420	HG00418	HG00419
Y077	NA19129	NA19128	NA19127

Supplementary Table S3: Recommended Read-Depth Thresholds for Specimmune

Gene Family	PacBio CLR	PacBio HiFi	ONT
HLA	40×	30×	40×
KIR	40×	30×	40×
CYP	30×	20×	30×
IG/TCR	30×	20×	30×

Supplementary Table S4: HLA Genotyping Accuracies in 1kGP

Gene	SpecImmune		SpecHLA		HLA*LA	
	2-Field	G/P group	2-Field	G/P group	2-Field	G/P group
HLA-A	94% (1,134/1,208)	95% (1,144/1,208)	82% (996/1,208)	83% (1,005/1,208)	85% (1,028/1,208)	93% (1,125/1,208)
HLA-B	89% (1,402/1,568)	92% (1,438/1,568)	72% (1,133/1,568)	73% (1,145/1,568)	84% (1,323/1,568)	92% (1,444/1,568)
HLA-C	98% (1,561/1,590)	98% (1,563/1,590)	85% (1,357/1,590)	91% (1,443/1,590)	84% (1,336/1,590)	92% (1,470/1,590)
HLA-DQB1	85% (1,209/1,422)	86% (1,222/1,422)	57% (812/1,422)	59% (834/1,422)	76% (1,085/1,422)	82% (1,170/1,422)
HLA-DRB1	73% (1,348/1,858)	73% (1,360/1,858)	59% (1,100/1,858)	60% (1,108/1,858)	70% (1,296/1,858)	76% (1,410/1,858)

References

[1] Fusheng Zhou, Hongzhi Cao, Xianbo Zuo, Tao Zhang, Xiaoguang Zhang, Xiaomin Liu, Ricong Xu, Gang Chen, Yuanwei Zhang, Xiaodong Zheng, et al. Deep sequencing of the mhc region in the chinese population contributes to studies of complex disease. *Nature genetics*, 48(7):740–746, 2016.

[2] Diego Chowell, Chirag Krishna, Federica Pierini, Vladimir Makarov, Naiyer A Rizvi, Fengshen Kuo, Luc GT Morris, Nadeem Riaz, Tobias L Lenz, and Timothy A Chan. Evolutionary divergence of hla class i genotype impacts efficacy of cancer immunotherapy. *Nature medicine*, 25(11):1715–1720, 2019.

[3] Richard M Single, Maureen P Martin, Xiaojiang Gao, Diogo Meyer, Meredith Yeager, Judith R Kidd, Kenneth K Kidd, and Mary Carrington. Global diversity and evidence for coevolution of kir and hla. *Nature genetics*, 39(9):1114–1119, 2007.

[4] Shuai Wang, Mengyao Wang, Lingxi Chen, Guangze Pan, Yanfei Wang, and Shuai Cheng Li. Spechla enables full-resolution hla typing from sequencing data. *Cell Reports Methods*, 3(9), 2023.

[5] Sergey Koren, Brian P Walenz, Konstantin Berlin, Jason R Miller, Nicholas H Bergman, and Adam M Phillippy. Canu: scalable and accurate long-read assembly via adaptive k-mer weighting and repeat separation. *Genome Res.*, 27(5):722–736, May 2017.

[6] Mikhail Kolmogorov, Jeffrey Yuan, Yu Lin, and Pavel A. Pevzner. Assembly of long, error-prone reads using repeat graphs. *Nature Biotechnology*, 37(5):540–546, May 2019. ISSN 1546-1696. doi: 10.1038/s41587-019-0072-8. URL https://doi.org/10.1038/s41587-019-0072-8.

[7] Haoyu Cheng, Gregory T. Concepcion, Xiaowen Feng, Haowen Zhang, and Heng Li. Haplotype-resolved de novo assembly using phased assembly graphs with hifiasm. *Nature Methods*, 18(2):170–175, Feb 2021. ISSN 1548-7105. doi: 10.1038/s41592-020-01056-5. URL https://doi.org/10.1038/s41592-020-01056-5.

[8] Véronique Giudicelli, Xavier Brochet, and Marie-Paule Lefranc. Imgt/v-quest: Imgt standardized analysis of the immunoglobulin (ig) and t cell receptor (tr) nucleotide sequences. *Cold Spring Harbor Protocols*, 2011(6):pdb–prot5633, 2011.

[9] Alexander T Dilthey, Pierre-Antoine Gourraud, Alexander J Mentzer, Nezih Cereb, Zamin Iqbal, and Gil McVean. High-accuracy hla type inference from whole-genome sequencing data using population reference graphs. *PLoS Comput. Biol.*, 12(10):e1005151, 2016.

[10] Dominic J Barker, Giuseppe Maccari, Xenia Georgiou, Michael A Cooper, Paul Flicek, James Robinson, and Steven GE Marsh. The ipd-imgt/hla database. *Nucleic acids research*, 51(D1):D1053–D1060, 2023.

[11] James Robinson, Jason A Halliwell, Hamish McWilliam, Rodrigo Lopez, and Steven GE Marsh. Ipd—the immuno polymorphism database. *Nucleic acids research*, 41(D1):D1234–D1240, 2012.

[12] Andrea Gaedigk, Scott T Casey, Michelle Whirl-Carrillo, Neil A Miller, and Teri E Klein. Pharmvar: a global resource and repository for pharmacogene variation. *Clinical pharmacology and therapeutics*, 110(3):542, 2021.

[13] Maciej K Konopiński. Shannon diversity index: a call to replace the original shannon's formula with unbiased estimator in the population genetics studies. *PeerJ*, 8(e9391):e9391, June 2020.

[14] Anne Chao, Y. T. Wang, and Lou Jost. Entropy and the species accumulation curve: a novel entropy estimator via discovery rates of new species. *Methods in Ecology and Evolution*, 4(11):1091–1100, 2013. doi: https://doi.org/10.1111/2041-210X.12108. URL https://besjournals.onlinelibrary.wiley.com/doi/abs/10.1111/2041-210X.12108.

[15] Samuel Zahl. Jackknifing an index of diversity. *Ecology*, 58(4):907–913, 1977. doi: https://doi.org/10.2307/1936227. URL https://esajournals.onlinelibrary.wiley.com/doi/abs/10.2307/1936227.
